# Mitochondrial Carrier SLC25A13 Drives Ferroptosis Resistance and Immune Evasion via a STAT3–IFI6 Circuit in Breast Cancer

**DOI:** 10.1002/advs.75818

**Published:** 2026-05-25

**Authors:** Yingze Zhu, Yuhan Tang, Yingcui Chen, Zhuoqi Zhang, Yige Lu, Xinyue Wang, Qiyuan Shi, Wenhui Zhao, Hui Pang

**Affiliations:** ^1^ The Department of Medical Oncology Harbin Medical University Cancer Hospital Harbin Heilongjiang P. R. China; ^2^ Clinical College Shanghai Jiaotong University Shanghai P. R. China

## Abstract

Triple‐negative breast cancer (TNBC) remains poorly responsive to immunotherapy, and how ferroptosis can be leveraged to enhance antitumor immunity is unclear. Here, we identify the mitochondrial aspartate/glutamate carrier SLC25A13 as a key immunometabolic driver in TNBC. SLC25A13 is upregulated in breast cancer, predicts poor prognosis, and is associated with reduced CD8^+^ T‐cell infiltration. Functionally, SLC25A13 promotes tumor growth, migration, and metastasis while suppressing ferroptosis and weakening CD8^+^ T‐cell‐mediated cytotoxicity. Mechanistically, SLC25A13 interacts with STAT3, enhances complex I‐linked oxidative phosphorylation, restrains mitochondrial ROS, and promotes STAT3 activation and nuclear translocation. Nuclear STAT3 directly induces IFI6, which preserves mitochondrial function, limits lipid peroxidation and Fe^2^^+^ accumulation, and thereby confers ferroptosis resistance and immune evasion. Through structure‐guided screening, we identified HY‐QS02682823 as a small‐molecule degrader of SLC25A13 that triggers lysosome‐dependent SLC25A13 loss, enhances ferroptosis, restores CD8^+^ T‐cell effector function, and improves the efficacy of anti‐PD‐1 therapy in syngeneic TNBC models. These findings identify the SLC25A13–STAT3–IFI6 axis as a key regulator of ferroptosis resistance and immune evasion in TNBC.

## Graphical abstract

SLC25A13 is identified as an immunometabolic driver of triple‐negative breast cancer that sustains ferroptosis resistance and immune evasion through a STAT3–IFI6 circuit. Pharmacologic degradation of SLC25A13 restores ferroptosis sensitivity and enhances anti‐PD‐1 efficacy, highlighting a strategy to convert immune‐cold tumors into immunotherapy‐responsive disease.

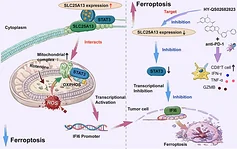

## 1 Introduction

Cancer immunotherapy—particularly immune checkpoint blockade (ICB) and chimeric antigen receptor (CAR) T cell therapy—has reshaped the treatment landscape and brought renewed hope to patients with advanced disease [1, 2, 3]. Much of their efficacy ultimately depends on reactivating and strengthening CD8^+^ cytotoxic T cells within the tumor microenvironment (TME) [4, 5]. These cells are the terminal effectors of tumor eradication. Activated CD8^+^ T cells can induce tumor cell death through several mechanisms. These include apoptosis, necroptosis, and ferroptosis, a recently recognized form of lipid peroxidation‐driven cell death [6, 7].

The mitochondrial carrier family (SLC25) forms the principal conduit linking metabolic pathways across the inner mitochondrial membrane. Among them, the mitochondrial aspartate/glutamate carrier SLC25A13 (also known as citrin) transports aspartate and glutamate to support the malate‐aspartate shuttle and the urea cycle, thereby safeguarding cellular energy production and nitrogen homeostasis [8]. Early studies mainly associated SLC25A13 with inherited metabolic disorders such as citrin deficiency. More recently, multi‐omics analyses have revealed that SLC25A13 is broadly upregulated in multiple malignancies, including hepatocellular, lung, and colorectal cancers, and its high expression correlates with enhanced tumor growth, invasion, and poor prognosis [9, 10, 11]. These observations suggest that SLC25A13 is not merely a passive “metabolic transporter” but may act as a proto‐oncogene that drives metabolic reprogramming in cancer cells [12, 13]. Notably, SLC25A13 expression is tightly linked to the tumor immune microenvironment, with elevated levels frequently coinciding with reduced infiltration of CD8^+^ cytotoxic T cells across cohorts. However, how SLC25A13 mechanistically shapes tumor cell‐immune cell crosstalk, and in particular how it modulates tumor susceptibility to cytotoxic attack, remains largely unexplored.

Ferroptosis is an iron‐dependent form of regulated cell death characterized by unchecked peroxidation of polyunsaturated phospholipids [14, 15]. Its execution reflects a dynamic balance between pro‐ferroptotic cues—such as labile Fe^2^^+^ accumulation and lipid reactive oxygen species—and anti‐ferroptotic defense systems, typified by the SLC7A11–GSH–GPX4 axis [16, 17]. Increasing evidence indicates that ferroptosis is not only a mode of tumor cell demise but also an important effector mechanism of CD8^+^ T‐cell‐mediated antitumor immunity [18]. Conversely, tumor cells often acquire ferroptosis resistance by engaging multiple metabolism‐linked defense circuits, including boosting glutathione synthesis, rewiring lipid and iron metabolism, and upregulating stress‐adaptive factors such as IFI6. Among these, IFI6 has emerged as a stress‐responsive molecule with pleiotropic roles in cancer biology [19, 20]. Previous studies suggest that IFI6 supports tumor‐cell survival by preserving mitochondrial homeostasis and adapting to oxidative stress, and its dysregulation has been linked to malignant progression in several tumor types. Emerging evidence also suggests that elevated IFI6 expression may be associated with an immunosuppressive tumor context in some malignancies. However, most current studies have focused on plasma‐membrane ferroptosis regulators such as system Xc^−^ and downstream antioxidant modules, whereas much less is known about how mitochondrial transport programs interface with IFI6‐dependent ferroptosis resistance and antitumor immunity [23]. These observations raise the possibility that IFI6 may function not only as a downstream transcriptional target, but also as a biologically relevant effector linking mitochondrial adaptation to ferroptosis control in cancer [21, 22].

Although ferroptosis induction has attracted increasing attention as a therapeutic strategy, current ferroptosis‐targeting approaches still have notable limitations, including limited tumor selectivity, potential off‐target effects, and insufficient efficacy when used alone [24]. Moreover, in the context of cancer immunotherapy, ferroptosis‐based interventions may not be sufficient to overcome the immunosuppressive tumor microenvironment unless coupled to mechanisms that also restore antitumor immune responsiveness [25]. These challenges underscore the importance of identifying upstream metabolic regulators that can simultaneously modulate ferroptosis sensitivity and immune evasion.

At the same time, STAT3 has emerged as a signaling hub at the intersection of metabolism, ferroptosis regulation, and immune suppression. Previous studies indicate that STAT3 can influence ferroptosis sensitivity through antioxidant and redox‐regulatory pathways, while persistent STAT3 activation has also been associated with impaired CD8^+^ T‐cell function and an immune‐cold tumor microenvironment. This is particularly relevant in TNBC, where the overall benefit of immune checkpoint blockade remains limited, and many tumors exhibit insufficient cytotoxic T‐cell infiltration together with adaptive immunosuppressive programs [26, 27]. Because mitochondrial transporters coordinate metabolic flux, redox homeostasis, and stress adaptation, they are well positioned to influence both ferroptosis susceptibility and antitumor immunity [28, 29]. We therefore hypothesized that the mitochondrial carrier SLC25A13 may act as an upstream immunometabolic regulator that couples mitochondrial metabolism to ferroptosis resistance and immune evasion in TNBC.

It remains unclear whether and how the mitochondrial carrier SLC25A13 couples mitochondrial metabolism to ferroptosis and antitumor immunity. Here, we focus on SLC25A13 and show that it is broadly upregulated across multiple solid tumors and inversely correlated with CD8^+^ cytotoxic T‐cell infiltration. We demonstrate that SLC25A13 directly interacts with STAT3 to enhance complex I‐dependent oxidative phosphorylation while limiting excessive mitochondrial ROS production, thereby promoting sustained STAT3 activation and nuclear accumulation. Nuclear STAT3 transcriptionally upregulates IFI6, maintaining mitochondrial membrane potential, restraining Fe^2^^+^ accumulation and lipid peroxidation, and attenuating CD8^+^ T‐cell effector functions, collectively establishing an SLC25A13–STAT3–IFI6 axis that concomitantly suppresses tumor‐cell ferroptosis and drives immune evasion. Functionally, genetic ablation of SLC25A13 markedly sensitizes tumor cells to CD8^+^ T cell‐induced ferroptotic killing. Furthermore, through high‐throughput small‐molecule screening, we identify HY‐QS02682823 as a selective SLC25A13 inhibitor that promotes SLC25A13 protein degradation, relieves STAT3–IFI6–dependent ferroptosis protection, and robustly sensitizes tumor cells to ferroptosis and CD8^+^ T‐cell cytotoxicity in vitro and in vivo. HY‐QS02682823 also synergizes with anti‐PD‐1 therapy to enhance antitumor efficacy. Together, these findings establish the SLC25A13–STAT3–IFI6 axis as a central node integrating mitochondrial metabolism, ferroptosis control, and immune evasion, and highlight therapeutic targeting of SLC25A13 as a promising strategy to improve cancer immunotherapy.

## 2 Results

### 2.1 SLC25A13 is Highly Expressed in Breast Cancer and is Associated With Immunosuppression and Poor Prognosis

To define the pan‐cancer landscape of SLC25A13 alterations, we systematically analyzed copy‐number changes and somatic mutations across large public cohorts. Pan‐cancer profiling revealed genomic alterations of SLC25A13 in a broad spectrum of solid and hematologic malignancies. These events included amplifications, single‐nucleotide variants, deep deletions, and structural variants. Overall alteration frequencies ranged from ∼2% to 8%. In breast cancer, SLC25A13 mutations were rare, occurring in ∼0.55% of cases (Extended Data Figure S1A).

We next mapped these variants onto the SLC25A13 protein. Mutations were distributed across the full‐length 676‐amino acid sequence. They comprised dispersed missense substitutions as well as truncating events with potential functional impact. Notably, several alterations clustered within or adjacent to the mitochondrial transporter functional domain, and R355*/Q emerged as a relatively recurrent hotspot (Extended Data Figure S1B). Together, these data indicate that SLC25A13 undergoes low‐frequency yet widespread genomic perturbations across cancers. Domain‐enriched mutations further suggest that its mitochondrial transport function may play a conserved and functionally important role in tumorigenesis. Given prior evidence linking SLC25A13 to metabolic rewiring and tumor progression in multiple cancer types—and the lack of a systematic investigation in breast cancer—we nominate SLC25A13 as an underappreciated functional driver in this disease. We therefore focused subsequent analyses on breast cancer to dissect its expression features and immune‐associated mechanisms.

**FIGURE 1:**
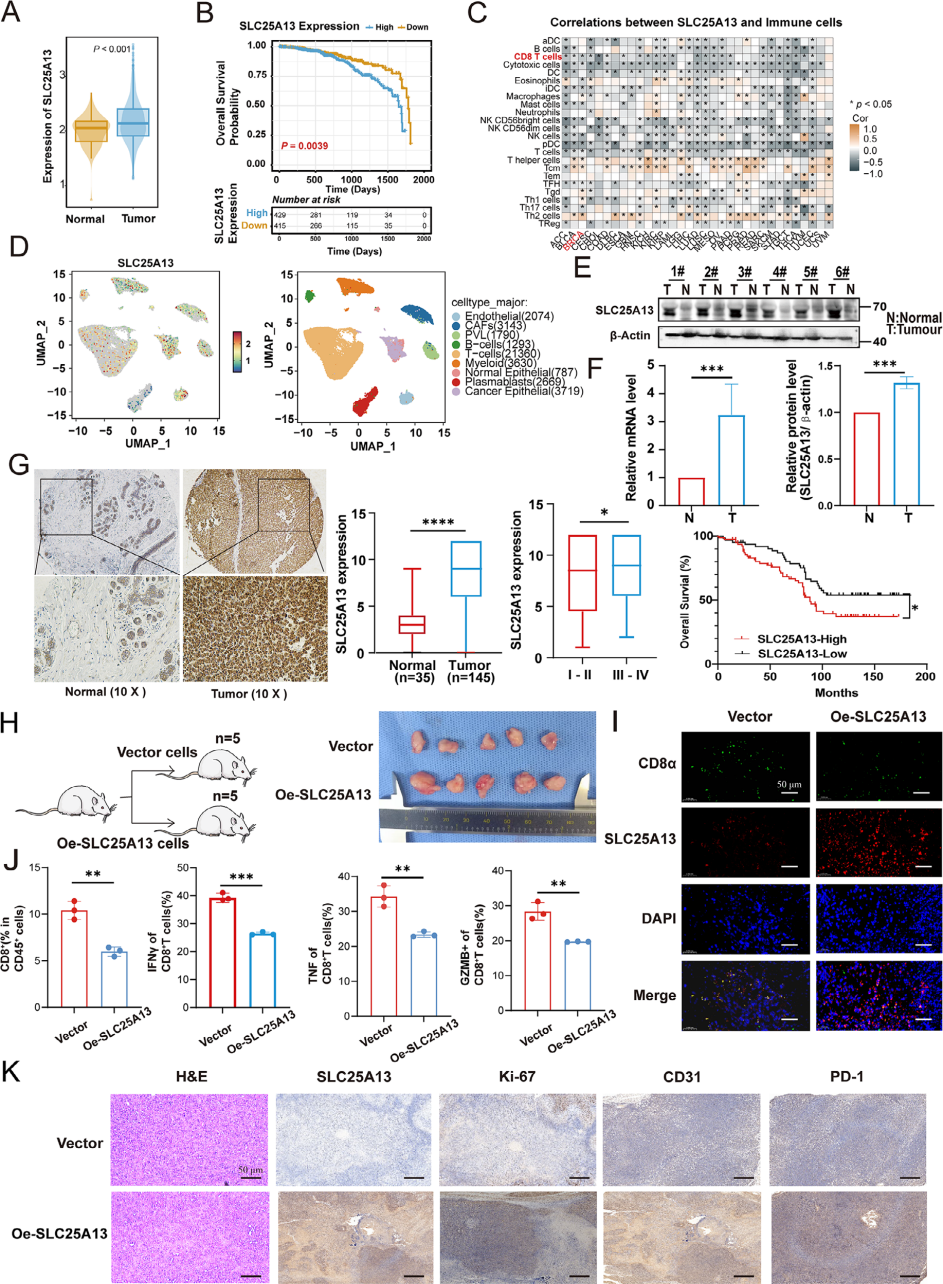
SLC25A13 is upregulated in breast cancer and associates with immunosuppression and poor prognosis. (A) Violin plots comparing SLC25A13 mRNA expression in normal breast tissues versus breast tumors from public cohorts. (B) Kaplan–Meier overall survival curves with risk tables for patients stratified by high versus low SLC25A13 expression. (C) Heat map showing correlations between SLC25A13 expression and inferred immune cell infiltration; the red box highlights significant associations with key immune populations, including CD8^+^ T cells. (D) UMAP projection of SLC25A13 expression across cell types in single‐cell RNA‐seq data; accompanying UMAP clustering of major cellular compartments from the same dataset. (E) Representative WB of SLC25A13 protein levels in paired tumor and adjacent normal tissues from breast cancer patients. (F) Quantification of SLC25A13 mRNA expression and densitometric quantification of SLC25A13 WB in paired tumor and normal tissues. (G) Representative IHC images of SLC25A13 in clinical tissue samples. The three panels on the right show, in order: comparison of SLC25A13 expression scores between normal tissues (*n* = 35) and breast cancer tissues (*n* = 145), comparison of SLC25A13 expression across clinical stages, and a Kaplan–Meier overall survival (OS) curve stratified by high versus low SLC25A13 expression. (H) Schematic of the orthotopic syngeneic tumor model in BALB/c mice using breast cancer cells stably overexpressing SLC25A13 or control cells, with representative endpoint gross tumor images. (I) Representative immunofluorescence images of tumor sections stained for CD8α, SLC25A13, and DAPI. (J) Flow cytometric quantification of the frequency of CD8^+^ T cells among CD45^+^ tumor‐infiltrating leukocytes, and the proportions of IFN‐γ^+^, TNF‐α^+^, and GZMB^+^ cells within the CD8^+^ T cell compartment. (K) Representative H&E staining and IHC images of SLC25A13, Ki‐67, CD31, and PD‐1 in orthotopic tumors (serial sections). Statistical analysis. Two‐group comparisons in (A), (F), (G), and (J) were performed using two‐tailed Student's *t*‐tests; for matched tumor and adjacent normal tissues in (F), a paired two‐tailed Student's *t*‐test was used where appropriate. Survival differences in (B) were analyzed using the log‐rank (Mantel–Cox) test. Correlations in (C) were assessed using two‐sided Spearman's rank correlation. Comparisons among more than two groups were analyzed by one‐way ANOVA with Dunnett's multiple‐comparisons test. Experiments involving two independent variables were analyzed by two‐way ANOVA with Tukey's multiple‐comparisons test. Unless otherwise indicated, data are presented as mean ± SEM. All *p‐*values were two‐sided, and *p < 0.05 was considered statistically significant.

To define the expression pattern of SLC25A13 in breast cancer and its relationship to the tumor immune microenvironment, we integrated public bulk transcriptomic datasets and single‐cell RNA‐seq profiles. Bulk RNA‐seq analyses showed that SLC25A13 mRNA levels were significantly elevated in breast tumors compared with normal breast tissue (Figure 1A). Patients with high SLC25A13 expression exhibited a statistically significant reduction in overall survival (Figure 1B), suggesting an association with adverse prognosis. Immune‐infiltration analyses further linked SLC25A13 to the immune landscape. In particular, SLC25A13 expression negatively correlated with CD8^+^ T cell infiltration (Figure 1C), suggesting a potential role in restraining antitumor immunity.

At single‐cell resolution, we annotated the breast cancer microenvironment using established lineage markers. Marker‐based classification (CD3D, CD4, CD8A, CD68, PECAM1, EPCAM, MS4A1, PDGFRB, among others) resolved major cellular compartments, including CD4^+^ T cells, CD8^+^ T cells, epithelial cells, myeloid cells, endothelial cells, stromal cells, B cells, and proliferating cells (Extended Data Figure S2A–K). We then projected SLC25A13 expression across these populations. SLC25A13 was predominantly enriched in malignant epithelial cells, while remaining minimally expressed in immune subsets, including CD8^+^ T cells (Figure 1D). These data support SLC25A13 as a tumor cell‐intrinsic molecule and argue against confounding by immune cell‐derived expression.

We next validated SLC25A13 upregulation in our in‐house breast cancer cohort. qPCR and WB (Western blot) showed that SLC25A13 mRNA and protein levels were significantly higher in tumors than in matched adjacent normal tissues (Figure 1E). SLC25A13 expression was further increased in patients with advanced‐stage disease. Immunohistochemistry (IHC) corroborated these findings. Tumors displayed markedly stronger SLC25A13 staining than normal mammary glands, and high SLC25A13 expression was associated with reduced overall survival (Figure 1F,G).

To test the function, we generated 4T1 breast cancer cells with stable SLC25A13 overexpression and established an orthotopic mammary fat pad tumor model in immunocompetent BALB/c mice. Compared with control tumors, SLC25A13 overexpression significantly accelerated tumor growth (Figure 1H). It also reduced the frequency of tumor‐infiltrating CD8^+^ T cells and suppressed their effector output, including IFN‐γ, TNF‐α, and GZMB (Figure 1J). Consistently, immunofluorescence revealed markedly decreased CD8α^+^ T cell infiltration in SLC25A13‐high tumors (Figure 1I). IHC further showed that SLC25A13‐overexpressing tumors exhibited increased Ki‐67 and CD31 staining and an elevated number of PD‐1–positive cells (Figure 1K), indicating concomitant enhancement of proliferative activity and an immunosuppressive phenotype.

Together, these data establish that SLC25A13 is predominantly upregulated in malignant epithelial cells in breast cancer. Elevated SLC25A13 tracks with restricted CD8^+^ T cell infiltration, poor clinical outcome, and heightened proliferation and immunosuppression. This multi‐level evidence supports SLC25A13 as a key metabolic regulator of the tumor immune microenvironment.

### 2.2 Biological Functions of SLC25A13

**FIGURE 2:**
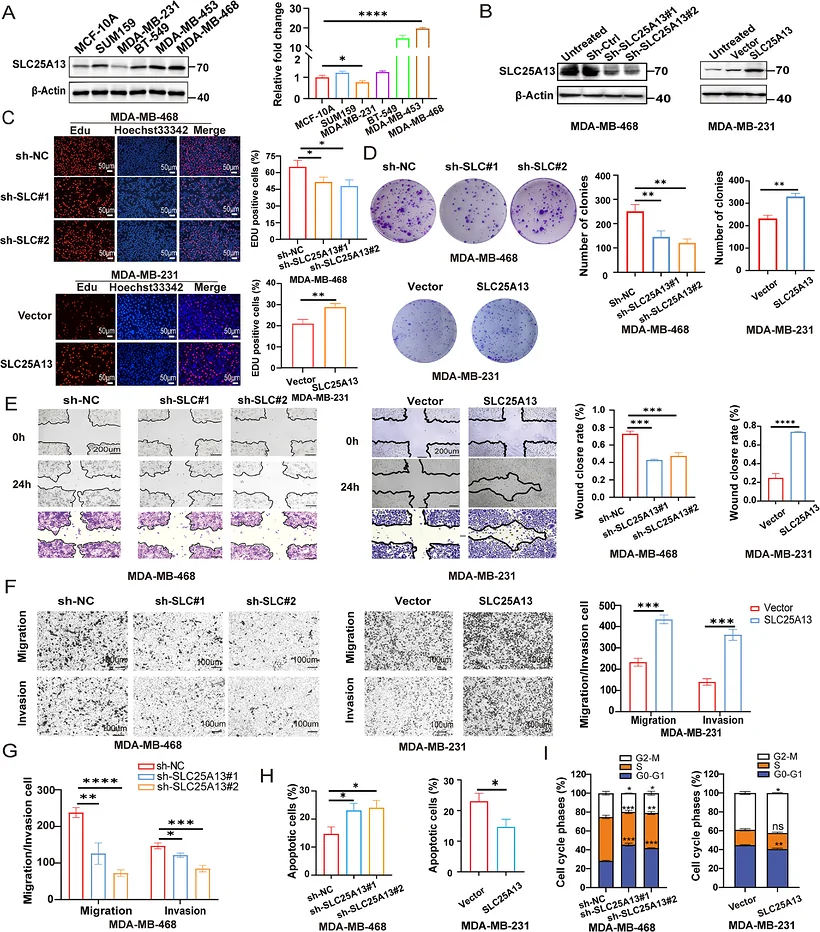
SLC25A13 is highly expressed in breast cancer cells and promotes proliferation, migration, and invasion while suppressing apoptosis. (A) WB analysis and quantification of SLC25A13 protein in the non‐malignant mammary epithelial cell line MCF‐10A and breast cancer cell lines. (B) WB analysis and quantification of SLC25A13 protein levels after genetic manipulation in MDA‐MB‐468 and MDA‐MB‐231 cells. (C) EdU incorporation assays to assess DNA synthesis. (D) Representative colony formation images and quantification showing the effects of SLC25A13 knockdown or overexpression on clonogenic capacity. (E) Wound‐healing assays in MDA‐MB‐468 and MDA‐MB‐231 cells at 0 and 24 h, with quantification of wound closure. (F) Representative images and quantification of transwell migration and invasion assays. (G) Quantification of migrated and invaded cells in MDA‐MB‐468 cells, showing that SLC25A13 knockdown suppresses migration and invasion. (H–I) Flow cytometry (Annexin V/PI) analysis and quantification of apoptosis and cell‐cycle distribution in MDA‐MB‐468 and MDA‐MB‐231 cells with SLC25A13 knockdown or overexpression. Data in (A–I) summarize three independent biological replicates; images in (C), (E), and (F) are representative micrographs. For the bar plot in (A) and for multi‐group comparisons in (D,E), and (G), one‐way ANOVA with Dunnett's multiple‐comparisons test was used. For the bar plot in (B) (knockdown/overexpression efficiency), apoptosis quantification (H), and two‐group comparisons in cell‐cycle analysis (I), two‐tailed unpaired Student's *t*‐tests were used. Data are presented as mean ± SEM. *p < 0.05, **p < 0.01, ***p < 0.001, ****p < 0.0001; ns, not significant.

To investigate the functional role of SLC25A13 in breast cancer progression, we first profiled its expression across a panel of mammary epithelial and breast cancer cell lines. SLC25A13 expression varied substantially among the tested cells, with the highest levels observed in MDA‐MB‐453 and MDA‐MB‐468, modest expression in SUM159 and BT‐549, and relatively low expression in MDA‐MB‐231 (Figure 2A). We therefore established stable SLC25A13‐knockdown cells in MDA‐MB‐468 and SLC25A13‐overexpressing cells in MDA‐MB‐231 for subsequent functional analyses (Figure 2B).

EdU incorporation assays revealed that SLC25A13 depletion significantly reduced DNA synthesis in MDA‐MB‐468 cells, whereas SLC25A13 overexpression increased DNA synthesis in MDA‐MB‐231 cells (Figure 2C). Colony formation assays yielded consistent results. Knockdown of SLC25A13 impaired clonogenic growth, while forced expression of SLC25A13 enhanced colony formation (Figure 2D). We next assessed migratory and invasive phenotypes. SLC25A13 knockdown substantially attenuated wound closure, migration, and invasion of MDA‐MB‐468 cells. Conversely, SLC25A13 overexpression accelerated wound healing and increased migration and invasion in MDA‐MB‐231 cells (Figure 2E–G).

Annexin V/PI flow cytometry further showed that SLC25A13 depletion increased apoptosis in MDA‐MB‐468 cells. In contrast, SLC25A13 overexpression reduced the fraction of apoptotic cells in MDA‐MB‐231 cells (Figure 2H), indicating that SLC25A13 confers a survival advantage. Cell‐cycle profiling was concordant. Silencing SLC25A13 increased the proportion of cells in *G*_0_/*G*_1_ and decreased the S‐phase fraction in MDA‐MB‐468 cells. By contrast, SLC25A13 overexpression in MDA‐MB‐231 cells favored cell‐cycle progression from *G*_0_/*G*_1_ into the proliferative *S*/*G*_2_–M compartments (Figure 2I).

Collectively, these data indicate that SLC25A13 exerts a key oncogenic function in breast cancer. It promotes tumor cell proliferation, migration, and invasion. It suppresses apoptosis and accelerates cell‐cycle progression. These functional findings provide direct experimental support for our public‐dataset analyses and reinforce the view that SLC25A13 may act as a potential driver in breast cancer.

### 2.3 SLC25A13 Suppresses Ferroptosis in Breast Cancer Cells and Maintains Intracellular Iron Homeostasis, Redox Balance, and Mitochondrial Function

**FIGURE 3:**
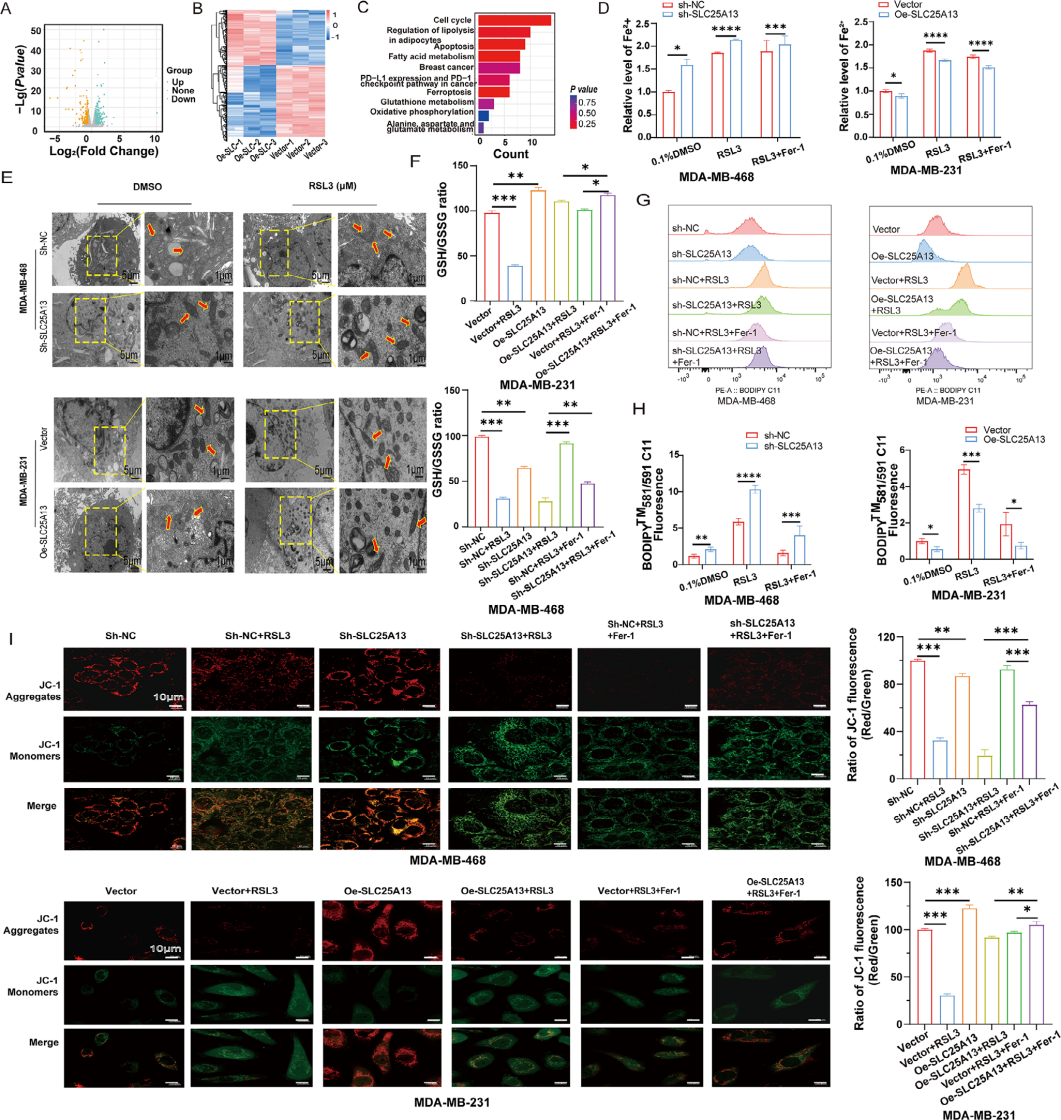
SLC25A13 suppresses ferroptosis in breast cancer cells and preserves intracellular iron homeostasis, redox balance, and mitochondrial function. (A) Volcano plot of differentially expressed genes. (B) Heatmap of differentially expressed genes (DEGs), showing representative changes in gene expression profiles between the SLC25A13 overexpression group and the control group. (C) Pathway enrichment analysis of the differentially expressed genes. (D) Relative intracellular Fe^2^^+^ levels measured by a fluorescent probe in MDA‐MB‐468 and MDA‐MB‐231 cells treated with DMSO, RSL3, or RSL3 + ferrostatin‐1 (Fer‐1). (E) Transmission electron microscopy (TEM) images showing ultrastructural changes in MDA‐MB‐468 and MDA‐MB‐231 cells treated with 0.1% DMSO or RSL3 (8 µm, 24 h). Red arrows indicate ferroptosis‐associated mitochondrial abnormalities. Scale bars, 5 µm (left panels) and 1 µm (right panels). (F) GSH/GSSG ratios in MDA‐MB‐231 and MDA‐MB‐468 cells under the indicated conditions, reflecting SLC25A13‐dependent control of intracellular redox homeostasis. (G,H) Lipid peroxidation assessed by BODIPY 581/591 C11 staining in MDA‐MB‐468 and MDA‐MB‐231 cells treated with 0.1% DMSO, RSL3 (8 µm, 24 h), or RSL3 + Fer‐1 (2 µm, 24 h). Representative flow‐cytometry histograms are shown in (G), and the corresponding quantification of fluorescence intensity is shown in (H). (I) Mitochondrial membrane potential measured by JC‐1 staining in MDA‐MB‐468 and MDA‐MB‐231 cells under the indicated conditions. Statistics. Bar plots are presented as mean ± s.d. unless otherwise indicated. Two‐group comparisons were performed using two‐tailed Student's *t*‐tests. Multi‐group comparisons were analyzed by one‐way ANOVA with Dunnett's multiple‐comparisons test. *p < 0.05, **p < 0.01, ***p < 0.001, ****p < 0.0001; ns, not significant.

To probe how SLC25A13 contributes to malignant progression, we first performed transcriptomic profiling of BALB/c syngeneic tumors. KEGG enrichment analysis indicated that SLC25A13‐associated differentially expressed genes were linked not only to the cell cycle, lipid metabolism, and the PD‐1/PD‐L1 pathway, but also to glutathione metabolism, oxidative phosphorylation, and ferroptosis (Figure 3A–C). These signatures suggested that SLC25A13 may shape tumor cell fate by modulating ferroptosis.

We next characterized the dose response to the ferroptosis inducer RSL3 in MDA‐MB‐468 and MDA‐MB‐231 cells. As RSL3 concentrations increased from 0 to 8 µm, GPX4 protein levels declined in a dose‐dependent manner. This was accompanied by downregulation of xCT and upregulation of ACSL4, consistent with engagement of the canonical ferroptosis program. These changes were most pronounced at 8 µm. Notably, SLC25A13 protein levels also decreased at the same dose (Extended Data Figure S3A), implicating SLC25A13 in the cellular response to RSL3‐driven ferroptotic stress. We then directly tested the functional impact of SLC25A13 using genetic perturbation. Silencing SLC25A13 markedly increased intracellular malondialdehyde (MDA) levels in both MDA‐MB‐468 and MDA‐MB‐231 cells. Co‐treatment with RSL3 further amplified MDA accumulation (Extended Data Figure S3B,C), indicating exacerbated lipid peroxidation. Consistent with ferroptotic ultrastructure, transmission electron microscopy revealed shrunken mitochondria with reduced cristae and increased membrane density in sh‐SLC25A13 cells. These mitochondrial lesions became even more pronounced when SLC25A13 knockdown was combined with RSL3 (Figure 3E). JC‐1 staining provided complementary functional readouts. SLC25A13 depletion reduced mitochondrial membrane potential and potentiated the loss of membrane potential induced by RSL3. Ferrostatin‐1 (Fer‐1) partially rescued this defect. By contrast, SLC25A13 overexpression maintained a higher membrane potential under ferroptotic stress (Figure 3I).

Functionally, RSL3 dose–response curves further established SLC25A13 as a negative regulator of ferroptosis. Building on these results, we introduced a second ferroptosis inhibitor with a distinct chemical scaffold, liproxstatin‐1. Stable SLC25A13 knockdown markedly increased RSL3 cytotoxicity in MDA‐MB‐468 cells. This sensitization was abolished by liproxstatin‐1. In contrast, neither the pan‐caspase inhibitor Z‐VAD‐FMK nor the necroptosis inhibitor necrosulfonamide restored cell viability. Conversely, SLC25A13 overexpression in MDA‐MB‐231 cells attenuated RSL3‐induced cell death. Co‐treatment with liproxstatin‐1 rescued viability to near‐control levels, whereas Z‐VAD‐FMK or necrosulfonamide had little effect (Extended Data Figure S3D,E). These orthogonal pharmacologic validations indicate that the pro‐survival activity of SLC25A13 is primarily mediated through suppression of ferroptosis, rather than apoptosis or necroptosis.

Upon RSL3 treatment, intracellular Fe^2^^+^ levels rose sharply. SLC25A13 depletion further amplified Fe^2^^+^ accumulation, whereas SLC25A13 overexpression blunted this response. In both genetic contexts, ferrostatin‐1 effectively reversed these changes (Figure 3D). RSL3 also disrupted intracellular redox homeostasis, as reflected by a reduced GSH/GSSG ratio. This defect was aggravated by SLC25A13 depletion but alleviated by SLC25A13 overexpression, and was partially reversed by ferrostatin‐1 (Figure 3F). In parallel, representative flow‐cytometry histograms and quantitative analysis of BODIPY 581/591 C11 oxidation showed that RSL3 robustly induced lipid ROS in both models. SLC25A13 silencing further intensified lipid peroxidation, whereas SLC25A13 overexpression attenuated this response. In both settings, co‐treatment with ferrostatin‐1 markedly reduced lipid ROS accumulation (Figure 3G,H). Collectively, multiple independent and concordant lines of evidence converge on a single conclusion. SLC25A13 functions as a central brake on ferroptosis in breast cancer cells. It preserves intracellular iron homeostasis, sustains antioxidant capacity, and maintains mitochondrial integrity. By doing so, SLC25A13 confers robust ferroptosis resistance and provides tumor cells with a clear survival advantage.

### 2.4 IFI6 is Highly Expressed in Breast Cancer and Suppresses Ferroptosis Through Multi‐Layered Mechanisms

**FIGURE 4:**
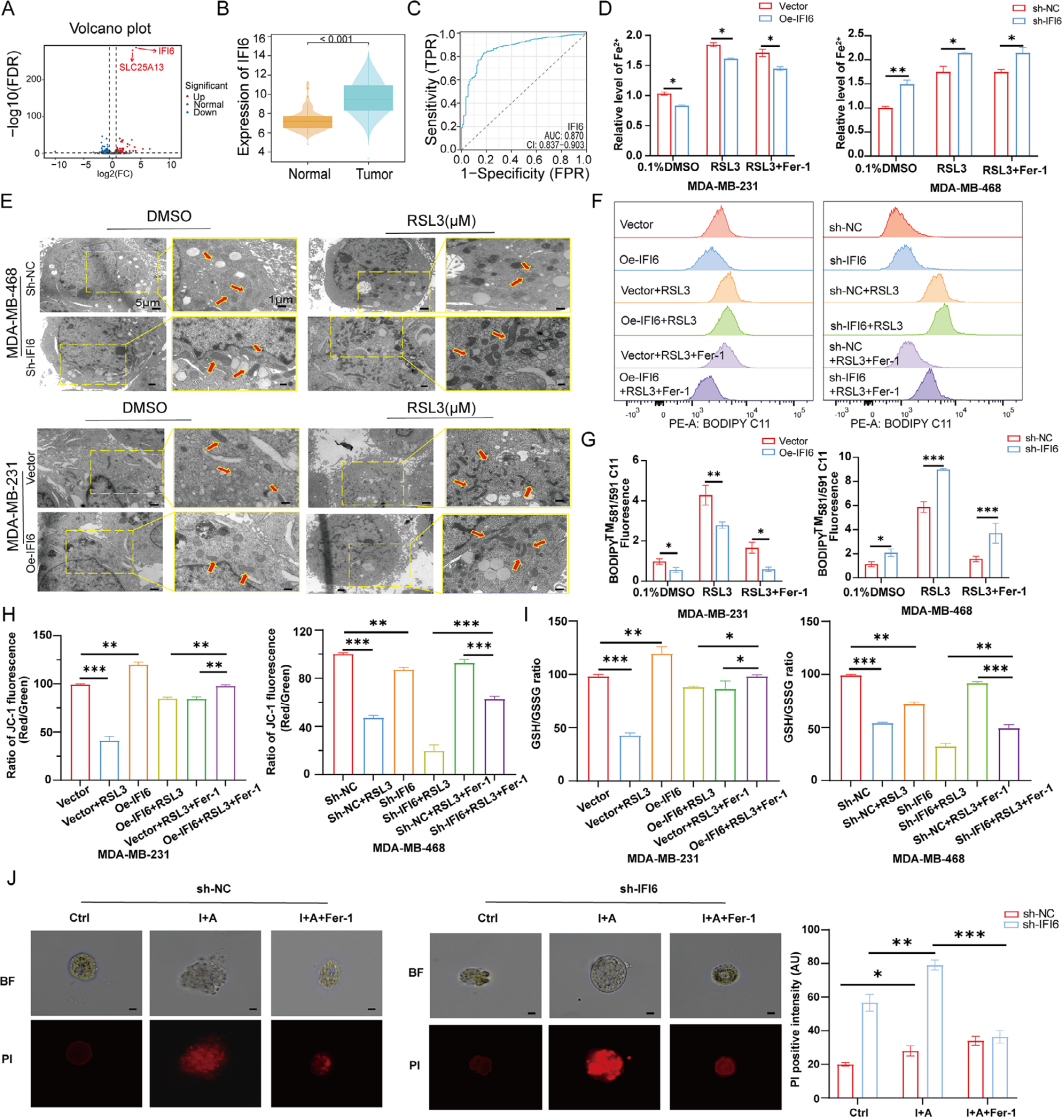
IFI6 is upregulated in breast cancer and suppresses ferroptosis through multi‐layered mechanisms. (A) Volcano plot of RNA‐seq differential expression analysis comparing control cells and SLC25A13‐overexpressing breast cancer cells. (B) Violin plots of IFI6 mRNA expression in normal breast tissues and breast tumors in the TCGA cohort (N = 113, T = 1113). (C) Receiver operating characteristic (ROC) curve evaluating the ability of IFI6 to discriminate tumor from normal samples, with AUC and confidence interval indicated. (D) Relative intracellular Fe^2^^+^ levels in MDA‐MB‐231 cells and MDA‐MB‐468 cells under the indicated conditions. (E) TEM images showing ultrastructural changes in MDA‐MB‐468 cells and MDA‐MB‐231 cells treated with DMSO or RSL3. Dashed boxes indicate regions shown at higher magnification, and red arrows highlight representative mitochondria with ferroptosis‐associated morphological features. [Scale bars, 5 µm (overview images) and 1 µm (magnified images).] (F,G) Lipid peroxidation assessed by BODIPY 581/591 C11 staining in MDA‐MB‐231 and MDA‐MB‐468 cells under the indicated conditions. Representative flow‐cytometry histograms (F) and the corresponding quantification of BODIPY 581/591 C11 fluorescence (G) are shown. (H) Mitochondrial membrane potential assessed by JC‐1 staining in MDA‐MB‐231 and MDA‐MB‐468 cells under the indicated conditions. (I) GSH/GSSG ratios in MDA‐MB‐231 and MDA‐MB‐468 cells under the indicated conditions, reflecting intracellular redox homeostasis. (J) Bright‐field (BF) and propidium iodide (PI) images of patient‐derived breast cancer organoids transduced with sh‐NC or sh‐IFI6 and treated with control (Ctrl), ferroptosis induction (IKE + arachidonic acid; I(20 ng/mL)+AA(20 µm)), or I+A + ferrostatin‐1 (Fer‐1). Scale bar, 50 µm. (*n* = 3) Quantification in (B,D), (G–I) is derived from three independent biological replicates and is presented as mean ± SD. The comparison in (B) was performed using a two‐tailed unpaired Student's *t*‐test. For (D), (G–I), differences among treatment groups were evaluated by one‐way ANOVA with Dunnett's multiple‐comparisons test. Panels (E) and (J) show representative TEM images and organoid PI staining from three independent experiments. *p < 0.05, **p < 0.01, *** *p* < 0.001.

To identify downstream effectors through which SLC25A13 controls ferroptosis, we prioritized candidates by integrating our transcriptomic dataset with differential expression filtering. Volcano plot analysis highlighted IFI6 as the most prominently upregulated gene among those significantly altered upon SLC25A13 perturbation, nominating it as a leading candidate (Figure 4A). Prior studies in solid tumors, including hepatocellular carcinoma and gastric cancer, have linked IFI6 to ferroptosis control and oxidative stress responses [19, 20]. IFI6 has also been implicated in tumor immune evasion and ferroptosis resistance, raising the possibility that it functions as a key downstream mediator of SLC25A13‐driven ferroptosis regulation.

Consistent with this notion, analysis of the TCGA‐BRCA cohort showed that IFI6 mRNA was markedly elevated in breast tumors relative to normal breast tissues (Figure 4B). Receiver operating characteristic (ROC) analysis further demonstrated a strong discriminatory capacity of IFI6 for distinguishing tumor from normal samples (Figure 4C). Moreover, correlation analysis in the independent TNBC dataset GSE27447 revealed a modest but significant positive association between SLC25A13 and IFI6 expression (Extended Data Figure S4A), further supporting the clinical relevance of the SLC25A13–IFI6 axis in TNBC. Before examining ferroptosis‐related phenotypes, we asked whether IFI6 also contributes to canonical malignant behaviors in breast cancer cells. In MDA‐MB‐231 cells, ectopic IFI6 expression enhanced colony formation and proliferation, whereas IFI6 silencing in MDA‐MB‐468 cells reduced both phenotypes. Likewise, wound‐healing and transwell assays showed that IFI6 promoted migration and invasion in MDA‐MB‐231 cells, while IFI6 knockdown suppressed these properties in MDA‐MB‐468 cells (Extended Data Figure S5A–K). These data indicate that IFI6 not only correlates with adverse clinical features but also directly supports tumor‐promoting phenotypes in breast cancer cells.

We next investigated whether IFI6 modulates ferroptosis‐associated metabolic and structural phenotypes in breast cancer cells. In MDA‐MB‐231 cells, ectopic IFI6 expression reduced both basal and RSL3‐induced Fe^2^^+^ accumulation, whereas IFI6 silencing in MDA‐MB‐468 cells increased intracellular Fe^2^^+^ levels under the same conditions (Figure 4D). These findings suggested that IFI6 contributes to iron homeostasis under ferroptotic stress. Ultrastructural analysis by transmission electron microscopy further substantiated this role. RSL3 induced classical ferroptosis‐associated mitochondrial alterations, including mitochondrial shrinkage, increased membrane density, and reduction or disappearance of cristae. These abnormalities were aggravated by IFI6 knockdown in MDA‐MB‐468 cells but were alleviated by IFI6 overexpression in MDA‐MB‐231 cells (Figure 4E), indicating that IFI6 preserves mitochondrial integrity during ferroptotic challenge.

Because lipid peroxidation is a defining feature of ferroptosis, we next examined BODIPY 581/591 C11 oxidation. Representative flow‐cytometric histograms and quantitative analysis showed that RSL3 robustly increased lipid ROS in both models, whereas IFI6 overexpression blunted this increase and IFI6 depletion further amplified it (Figure 4F,G). Importantly, co‐treatment with ferrostatin‐1 (Fer‐1) markedly attenuated lipid peroxidation in both genetic contexts, supporting that the observed changes were ferroptosis‐related. Together with the Fe^2^^+^ data, these results indicate that IFI6 restrains the core biochemical events that drive ferroptotic cell death.

We then assessed whether IFI6 also influences mitochondrial function and intracellular redox buffering under ferroptotic stress. JC‐1 staining revealed that RSL3 induced a pronounced loss of mitochondrial membrane potential, as reflected by a decreased red/green fluorescence ratio. This defect was exacerbated by IFI6 knockdown but mitigated by IFI6 overexpression, while Fer‐1 partially restored membrane potential in both settings (Figure 4H). In parallel, measurement of the GSH/GSSG ratio showed that RSL3 impaired intracellular reducing capacity, an effect that became more severe upon IFI6 depletion and was alleviated by enforced IFI6 expression (Figure 4I). These data indicate that IFI6 supports both mitochondrial fitness and redox homeostasis, thereby raising the cellular threshold for ferroptosis execution.

To extend these observations beyond conventional monolayer cultures, we evaluated ferroptosis sensitivity in patient‐derived breast cancer organoids. Ferroptosis was induced using IKE plus arachidonic acid (I+A). Under these conditions, sh‐IFI6 organoids displayed more severe structural collapse and markedly increased propidium iodide (PI) positivity compared with control organoids, whereas co‐treatment with Fer‐1 partially restored organoid integrity and reduced PI‐positive cell death (Figure 4J). Thus, the ferroptosis‐protective function of IFI6 is retained in a three‐dimensional patient‐derived model.

Collectively, these data identify IFI6 as a clinically relevant and functionally important downstream effector of the SLC25A13 pathway in breast cancer. IFI6 is upregulated in tumors, preserves mitochondrial structure and membrane potential, limits Fe^2^^+^ accumulation and lipid peroxidation, and sustains intracellular redox homeostasis under ferroptotic stress. By protecting both 2D breast cancer cells and 3D patient‐derived organoids from ferroptosis, IFI6 emerges as a key executor through which SLC25A13 reinforces ferroptosis resistance.

### 2.5 STAT3 Links SLC25A13 to IFI6 and Directly Activates the IFI6 Promoter

**FIGURE 5:**
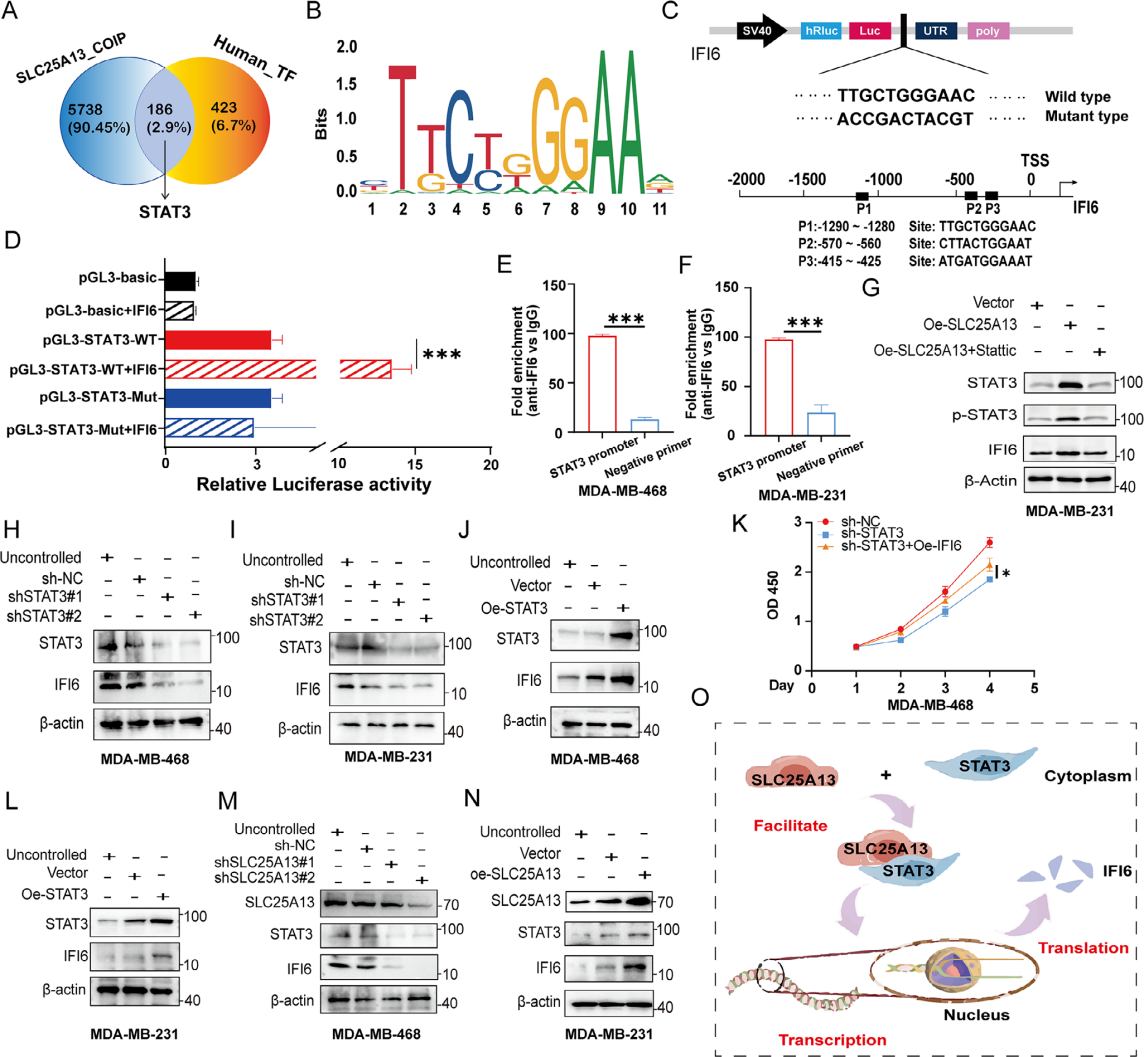
STAT3 is a key transcription factor linking SLC25A13 to IFI6 and directly activates the IFI6 promoter. (A) Venn diagram showing the overlap between proteins identified by SLC25A13 co‐immunoprecipitation (SLC25A13_COIP) and the curated human transcription factor set. (B) Predicted STAT3‐binding motif. (C) Schematic of IFI6 promoter‐driven luciferase reporter constructs. (D) Dual‐luciferase reporter assays demonstrating STAT3‐dependent activation of the IFI6 promoter. (E,F) ChIP–qPCR analysis of STAT3 occupancy at the IFI6 promoter in MDA‐MB‐468 (E) and MDA‐MB‐231 (F) cells. Enrichment is shown as fold change for anti‐STAT3 relative to IgG, comparing IFI6 promoter‐specific primers to negative‐control primers. (G) WB analysis in MDA‐MB‐231 cells showing that Stattic in the SLC25A13‐overexpressing background reduces STAT3, p‐STAT3, and IFI6 expression. (H–I) WB analysis showing that STAT3 knockdown decreases IFI6 protein levels in MDA‐MB‐468 (H) and MDA‐MB‐231 (I) cells. (J,L) WB analysis showing that STAT3 overexpression increases IFI6 protein levels in MDA‐MB‐468 (J) and MDA‐MB‐231 (L) cells. (K) Cell proliferation assay showing that STAT3 knockdown suppresses the growth of MDA‐MB‐468 cells, whereas IFI6 re‐expression partially rescues this effect. (M,N) WB analysis showing that SLC25A13 silencing reduces, whereas SLC25A13 overexpression increases, STAT3 and IFI6 protein levels in MDA‐MB‐468 (M) and MDA‐MB‐231 (N) cells. (O) Schematic model illustrating the proposed SLC25A13–STAT3–IFI6 signaling axis. Statistics. Data are presented as mean ± SD where applicable. Quantitative data in (D), (E,F), and (K) were obtained from three independent experiments. Statistical analyses were performed using two‐tailed unpaired Student's *t*‐test or one‐way ANOVA with appropriate multiple‐comparisons testing, as indicated. *P < 0.05, **P < 0.01, ***P < 0.001.

To define the transcriptional mechanism linking SLC25A13 to IFI6, we first intersected proteins identified by SLC25A13 co‐immunoprecipitation with human transcription factor databases. This analysis yielded 186 overlapping candidates, among which STAT3 emerged as a prominent node (Figure 5A). Motif analysis further showed that STAT3 recognizes the canonical TT(C/G)TGGAA‐like motif (Figure 5B). Consistent with this prediction, sequence inspection of the IFI6 promoter identified three putative STAT3‐binding elements (P1–P3) within the −2000–0 bp region upstream of the transcription start site (Figure 5C).

We next tested whether STAT3 directly activates IFI6 transcription. Dual‐luciferase reporter assays showed that STAT3 robustly increased the activity of the wild‐type IFI6 promoter, whereas mutation of the core P1–P3 motifs markedly blunted this induction (Figure 5D). ChIP–qPCR analysis in both MDA‐MB‐468 and MDA‐MB‐231 cells further demonstrated strong enrichment of STAT3 at the IFI6 promoter, with minimal signal at the negative‐control region (Figure 5E,F). These results indicate that STAT3 directly binds to and transcriptionally activates the IFI6 promoter in breast cancer cells.

To further determine whether SLC25A13‐induced IFI6 upregulation depends on STAT3 activity, we treated SLC25A13‐overexpressing MDA‐MB‐231 cells with the pharmacological STAT3 inhibitor Stattic. Stattic markedly reduced STAT3 and p‐STAT3 levels and attenuated IFI6 upregulation in the SLC25A13‐overexpressing background (Figure 5G), supporting that the induction of IFI6 by SLC25A13 is dependent on STAT3 signaling. Consistently, direct STAT3 depletion in MDA‐MB‐468 and MDA‐MB‐231 cells markedly reduced IFI6 protein abundance (Figure 5H,I), whereas STAT3 overexpression increased IFI6 expression in both cell lines (Figure 5J,L).

Functionally, STAT3 knockdown suppressed cell proliferation, and IFI6 re‐expression partially rescued this defect in MDA‐MB‐468 cells (Figure 5K), supporting IFI6 as an important downstream effector of STAT3. Importantly, perturbation of SLC25A13 produced concordant changes in this pathway: SLC25A13 silencing reduced STAT3 and IFI6 protein levels, whereas SLC25A13 overexpression increased both proteins (Figure 5M,N). Together, these data establish an SLC25A13–STAT3–IFI6 regulatory axis in which SLC25A13 promotes STAT3 activation and, through STAT3‐dependent transcription, drives IFI6 expression (Figure 5O).

### 2.6 SLC25A13 Stabilizes STAT3 Through Direct Interaction

**FIGURE 6:**
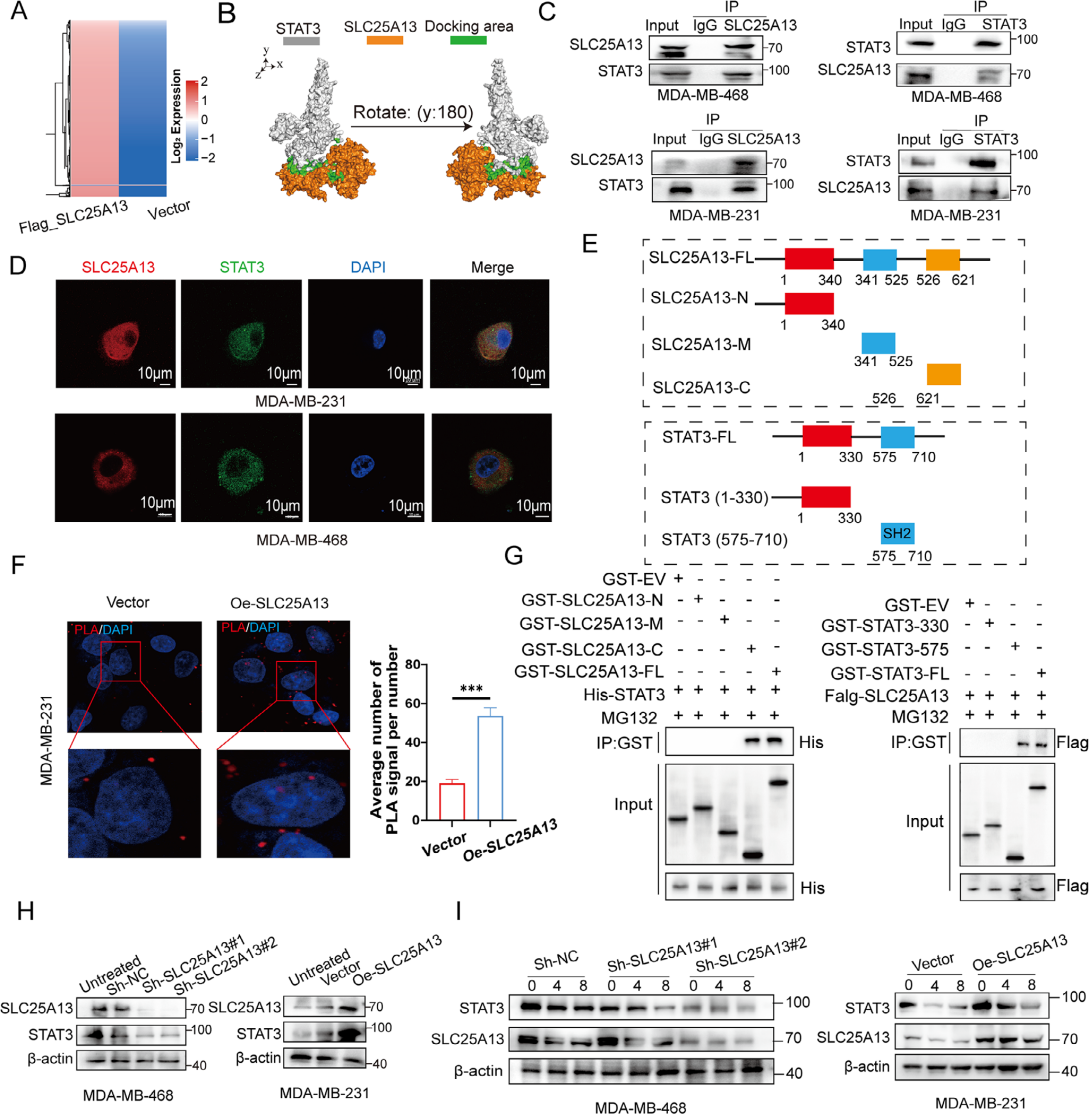
SLC25A13 stabilizes STAT3 through direct interaction. (A) Integrative profiling of MDA‐MB‐231 cells transfected with Vector or Flag‐SLC25A13. (B) Overall structural model of the SLC25A13–STAT3 complex based on molecular docking, showing the predicted docking interface. (C) Endogenous co‐immunoprecipitation (co‐IP) confirming the interaction between SLC25A13 and STAT3 in MDA‐MB‐468 and MDA‐MB‐231 cells. (D) Confocal immunofluorescence images showing intracellular colocalization of SLC25A13 and STAT3 in MDA‐MB‐231 and MDA‐MB‐468 cells. Scale bar, 10 µm. (E) Schematic illustration of SLC25A13 and STAT3 truncation constructs used for interaction mapping. (F) PLA in Vector and Oe‐SLC25A13 MDA‐MB‐231 cells. Representative PLA images are shown on the left, and quantification of the average PLA signal number per cell is shown on the right. Scale bar, 10 µm. (G) GST pull‐down assays showing that the interaction between SLC25A13 and STAT3 involves the C‐terminal region of SLC25A13 and the SH2‐containing region of STAT3. (H) WB analysis of SLC25A13 and STAT3 protein levels following SLC25A13 knockdown in MDA‐MB‐468 cells or SLC25A13 overexpression in MDA‐MB‐231 cells. (I) Cycloheximide (CHX) chase assays in MDA‐MB‐468 and MDA‐MB‐231 cells showing that SLC25A13 knockdown shortens, whereas SLC25A13 overexpression prolongs, STAT3 protein stability. Replication and statistics. Quantification in (F) is presented as mean ± SD from three independent experiments (*n* = 3) and was analyzed by a two‐tailed unpaired Student's *t*‐test. Panels (A–E) and (G–I) show representative images, WB, or schematics. ns, not significant. ***P < 0.001.

To determine whether SLC25A13 regulates STAT3 via a direct physical interaction, we first performed immunoprecipitation followed by mass spectrometry in MDA‐MB‐231 cells. STAT3 emerged as a candidate SLC25A13‐interacting protein (Figure 6A). Molecular docking further predicted a stable association between SLC25A13 and STAT3, with the putative docking interface located within the SLC25A13 structure (Figure 6B).

We next validated this association experimentally. Endogenous co‐immunoprecipitation confirmed that SLC25A13 and STAT3 interact in both MDA‐MB‐468 and MDA‐MB‐231 cells (Figure 6C). Confocal immunofluorescence further showed intracellular colocalization of the two proteins (Figure 6D). To strengthen the evidence beyond conventional colocalization, we performed a proximity ligation assay (PLA) in MDA‐MB‐231 cells and found that SLC25A13 overexpression markedly increased the number of intracellular PLA puncta, supporting enhanced in situ proximity between SLC25A13 and STAT3 (Figure 6F).

To map the interaction domains, we generated truncation constructs of both proteins (Figure 6E) and performed GST pull‐down assays. These analyses showed that full‐length SLC25A13 and its C‐terminal fragment associated with STAT3, whereas reciprocal mapping indicated that the interaction involves the SH2‐containing C‐terminal region of STAT3 (Figure 6G). These data suggest that the association between SLC25A13 and STAT3 involves the SH2‐containing region of STAT3.

We then examined whether SLC25A13 regulates STAT3 expression at the transcriptional or post‐transcriptional level. qPCR analysis showed that STAT3 mRNA levels were not significantly altered by either SLC25A13 knockdown in MDA‐MB‐468 cells or SLC25A13 overexpression in MDA‐MB‐231 cells (Extended Data Figure S4B), arguing against transcriptional regulation. In contrast, WB revealed that STAT3 protein abundance decreased after SLC25A13 silencing and increased upon SLC25A13 overexpression (Figure 6H). Consistently, cycloheximide chase assays showed that SLC25A13 knockdown accelerated STAT3 protein decay, whereas SLC25A13 overexpression prolonged STAT3 stability (Figure 6I).

Together, these results indicate that SLC25A13 physically associates with STAT3 and stabilizes STAT3 protein in a post‐transcriptional manner. This interaction provides an upstream molecular basis for the SLC25A13–STAT3–IFI6 axis.

### 2.7 SLC25A13 Enhances Complex I‐Dependent Oxidative Phosphorylation to Restrain Mitochondrial ROS and Promote STAT3 Nuclear Translocation

**FIGURE 7:**
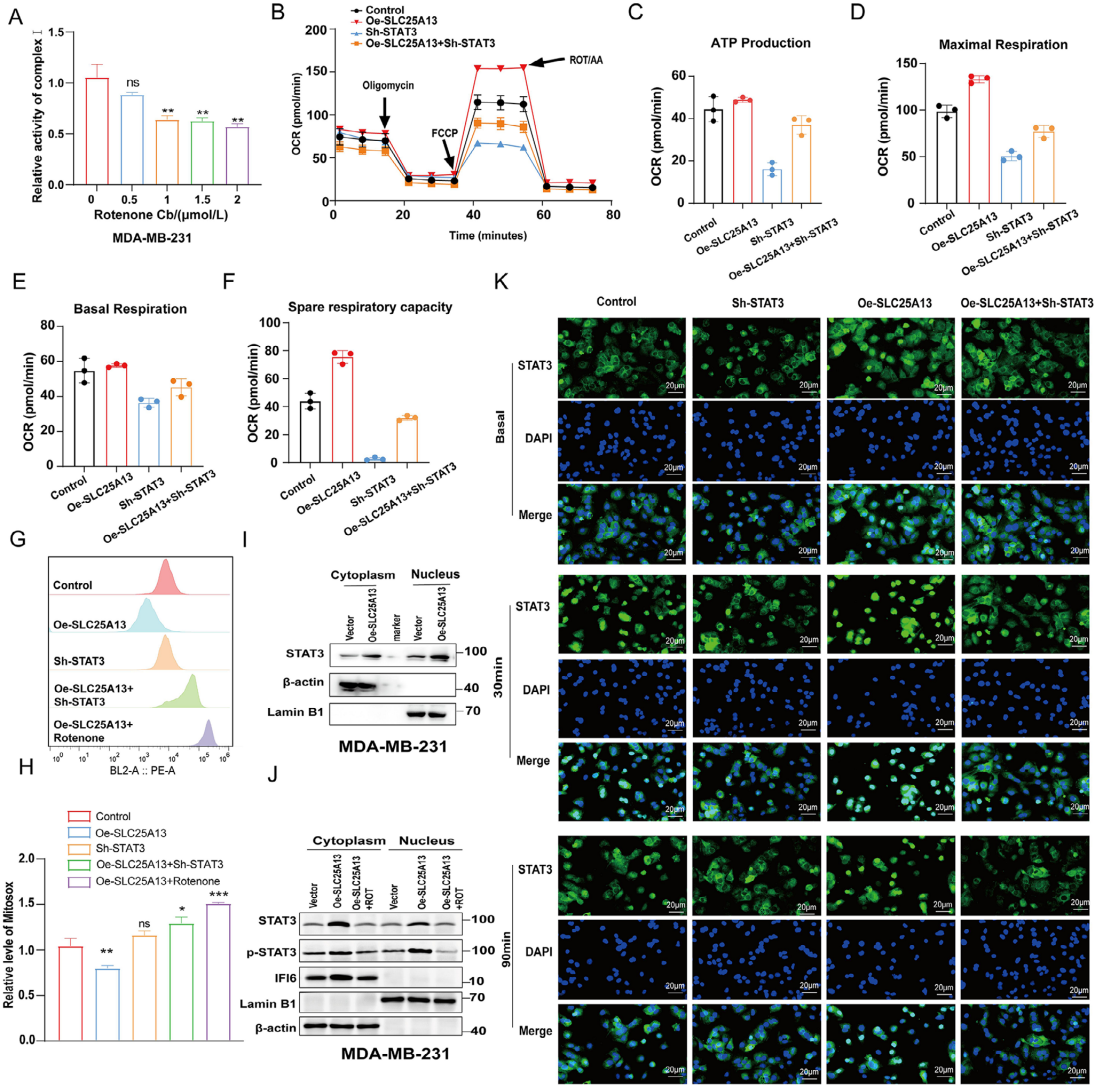
SLC25A13 enhances complex I‐dependent oxidative phosphorylation to restrain mitochondrial ROS and promote STAT3 nuclear translocation. (A) Relative complex I enzymatic activity in MDA‐MB‐231 cells treated with increasing concentrations of rotenone for 2 h, used to define an effective inhibitory dose. (B) Seahorse OCR traces of control, Oe‐SLC25A13, sh‐STAT3, and Oe‐SLC25A13 + sh‐STAT3 cells after sequential injection of oligomycin, FCCP, and rotenone/antimycin A. (C–F) Quantification of OCR‐derived parameters from (B), including ATP‐linked respiration (C), maximal respiration (D), basal respiration (E), and spare respiratory capacity (F). (G,H) Flow cytometric measurement and quantification of mitochondrial ROS using MitoSOX in the indicated groups: control, Oe‐SLC25A13, sh‐STAT3, Oe‐SLC25A13 + sh‐STAT3, and Oe‐SLC25A13 + rotenone. (I) WB analysis of cytoplasmic and nuclear fractions showing the subcellular distribution of STAT3 in Vector and Oe‐SLC25A13 cells. (J) Subcellular fractionation WB in Vector, Oe‐SLC25A13, and Oe‐SLC25A13 + rotenone cells. (K) Time‐course immunofluorescence analysis of STAT3 localization in control, sh‐STAT3, Oe‐SLC25A13, and Oe‐SLC25A13 + sh‐STAT3 cells under basal, 30 min, and 90 min conditions. Scale bars, 20 µm. Statistics. Data are presented as mean ± SD from three independent experiments unless otherwise indicated. Statistical analyses were performed using one‐way ANOVA with appropriate multiple‐comparisons testing or two‐tailed unpaired Student's *t*‐test, as appropriate. *P < 0.05, **P < 0.01, ***P < 0.001; ns, not significant.

To mechanistically link mitochondrial metabolism to STAT3 activation and ferroptosis suppression, we next asked whether SLC25A13 regulates mitochondrial ROS through complex I, thereby facilitating STAT3 nuclear entry [30, 31, 32]. Building on the SLC25A13–STAT3–IFI6 transcriptional axis defined above, we focused on a model in which SLC25A13 sustains complex I–driven oxidative phosphorylation (OXPHOS) and prevents excessive mitochondrial ROS accumulation. We first performed a titration of the complex I–specific inhibitor rotenone in MDA‐MB‐231 cells. Complex I enzymatic activity was only modestly affected at 0.5 µm rotenone, but was significantly reduced at concentrations of 1 µm or above. We therefore selected 1 µM rotenone as the lowest dose that achieved effective complex I inhibition for subsequent rescue experiments (Figure 7A). We then assessed mitochondrial respiration using Seahorse mitochondrial stress tests. Compared with controls, SLC25A13 overexpression significantly increased basal respiration, maximal respiratory capacity, ATP production, and spare respiratory capacity. In contrast, STAT3 knockdown broadly reduced these parameters. Importantly, co‐silencing STAT3 in the SLC25A13‐overexpressing background largely abolished the respiration‐enhancing effects of SLC25A13 (Figure 7B–F). These findings suggest that SLC25A13 enhances complex I‐linked OXPHOS in a manner that is at least partly dependent on STAT3.

We next quantified mitochondrial ROS using MitoSOX. SLC25A13 overexpression markedly reduced mitochondrial ROS levels. This effect was reversed by STAT3 knockdown. It was also reversed by pharmacologic inhibition of complex I with rotenone in SLC25A13‐overexpressing cells, which restored ROS to near‐control levels (Figure 7G,H). These data support a model in which SLC25A13 sustains a complex I‐dependent respiratory state that limits excessive mitochondrial ROS accumulation.

We then asked whether this redox state influences STAT3 nuclear translocation. Subcellular fractionation showed that SLC25A13 overexpression increased nuclear STAT3 relative to the cytoplasmic fraction (Figure 7I). Consistently, when mitochondrial ROS was restored by rotenone, the nuclear accumulation of STAT3 and p‐STAT3 was attenuated, and IFI6 expression was concomitantly reduced (Figure 7J). Time‐course immunofluorescence further demonstrated enhanced nuclear redistribution of STAT3 in SLC25A13‐overexpressing cells, whereas STAT3 knockdown markedly weakened this response (Figure 7K). Together, these findings support a model in which SLC25A13 promotes STAT3 nuclear accumulation by sustaining complex I‐linked oxidative phosphorylation and restraining mitochondrial ROS, thereby creating a redox environment permissive for STAT3‐dependent IFI6 induction.

### 2.8 SLC25A13 Attenuates CD8^+^ T Cell‐Mediated Tumor Killing and Promotes Immune Evasion

**FIGURE 8:**
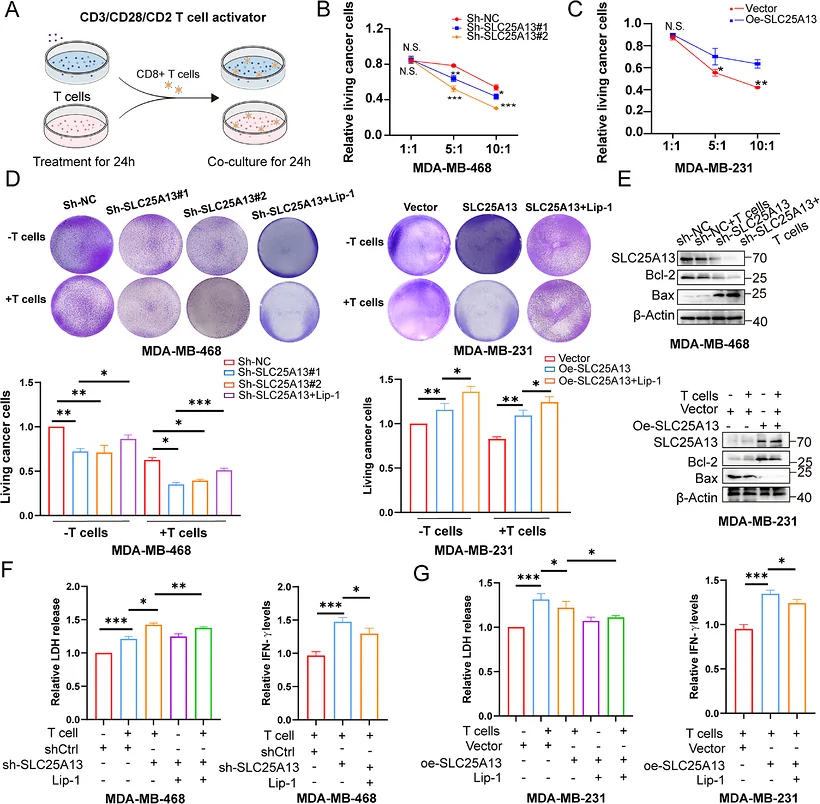
SLC25A13 attenuates CD8^+^ T cell‐mediated tumor killing and promotes immune evasion. (A) Schematic of the in vitro co‐culture assay. (B,C) Relative survival of tumor cells after 24 h co‐culture at E:T ratios of 1:1, 5:1, and 10:1. (D) Representative crystal violet staining and corresponding quantification of residual living cancer cells in MDA‐MB‐468 and MDA‐MB‐231 cells. (E) WB analysis of SLC25A13, Bcl‐2, and Bax in MDA‐MB‐468 and MDA‐MB‐231 cells under the indicated co‐culture conditions. (F,G) Relative LDH release and IFN‐γ levels in MDA‐MB‐468 (F) and MDA‐MB‐231 (G) co‐culture systems under the indicated conditions, with or without Lip‐1. Statistics. Data are presented as mean ± SD from three independent experiments unless otherwise indicated. Statistical analyses were performed using two‐way ANOVA for the E: T ratio experiments in (B,C) and one‐way ANOVA with appropriate multiple‐comparisons testing for (D), (F), and (G). Immunoblots in (E) are representative of at least three independent experiments. *p < 0.05, **p < 0.01, ***p < 0.001; ns, not significant.

Given that SLC25A13 suppresses ferroptosis through a metabolic‐signaling axis and reinforces tumor cell‐intrinsic resistance, we next asked whether it also affects CD8^+^ T cell‐mediated cytotoxicity. To address this, we established an in vitro co‐culture system using purified human CD8^+^ T cells isolated from peripheral blood, which were activated by CD3/CD28/CD2 stimulation before being co‐cultured with breast cancer cells expressing different levels of SLC25A13 at graded effector‐to‐target ratios (E:T = 1:1, 5:1, and 10:1) for 24 h (Figure 8A). Cell viability analyses showed that, in MDA‐MB‐468 cells, SLC25A13 knockdown significantly potentiated CD8^+^ T cell killing. Tumor cell survival was reduced across all E:T ratios compared with controls (Figure 8B). In contrast, SLC25A13 overexpression in MDA‐MB‐231 cells preserved a greater proportion of living cancer cells under the same co‐culture conditions (Figure 8C), indicating that SLC25A13 diminishes tumor‐cell susceptibility to CD8^+^ T‐cell cytotoxicity.

This phenotype was further supported by long‐term co‐culture assays. In MDA‐MB‐468 cells, SLC25A13 knockdown reduced the number of residual living cancer cells under both basal and T‐cell co‐culture conditions, whereas addition of Liproxstatin‐1 (Lip‐1) partially restored tumor‐cell survival (Figure 8D). Conversely, in MDA‐MB‐231 cells, SLC25A13 overexpression increased the number of surviving cancer cells, and this effect was further maintained in the presence of Lip‐1 (Figure 8D). At the protein level, co‐culture with T cells shifted apoptosis‐related survival markers in a direction consistent with enhanced tumor‐cell vulnerability after SLC25A13 depletion, as reflected by reduced Bcl‐2 and increased Bax, whereas SLC25A13 overexpression maintained a more pro‐survival pattern (Figure 8E).

To further assess whether ferroptosis contributes to these co‐culture phenotypes, we measured LDH release and IFN‐γ levels in the presence or absence of Lip‐1. In MDA‐MB‐468 cells, SLC25A13 knockdown increased LDH release and was associated with higher IFN‐γ levels, and these changes were partially modulated by Lip‐1 (Figure 8F). In MDA‐MB‐231 cells, SLC25A13 overexpression altered LDH release and IFN‐γ readouts, and these effects were likewise influenced by Lip‐1 (Figure 8G). Together, these data indicate that SLC25A13 restrains CD8^+^ T cell‐mediated tumor killing, at least in part through ferroptosis‐associated mechanisms.

### 2.9 Screening a Small‐Molecule Inhibitor Targeting SLC25A13

**FIGURE 9:**
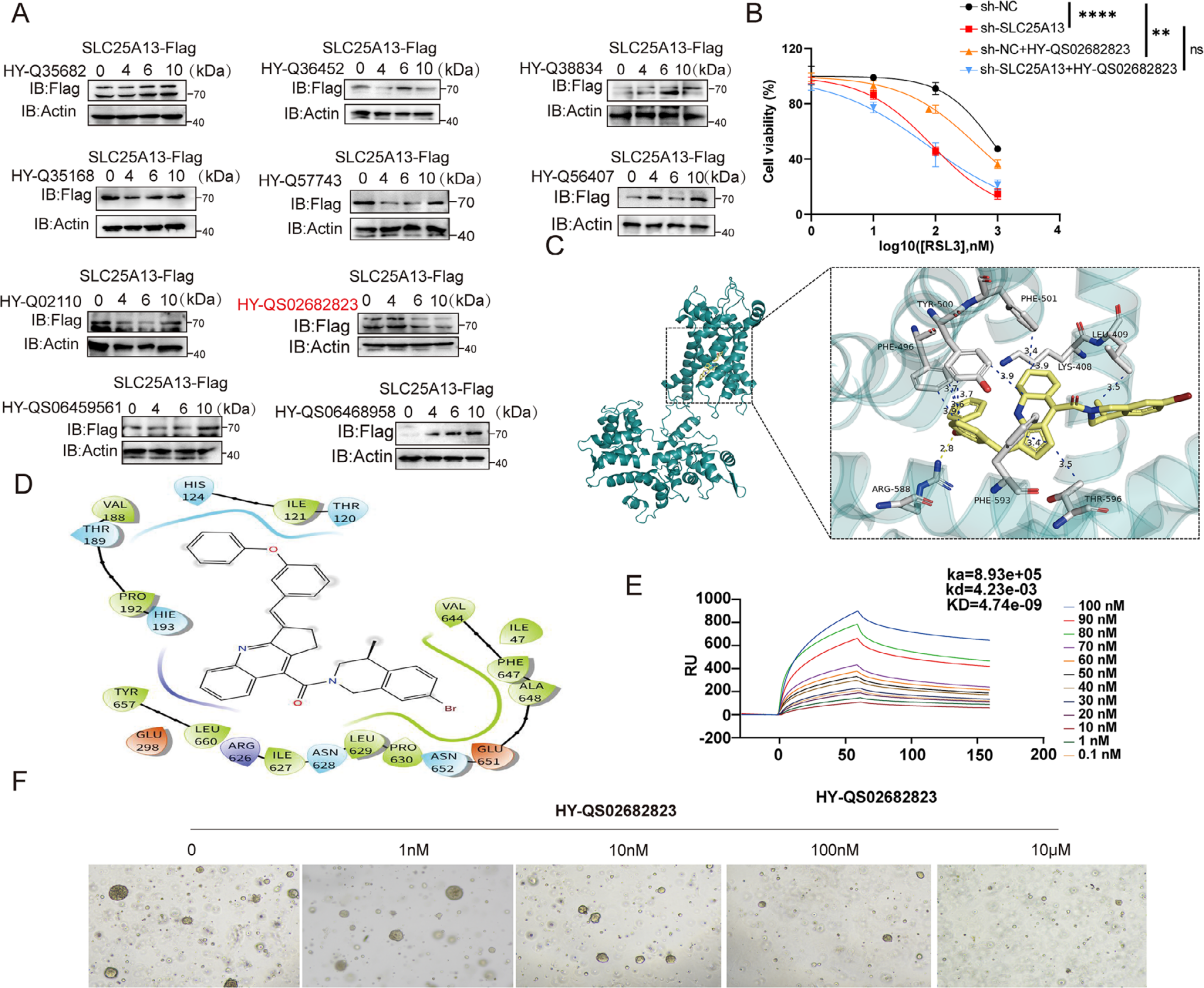
Screening and identification of the small molecule HY‐QS02682823 as a direct SLC25A13‐targeting agent. (A) SLC25A13‐Flag–overexpressing cells were treated with the indicated candidate compounds, and SLC25A13‐Flag protein abundance was assessed to identify molecules that reduce SLC25A13 levels. (B) Effect of HY‐QS02682823 on RSL3 sensitivity in control and SLC25A13‐silenced cells. (C) Overall structural model of the SLC25A13–HY‐QS02682823 complex based on molecular docking, with an enlarged view of the predicted binding pocket. (D) Two‐dimensional interaction map of the HY‐QS02682823–SLC25A13 interface, highlighting key residues and interaction types. (E) SPR sensorgrams showing binding kinetics of HY‐QS02682823 to SLC25A13. (F) Effects of HY‐QS02682823 on breast cancer organoids, assessed after compound treatment.

To identify a selective small‐molecule inhibitor of SLC25A13, we first performed structure‐guided virtual screening of the HY‐L901P compound library using the three‐dimensional structure of SLC25A13. We prioritized the top 10 candidates by predicted binding energy and advanced them to cell‐based validation. In an initial WB screen, most compounds (HY‐Q35682, HY‐Q36452, HY‐Q38834, HY‐Q35168, HY‐Q57743, HY‐Q56407, HY‐Q02110, HY‐QS0648958, and HY‐QS06459561) exerted minimal effects on SLC25A13‐Flag abundance across the 0–10 µm range (Figure 9A). By contrast, HY‐QS02682823 induced a pronounced and dose‐dependent reduction of SLC25A13‐Flag at 4–10 µm. Based on this prominent degrader‐like effect, HY‐QS02682823 was selected as the lead compound for subsequent studies. To further validate the on‐target activity of HY‐QS02682823 in cells, we compared its ferroptosis‐sensitizing effect in control and SLC25A13‐silenced cells. HY‐QS02682823 markedly shifted the RSL3 dose–response curve in control cells, whereas this effect was significantly attenuated after SLC25A13 knockdown, indicating that the ferroptosis‐sensitizing effect of HY‐QS02682823 is largely mediated through SLC25A13 (Figure 9B).

We next defined the binding mode of HY‐QS02682823 to SLC25A13 using complementary in silico and biophysical approaches. Molecular docking predicted that HY‐QS02682823 stably occupies the ligand‐binding cavity of SLC25A13 and engages multiple key residues through hydrogen bonding, hydrophobic contacts, and *π–π* stacking interactions (Figure 9C,D). These features provide a structural rationale for selective recognition of SLC25A13. Surface plasmon resonance (SPR) measurements further revealed canonical concentration‐dependent association and dissociation kinetics over 0.1–100 nm. Kinetic fitting yielded k_a = 8.93 × 10^5^ m ^−^^1^ s^−^^1^, k_d = 4.23 × 10^−^^3^ s^−^^1^, and K_D ≈ 4.74 × 10^−^^9^ m (Figure 9E), indicating nanomolar affinity. Finally, we evaluated HY‐QS02682823 in a three‐dimensional organoid model derived from triple‐negative breast cancer. Treatment caused organoid shrinkage, loosening of architecture, and progressive disintegration in a dose‐dependent manner (Figure 9F). These results suggest that pharmacologic targeting of SLC25A13 is sufficient to impair tumor cell growth and survival at the organoid level.

To evaluate the stability of HY‐QS02682823 binding to SLC25A13 from a structural dynamics perspective, we performed 100‐ns all‐atom molecular dynamics simulations of the complex and constructed free‐energy landscapes using metrics such as RMSD and radius of gyration. The resulting free‐energy surface revealed that the HY–SLC25A13 complex predominantly occupied a single well‐defined low‐energy basin. We did not observe transitions into multiple high‐energy, dissociated states, indicating that the bound pose is thermodynamically stable (Extended Data Figure S6A,B).

Time‐resolved RMSD trajectories further supported this conclusion. After a brief equilibration period, the protein backbone RMSD remained within a narrow fluctuation range. The ligand RMSD was even lower, consistent with stable positioning of HY‐QS02682823 within the binding pocket and minimal drift over time (Extended Data Figure S6C). Residue‐level RMSF analysis showed low thermal fluctuations across most residues, with higher flexibility confined to a few terminal and loop regions (Extended Data Figure S6D). Thus, HY‐QS02682823 binding does not trigger large‐scale conformational rearrangements of SLC25A13.

Hydrogen‐bond kinetics revealed persistent hydrogen bonding between HY‐QS02682823 and SLC25A13 throughout the simulation. These bonds underwent rapid formation–breakage exchange, consistent with a stable yet dynamic interaction network (Extended Data Figure S6E). In parallel, the solvent‐accessible surface area (SASA) and radius of gyration remained largely constant across the trajectory (Extended Data Figure S6F,G), further indicating that the overall fold stays compact and does not undergo unfolding or collapse.

Collectively, the molecular dynamics simulations indicate that HY‐QS02682823 binds SLC25A13 in a manner that is both thermodynamically and kinetically stable. This stable engagement preserves global protein architecture and provides a structural foundation for the compound's ability to promote SLC25A13 degradation and functional inhibition.

### 2.10 HY‐QS02682823 Promotes Lysosome‐Dependent Degradation of SLC25A13

We next used CETSA to validate direct target engagement by HY‐QS02682823 in cells. As the heating temperature was increased stepwise from 37°C to 61°C, SLC25A13‐Flag in the DMSO control rapidly destabilized above 50°C. In contrast, HY‐QS02682823‐treated cells retained substantially higher residual SLC25A13‐Flag signals across the same temperature range. Quantification confirmed that HY‐QS02682823 significantly increased the thermal stability of SLC25A13 (Extended Data Figure S7A,B), supporting specific binding in a cellular context. In agreement with a post‐transcriptional mechanism, qPCR showed that HY‐QS02682823 (0–10 µm) did not measurably alter SLC25A13 mRNA levels (Extended Data Figure S7C). Thus, the compound acts primarily by modulating protein stability rather than transcription. We then asked whether pharmacologic targeting of SLC25A13 broadly sensitizes tumor cells to ferroptosis. Across a diverse panel of cancer cell lines—including murine colorectal cancer MC38, human colorectal cancer HCT116, human cervical cancer HeLa, gastric cancer MKN45, murine melanoma B16‐F10, human lung cancer HCC827, human hepatocellular carcinoma HepG2, and human lung adenocarcinoma A549—co‐treatment with HY‐QS02682823 consistently shifted the RSL3 viability–dose response curves leftward. This resulted in a marked reduction in the effective inhibitory concentration of RSL3 (Extended Data Figure S7D–K). These data indicate that HY‐QS02682823, as an SLC25A13‐targeting inhibitor, confers broad, cross‐tumor sensitization to ferroptosis‐inducing stress.

We next dissected the mechanism by which HY‐QS02682823 reduces SLC25A13 abundance using degradation kinetics and pathway blockade. In cycloheximide (CHX) chase assays, HY‐QS02682823 markedly accelerated the time‐dependent loss of SLC25A13‐Flag relative to vehicle control. By 8 h, the residual protein fraction was substantially reduced (Extended Data Figure S8A,B), indicating that HY‐QS02682823 shortens the SLC25A13 protein half‐life and promotes its degradation.

To define the degradation route, we performed inhibitor rescue experiments. HY‐QS02682823 alone strongly decreased SLC25A13‐Flag levels. This effect was only minimally affected by the proteasome inhibitor MG132. In contrast, the lysosome inhibitor bafilomycin A1 (BafA1) almost restored SLC25A13‐Flag abundance (Extended Data Figure S8C). These data point to a predominantly lysosome‐dependent degradation mechanism. We also asked whether HY‐QS02682823 accompanies ferroptosis sensitization by directly perturbing glutathione. Under both basal conditions and RSL3‐induced ferroptotic stress, HY‐QS02682823 did not significantly alter total cellular GSH levels (Extended Data Figure S8D). Thus, its ferroptosis‐sensitizing activity is unlikely to arise from glutathione depletion. Instead, it primarily reflects interference with SLC25A13 protein stability.

Together, these results indicate that HY‐QS02682823 directly engages SLC25A13 with high affinity and accelerates its lysosome‐dependent degradation. By promoting SLC25A13 degradation, the compound broadly sensitizes diverse tumor cells to ferroptosis inducers. HY‐QS02682823, therefore, represents a lead small molecule with SLC25A13‐targeted degradation potential.

### 2.11 HY‐QS02682823 Exhibits a Favorable In Vivo Safety Profile

Given our in vitro data showing that HY‐QS02682823 directly engages and degrades SLC25A13 and broadly sensitizes tumor cells to ferroptosis inducers, we first evaluated its tolerability in vivo using healthy mice. BALB/c mice received intraperitoneal injections of HY‐QS02682823 (10 or 20 mg/kg) every 2 days for five doses, after which blood and major organs were collected for analysis. Serum biochemistry revealed no evidence of overt toxicity. Levels of the cardiac injury marker CK‐MB, liver function indicators AST and ALT, and the renal function marker creatinine (CR) were comparable between vehicle‐ and HY‐QS02682823‐treated animals at both doses (Extended Data Figure S9A). These results suggest that HY‐QS02682823 does not cause detectable cardiac, hepatic, or renal dysfunction under the tested regimen.

Histopathologic assessment was concordant. H&E staining of heart, liver, lung, spleen, and kidney showed no inflammatory infiltrates, tissue necrosis, or architectural disruption, even at the higher dose of 20 mg/kg (Extended Data Figure S9B). Together, these data indicate that HY‐QS02682823 is well tolerated at effective exposure doses in mice. This safety profile provides a foundation for subsequent in vivo antitumor studies using HY‐QS02682823 as an SLC25A13‐targeting ferroptosis‐sensitizing agent.

### 2.12 HY‐QS02682823 Suppresses Tumor Growth and Lung Metastasis by Activating Ferroptosis and Boosting CD8^+^ T Cell Function

**FIGURE 10:**
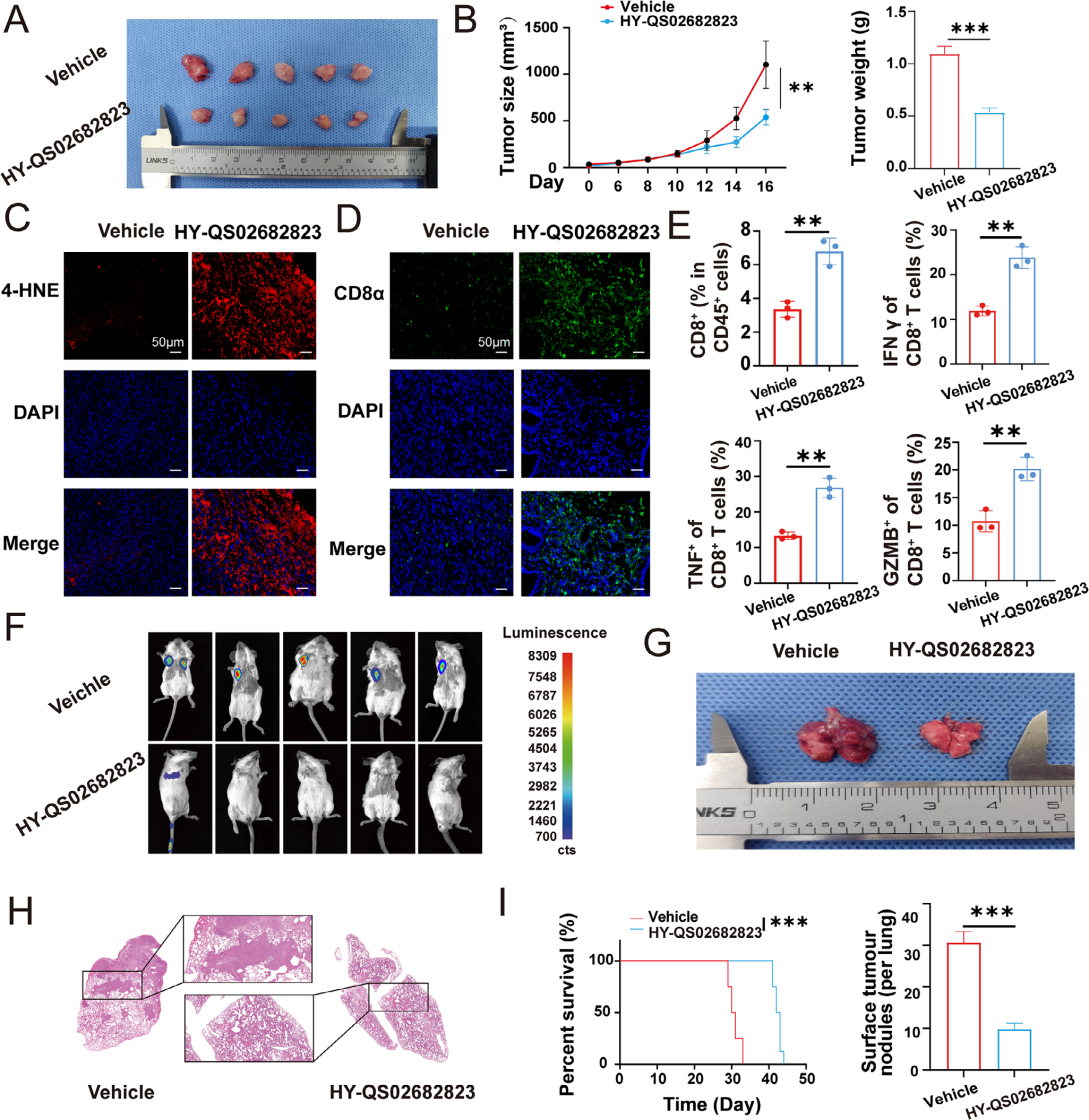
HY‐QS02682823 suppresses tumor growth and lung metastasis by activating ferroptosis and enhancing CD8^+^ T cell function. (A) Representative gross images of orthotopic mammary fat pad tumors from mice treated with vehicle or HY‐QS02682823. (B) Tumor growth curves over time with statistical analysis and endpoint tumor weights. (C) Immunofluorescence staining of the lipid peroxidation marker 4‐HNE in tumor sections, with DAPI nuclear counterstain. (D) Immunofluorescence staining of CD8α^+^ T cells in tumor sections, with DAPI nuclear counterstain. (E) Flow cytometry (or counting‐based analysis) quantifying the fraction of CD8^+^ T cells among CD45^+^ tumor‐infiltrating leukocytes and the frequencies of IFN‐γ^+^, TNF‐α^+^, and GZMB^+^ cells within the CD8^+^ T cell compartment. (F) In vivo bioluminescence imaging of mice in the lung metastasis model treated with vehicle or HY‐QS02682823; the color scale indicates tumor burden intensity. (G) Representative gross images of lungs and surface metastatic nodules from each group. (H) Whole‐lung H&E scans showing the extent and number of metastatic lesions; boxed regions highlight representative foci. (I) Left, Kaplan–Meier survival curves for vehicle‐ and HY‐QS02682823‐treated mice. Right, quantification of lung surface nodule counts per mouse. Panels (A–I) show representative results from at least three independent biological experiments. Data in bar plots and growth curves in (B), (E), (H), and (I) are presented as mean ± s.d. Tumor volume trajectories over time (B, left) were analyzed by two‐way ANOVA with Tukey's post hoc test. Endpoint tumor weight (B, right), CD8^+^ T cell infiltration and cytokine levels (E), lung surface nodule counts (I, right), and other two‐group bar‐plot comparisons were analyzed using two‐tailed unpaired Student's *t*‐tests. Survival differences (I, left) were assessed by the log‐rank (Mantel–Cox) test. *p < 0.05, ** *p* < 0.01, *** *p* < 0.001.

After defining a tolerable dosing window, we evaluated the antitumor activity of HY‐QS02682823 in an immunocompetent orthotopic mammary fat pad tumor model. Compared with the vehicle, HY‐QS02682823 significantly slowed tumor growth over time and reduced endpoint tumor weight (Figure 10A,B), indicating durable in vivo efficacy.

Mechanistically, immunofluorescence staining of tumor sections showed a strong increase in the lipid peroxidation marker 4‐HNE following HY‐QS02682823 treatment (Figure 10C), consistent with robust activation of ferroptosis‐associated oxidative damage. In parallel, HY‐QS02682823 increased the abundance of tumor‐infiltrating CD8^+^ T cells (Figure 10D). Flow cytometry further revealed higher frequencies of IFN‐γ^+^, TNF‐α^+^, and GZMB^+^ cells within the CD8^+^ T cell compartment (Figure 10E). Thus, HY‐QS02682823 not only heightens tumor cell susceptibility to ferroptosis but also strengthens effector CD8^+^ T cell cytotoxicity.

We next tested metastatic control in a lung metastasis model. In vivo bioluminescence imaging showed markedly reduced whole‐body and pulmonary tumor signals in HY‐QS02682823‐treated mice (Figure 10F). Gross inspection and enumeration of lung surface nodules confirmed a significant decrease in metastatic burden, both in number and size (Figure 10G,H). Consistently, HY‐QS02682823 prolonged survival in tumor‐bearing mice and reduced the number of lung surface nodules per animal (Figure 10I). Together, these data indicate that HY‐QS02682823 restrains primary tumor growth and suppresses distant lung metastasis.

### 2.13 HY‐QS02682823 Couples Ferroptosis and Antitumor Immunity In Vivo

**FIGURE 11:**
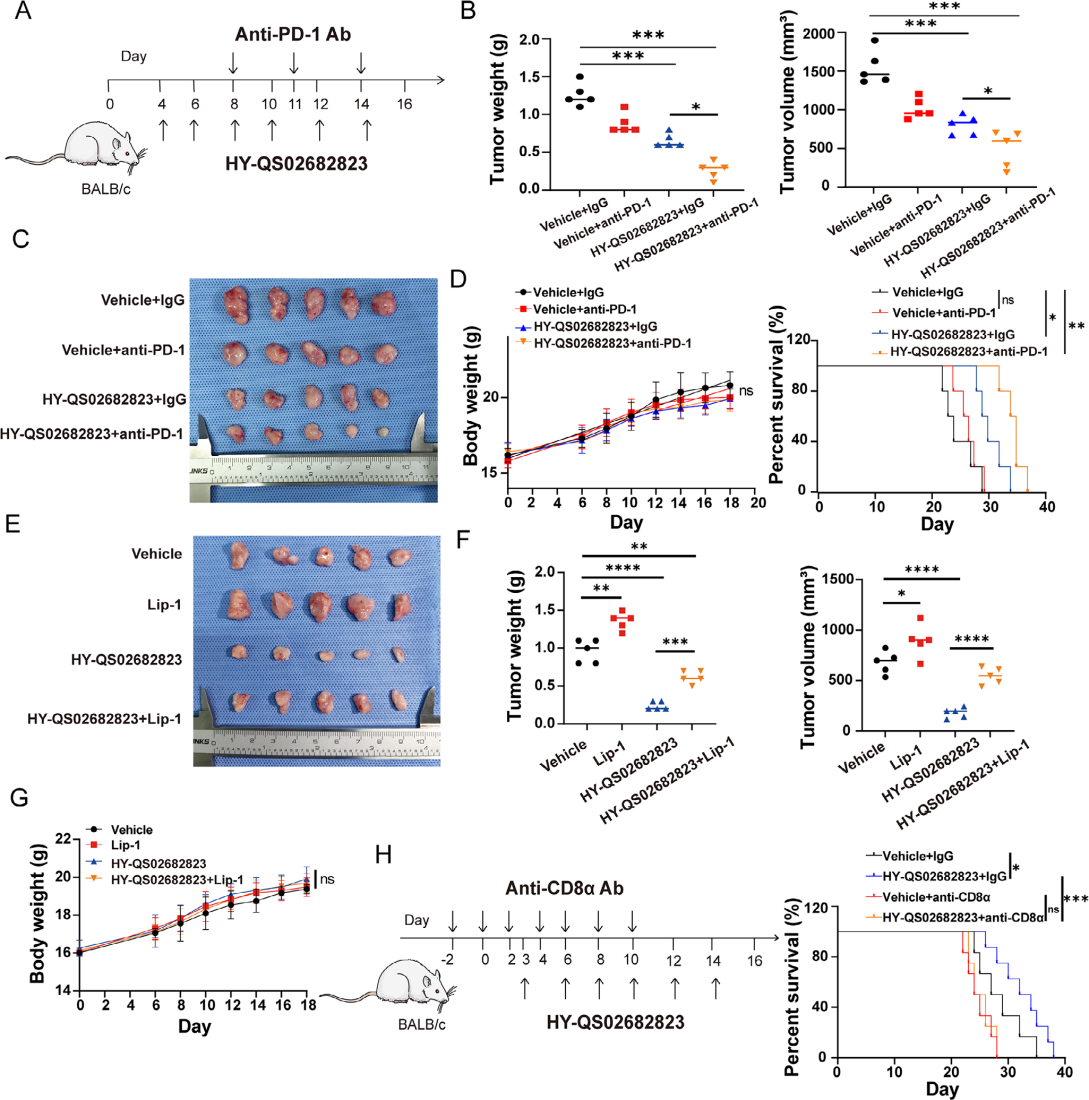
HY‐QS02682823 couples ferroptosis and antitumor immunity in vivo. (A) Schematic illustration of the treatment schedule for the anti‐PD‐1 combination experiment in BALB/c mice. (B) Scatter plots showing final tumor weight and tumor volume in mice treated with Vehicle + IgG, Vehicle + anti‐PD‐1, HY‐QS02682823 + IgG, or HY‐QS02682823 + anti‐PD‐1. (C) Representative images of tumors from the indicated treatment groups in the anti‐PD‐1 combination experiment. (D) Body‐weight curves (left) and Kaplan–Meier survival curves (right) of mice receiving the indicated treatments in the anti‐PD‐1 combination experiment. (E) Representative images of tumors from mice treated with Vehicle, Lip‐1, HY‐QS02682823, or HY‐QS02682823 + Lip‐1. (F) Scatter plots showing final tumor weight and tumor volume in the Lip‐1 rescue experiment. (G) Body‐weight curves of mice receiving the indicated treatments in the Lip‐1 rescue experiment. (H) Schematic illustration of the anti‐CD8α depletion schedule (left) and Kaplan–Meier survival curves (right) of mice treated with Vehicle + IgG, HY‐QS02682823 + IgG, Vehicle + anti‐CD8α, or HY‐QS02682823 + anti‐CD8α. Data are shown as mean ± SD. Tumor weight and tumor volume were analyzed by one‐way ANOVA with appropriate multiple‐comparisons testing. Survival curves were analyzed using the log‐rank (Mantel–Cox) test. ns, not significant. *p < 0.05, **p < 0.01, ***p < 0.001.

To further evaluate the in vivo therapeutic activity of HY‐QS02682823, we first examined its effect in combination with anti‐PD‐1 therapy in a syngeneic BALB/c tumor model (Figure 11A). Compared with vehicle treatment, both anti‐PD‐1 and HY‐QS02682823 alone reduced tumor burden, whereas the combination regimen produced a greater inhibitory effect, as reflected by further decreases in both tumor weight and tumor volume (Figure 11B,C). During treatment, no obvious differences in body weight were observed among groups, indicating acceptable tolerability (Figure 11D, left). In addition, the combination group showed a more favorable survival outcome than either monotherapy (Figure 11D, right). These data indicate that HY‐QS02682823 enhances the antitumor efficacy of PD‐1 blockade in vivo. Histologic analysis of endpoint tumor tissues provided further support for the antitumor activity of HY‐QS02682823 in vivo. Compared with vehicle‐treated tumors, HY‐QS02682823‐treated tumors showed reduced SLC25A13 and Ki‐67 staining, increased 4‐HNE and ACSL4 staining, reduced GPX4 expression, and enhanced CD8^+^ T‐cell infiltration. Similar histologic changes were observed in tumors from the HY‐QS02682823 plus anti‐PD‐1 group (Extended Data Figure S10), consistent with increased lipid peroxidation and greater intratumoral CD8^+^ T‐cell accumulation.

We next asked whether the in vivo activity of HY‐QS02682823 was linked to ferroptosis. As shown in Figure 11E–G, co‐treatment with the ferroptosis inhibitor Lip‐1 partially reversed the tumor‐suppressive effect of HY‐QS02682823, leading to increased tumor weight and tumor volume compared with the HY‐QS02682823‐alone group (Figure 11F). Again, no obvious changes in body weight were detected among treatment groups (Figure 11G). These results support that the antitumor activity of HY‐QS02682823 is at least partly mediated by ferroptosis in vivo.

Finally, to determine whether CD8^+^ T cells contribute to the therapeutic benefit of HY‐QS02682823, we performed CD8α depletion experiments according to the schedule shown in Figure 11H. In the control IgG setting, HY‐QS02682823 prolonged survival relative to vehicle treatment; however, this survival benefit was markedly weakened after CD8^+^ T‐cell depletion (Figure 11H). Together, these findings indicate that the in vivo antitumor effect of HY‐QS02682823 is at least partly dependent on CD8^+^ T cells, consistent with a ferroptosis–immunity coupled mechanism.

## 3 Discussion

Ferroptosis is an iron‐dependent, lipid peroxidation‐driven form of regulated cell death [33, 34, 35]. It has emerged as a key node that reshapes tumor metabolism and the immune microenvironment [36, 37]. In recent years, the crosstalk between ferroptosis and cancer immunity has become a major focus in oncology. Accumulating evidence indicates that CD8^+^ cytotoxic T cells are central effectors of immune checkpoint blockade (ICB) [38]. They can also trigger ferroptosis in tumor cells. This occurs, in part, through IFN‐γ production and rewiring of tumor lipid metabolism, which amplifies lipid peroxidation stress and strengthens antitumor immunity [39]. The relationship is bidirectional. Ferroptosis can also occur in immune cell subsets within the tumor microenvironment, including CD8^+^ T cells [40, 41, 42]. In this context, ferroptosis may blunt immune effector function and reinforce immunosuppression, ultimately limiting therapeutic benefit. Together, these findings highlight a critical principle. Ferroptosis in the tumor microenvironment is not inherently “good” or “bad” [43]. Its outcome depends on where, when, and to what extent ferroptosis is engaged across distinct cellular compartments. Defining the determinants of ferroptosis sensitivity in tumor cells versus immune cells—and developing strategies that selectively sensitize tumor cells to ferroptosis while preserving or enhancing effector immunity—could improve the depth and durability of immunotherapy responses [44, 45]. Yet in triple‐negative breast cancer, the upstream drivers of ferroptosis resistance and their mechanistic coupling to immunosuppression remain poorly defined.

In this study, we systematically investigated the role of the mitochondrial transporter SLC25A13 using public breast cancer datasets and experimental TNBC models. We propose a ferroptosis‐suppressive and immune‐evasive pathway centered on the SLC25A13–STAT3–IFI6 axis. We further provide pharmacologic evidence, using the small molecule HY‐QS02682823, that targeting this axis robustly enhances ferroptosis and antitumor immunity and synergizes with PD‐1 blockade. Across multiple cohorts, we show that SLC25A13 is markedly upregulated in breast cancer. High SLC25A13 expression associates with advanced clinical stage and inferior overall survival. It also tracks with reduced CD8^+^ T cell infiltration, consistent with an “immune‐cold” phenotype. Functional assays in TNBC models support a causal role. SLC25A13 overexpression promotes TNBC cell proliferation, migration, and invasion while suppressing apoptosis, whereas SLC25A13 depletion produces the opposite effects. In syngeneic mouse tumor models, SLC25A13‐high tumors grow faster and display increased Ki‐67 and CD31 staining. In parallel, intratumoral CD8^+^ T cell abundance is reduced, and the fraction of IFN‐γ^+^, TNF‐α^+^, and GZMB^+^ CD8^+^ T cells is diminished. These findings indicate that SLC25A13 not only drives tumor cell‐intrinsic malignant progression but also actively remodels the tumor microenvironment toward immunosuppression.

Our findings identify SLC25A13 as a critical immunometabolic regulator linking mitochondrial adaptation to ferroptosis resistance. Functionally, SLC25A13 suppressed ferroptotic stress by limiting intracellular Fe^2^^+^ accumulation, preserving the GSH/GSSG balance, reducing lipid peroxidation, and maintaining mitochondrial integrity and membrane potential. These effects were evident not only in conventional 2D cell systems but also in patient‐derived organoids, indicating that the ferroptosis‐protective role of SLC25A13 is preserved in a more physiologically relevant context. Together with its association with reduced CD8^+^ T‐cell infiltration and impaired antitumor immunity, these results support the view that SLC25A13 functions as an upstream determinant of both ferroptosis resistance and immune evasion in TNBC.

Mechanistically, we show that this phenotype is mediated through an SLC25A13–STAT3–IFI6 axis. Transcriptomic and functional analyses identified IFI6 as a key downstream effector responsible for the ferroptosis‐protective program, whereas protein‐interaction, epistasis, and transcriptional assays positioned STAT3 as the transcriptional node linking SLC25A13 to IFI6. In this model, SLC25A13 sustains complex I‐dependent oxidative phosphorylation and restrains excessive mitochondrial ROS, thereby promoting STAT3 activation and nuclear translocation, which in turn drives IFI6 transcription. Given the known roles of STAT3 in oxidative stress adaptation and immune suppression, this pathway provides a mechanistic framework connecting mitochondrial metabolism, ferroptosis control, and antitumor immune dysfunction. These findings not only define the biological significance of the SLC25A13–STAT3–IFI6 circuit but also support SLC25A13 as a potential therapeutic target for ferroptosis‐based and immunotherapy‐based combination strategies in TNBC. Although our data support the functional importance of the SLC25A13–STAT3–IFI6 axis in coordinating ferroptosis resistance and immune evasion, the detailed biochemical mechanisms linking SLC25A13 to STAT3 activation and IFI6‐dependent ferroptosis control remain to be further clarified.

Building on these mechanistic findings, we identified HY‐QS02682823 as a candidate small molecule targeting SLC25A13. Biophysical and pharmacologic analyses supported direct engagement of SLC25A13 and indicated that HY‐QS02682823 promotes lysosome‐dependent degradation of the protein, suggesting a targeted‐degrader‐like mode of action rather than simple enzymatic inhibition. Functionally, HY‐QS02682823 enhanced ferroptotic vulnerability, increased lipid peroxidation, and potentiated the antitumor effects of RSL3. In syngeneic TNBC‐like tumor and metastasis models, treatment suppressed tumor progression, prolonged survival, and remodeled the tumor microenvironment toward a more immune‐active state, with increased CD8^+^ T‐cell infiltration and reduced immunosuppressive signaling. Notably, the therapeutic benefit was attenuated by either CD8^+^ T‐cell depletion or ferroptosis inhibition, supporting a ferroptosis–immunity coupled mechanism. Moreover, HY‐QS02682823 showed clear synergy with anti‐PD‐1 therapy and was well tolerated under the tested conditions. Together, these results provide proof of concept that pharmacologic targeting of SLC25A13 may represent a feasible strategy for ferroptosis‐based immunotherapy sensitization.

Of note, this study has several limitations. First, although we define the SLC25A13–STAT3–IFI6 axis as a key mechanism underlying ferroptosis resistance, the precise metabolic programs through which SLC25A13 modulates ferroptosis sensitivity remain incompletely resolved. In particular, whether this effect involves altered aspartate/glutamate shuttling, NADPH homeostasis, or lipid biosynthesis will require further metabolic investigation. Second, while we establish STAT3 as a direct transcriptional activator of IFI6, additional context‐dependent transcriptional regulators may also contribute to this circuitry across distinct breast cancer subtypes. Third, although HY‐QS02682823 showed robust antitumor activity and acceptable tolerability in mice, its off‐target profile and translational applicability require further validation in additional human‐relevant models. Finally, our immune analyses focused mainly on CD8^+^ T cells, whereas the potential contributions of other immune populations, including macrophages, dendritic cells, and regulatory T cells, remain to be defined. These issues warrant further study to refine the mechanistic and translational framework of SLC25A13‐targeted therapy.

A potential limitation of the present study is that the translational interpretation of the in vivo findings may be influenced by species differences between human and mouse SLC25A13. Although our data support a role for the SLC25A13–STAT3–IFI6 axis in ferroptosis resistance and antitumor immunity, interspecies differences in molecular context, protein structure, and pharmacologic susceptibility may affect target dependency and drug response. Thus, the therapeutic activity of HY‐QS02682823 and the immunologic effects of SLC25A13 targeting observed in murine models should be interpreted with caution and will require further validation in human breast cancer specimens, patient‐derived organoids, and other translationally relevant systems. In addition, while our mapping studies localize the interaction to the SH2‐containing region of STAT3, we did not determine whether SLC25A13 is tyrosine phosphorylated or whether this association depends on a canonical phosphotyrosine–SH2 recognition mechanism. Further biochemical studies will be needed to clarify the molecular basis of this interaction.

In summary, our study proposes a model in which the SLC25A13–STAT3–IFI6 axis serves as a central hub coordinating ferroptosis resistance and immune suppression in TNBC. SLC25A13 stabilizes STAT3 and drives a STAT3–IFI6 positive‐feedback transcriptional circuit. This circuit preserves mitochondrial homeostasis, reinforces ferroptosis resistance, and fosters an “immune‐cold” microenvironment characterized by CD8^+^ T‐cell exclusion and functional exhaustion. In contrast, HY‐QS02682823, a small molecule that promotes targeted degradation of SLC25A13, disrupts this pathway, re‐engages tumor cell ferroptosis, and boosts the efficacy of immune checkpoint blockade in syngeneic TNBC‐like models. Together, these findings identify a key metabolic‐to‐transcriptional linkage between ferroptosis resistance and immune evasion in TNBC, while supporting the broader clinical relevance of SLC25A13 across breast cancer cohorts. Further studies in additional breast cancer subtypes will be required to determine whether this mechanism applies beyond TNBC.

## 4 Methods

### 4.1 Ethics Statement

This study complied with all relevant ethical regulations. Human breast cancer specimens were obtained from patients at the Harbin Medical University Cancer Hospital. Written informed consent was obtained from all participants. All procedures conducted were approved by the institutional ethics committee (KY2020‐03). Both male and female patients were included in data collection. Eight‐week‐old female BALB/c mice were purchased from the Laboratory Animal Center of Harbin Medical University (Harbin, China). All mice were specific pathogen‐free (SPF). All animal experiments were performed in accordance with the ethical guidelines of Harbin Medical University.

### 4.2 Public Bulk Transcriptomic Datasets

Bulk RNA‐seq expression profiles and corresponding clinical annotations for breast cancer were obtained from the TCGA‐BRCA cohort. Samples with incomplete survival information were excluded from survival analyses. Gene expression values were processed and analyzed in R (version 4.5.0). For differential expression analysis between tumor and normal breast tissues, we applied DESeq2 to raw count matrices. Differentially expressed genes (DEGs) were defined using the following criteria: |log2FC| ≥ 1 and FDR < 0.05.

### 4.3 Immune Infiltration Analysis (TIMER2.0)

Associations between SLC25A13 expression and immune cell infiltration were evaluated using the TIMER2.0 framework. Correlations between SLC25A13 expression and inferred immune cell abundance were assessed using Spearman's correlation. When multiple immune cell types were tested simultaneously, p‐values were adjusted using the Benjamini–Hochberg method; adjusted p < 0.05 was considered significant. Results were visualized as correlation heatmaps, with color indicating the correlation coefficient and symbols indicating statistical significance as specified in the corresponding figure legends.

### 4.4 Single‐Cell RNA‐Seq Dataset

Public breast cancer single‐cell RNA‐seq (scRNA‐seq) data were retrieved from GEO (accession: GSE176078). The gene‐cell count matrix and metadata were downloaded from the repository and analyzed using Seurat (version 4.5.0) in R. Low‐quality cells were filtered based on standard criteria: cells with <200 detected genes, >6000 detected genes, or >10% mitochondrial transcripts were removed. Genes expressed in fewer than 3 cells were excluded. After filtering, the data were normalized using SCTransform. Highly variable genes were identified, and data were scaled prior to dimensionality reduction.

### 4.5 Dimensionality Reduction, Clustering, and Visualization

Single‐cell RNA‐seq data were processed using Seurat v4.5.0 in R v4.5.0. Data normalization, dimensionality reduction, clustering, and visualization were carried out according to the standard Seurat workflow using default parameters. PCA was applied for dimensionality reduction, and UMAP was used for two‐dimensional visualization. Cluster marker genes were identified using Seurat's differential expression analysis framework.

### 4.6 Cell‐Type Annotation

Major cell populations were annotated based on canonical lineage markers, including CD3D (T cells), CD4 (CD4^+^ T cells), CD8A (CD8^+^ T cells), CD68 (myeloid cells), PECAM1 (endothelial cells), PDGFRB (perivascular cells), MS4A1 (B cells), JCHAIN (plasmablasts), and EPCAM (malignant epithelial cells). Dot plots were used to display marker expression across clusters, where dot size indicates the fraction of expressing cells and color represents normalized expression levels. Feature plots in UMAP space were generated for key markers to support annotation. The proportional composition of major cell types across samples was summarized using donut charts based on the annotated cell identities.

### 4.7 Cell Culture and Reagents

Human breast cancer cell lines MDA‐MB‐231 and MDA‐MB‐468 were maintained in RPMI 1640 medium (Gibco, C11875500BT) supplemented with 10% fetal bovine serum (FBS; Gibco, 10270‐106) and 1% penicillin–streptomycin at 37°C. MDA‐MB‐231 cells were cultured in an air incubator without additional CO_2_, whereas MDA‐MB‐468 and all other cell lines were cultured at 37°C in a humidified incubator with 5% CO_2_. HeLa, HCT116, and A549 cells were obtained from the Heilongjiang Academy of Medical Sciences and cultured in either DMEM (PM150217) or RPMI 1640 (Gibco, C11875500BT), as recommended by the supplier, supplemented with 10% FBS (Gibco, 10270‐106) and 1% penicillin–streptomycin. HepG2, HCC827, B16‐F10, and MC38 cells were purchased from the American Type Culture Collection (ATCC) and maintained under standard conditions in DMEM (PM150217) or RPMI 1640 (Gibco, C11875500BT) supplemented with 10% FBS (Gibco, 10270‐106) and 1% penicillin–streptomycin. Unless otherwise specified, all cell lines were routinely tested and confirmed to be mycoplasma‐free and were used for experiments at 70%–80% confluence.

RSL3 (HY‐100218A; MedChemExpress), ferrostatin‐1 (HY‐100579; MedChemExpress), arachidonic acid (AA; HY‐109590; MedChemExpress), liproxstatin‐1 (T2376; MedChemExpress), and the small‐molecule inhibitor HY‐QS02682823 (MedChemExpress) were purchased from MedChemExpress (MCE). Z‐VAD‐FMK (CM10656) and necrosulfonamide (CM05180) were obtained from Proteintech. Recombinant IFN‐γ was purchased from PeproTech (300‐02). All small‐molecule compounds were dissolved in DMSO to prepare stock solutions and diluted to working concentrations in the appropriate serum‐free medium immediately before use. Equal volumes of DMSO were added to control groups as vehicle controls.

### 4.8 MDA Assay

Intracellular malondialdehyde (MDA) was quantified using a commercial TBARS‐based MDA assay kit (Beyotime, S0131M). After drug treatment, cells were trypsinized and counted; 1 × 10^7^ cells were used per sample and lysed in 100 µL lysis buffer. Lysates were vigorously vortexed, incubated on ice for 30 min, and centrifuged at 12 000 × *g* for 10 min at 4°C; the supernatants were collected. Total protein concentrations were determined by BCA assay to enable normalization. A TBA stock solution was prepared by dissolving 18.5 mg TBA in 5 mL of TBA diluent with vigorous vortexing in a 70°C water bath until fully dissolved. The MDA working reagent was freshly prepared by mixing 150 µL TBA diluent + 50 µL TBA stock + 3 µL antioxidant per reaction. MDA standards were prepared in DEPC‐treated water at 0, 1, 2.5, 5, 10, 20, and 40 µm to generate a standard curve. For each sample, 100 µL of protein supernatant was mixed with 200 µL of MDA working reagent and heated at 100°C for 15 min. After cooling to room temperature, samples were centrifuged at 1000 × *g* for 10 min, and 100 µL of the supernatant was transferred to a 96‐well plate for measurement of absorbance at 532 nm using a microplate reader. MDA concentrations were calculated from the standard curve and normalized to protein content, and results were reported as µmol/mg protein.

### 4.9 Fluorescence Microscopy Assessment of PGSK Fluorescence Intensity

Breast cancer cells were harvested by trypsinization and treated with RSL3 (8 µm) with or without ferrostatin‐1 (Fer‐1, 2 µm) for 24 h. After treatment, a Phen Green SK (PGSK) staining solution was prepared by adding 1 µL PGSK to 1 mL serum‐free (blank) medium. The culture medium was removed, and cells were incubated with the PGSK staining solution under standard culture conditions (37°C, 5% CO_2_) for 25 min. Cells were then washed twice with PBS, after which 2 mL PBS was added to each well, and fluorescence was observed immediately using a fluorescence microscope.

### 4.10 Co‐Immunoprecipitation and WB

Co‐immunoprecipitation (Co‐IP) and WB (Western blot) were performed as previously described with minor modifications [46, 47]. Human breast cancer cell lines MDA‐MB‐231 and MDA‐MB‐468, as well as HEK293T cells used for tagged plasmid expression and high‐level protein production, were used for protein interaction assays. Cells were washed with ice‐cold PBS and lysed on ice in RIPA lysis buffer supplemented with protease inhibitors for 30 min. Lysates were then centrifuged at 12 000 rpm for 30 min at 4°C to remove cell debris, and the supernatants were collected for subsequent Co‐IP or directly used as input controls.

For Co‐IP, equal amounts of total protein were incubated with the indicated primary antibodies at 4°C overnight with gentle rotation, followed by incubation with Protein A/G agarose beads for an additional 2 h at 4°C. Immune complexes were washed five times with PBST (PBS +Triton), and bound proteins were eluted by boiling in 1× SDS loading buffer. Eluted samples and corresponding inputs were separated by SDS–PAGE and transferred onto PVDF membranes. Membranes were blocked with 5% non‐fat milk at room temperature for 1 h, followed by incubation with the appropriate primary antibodies and HRP‐conjugated secondary antibodies. Signals were developed using an enhanced chemiluminescence substrate and recorded using a chemiluminescence imaging system. Band intensities were quantified semi‐quantitatively using ImageJ software. The sources and working dilutions of all primary and secondary antibodies are listed in Extended Data Table S1.

### 4.11 Immunohistochemistry

Immunohistochemistry was performed on paraffin‐embedded human breast cancer tissues and paired adjacent normal breast tissues, as well as on paraffin sections of mouse orthotopic xenograft tumors and lung metastatic lesions. Tissues were fixed in 10% neutral‐buffered formalin, processed routinely, paraffin‐embedded, and sectioned at 4 µm thickness. Sections were baked at 60°C, deparaffinized in xylene, and rehydrated through a graded ethanol series to distilled water. Antigen retrieval was carried out by heat‐induced epitope retrieval in citrate buffer (10 mm sodium citrate, pH 6) or EDTA buffer (pH 9), followed by cooling to room temperature. Endogenous peroxidase activity was blocked with 3% H_2_O_2_, and non‐specific binding was blocked with 5% BSA.

Sections were then incubated overnight at 4°C with the indicated primary antibodies (such as anti‐SLC25A13, anti‐STAT3, anti‐IFI6, anti‐PD‐L1, and anti‐CD8). After washing with PBS containing 0.05% Tween‐20, sections were incubated with HRP‐conjugated secondary antibodies at room temperature for 1 h. Signal was developed using DAB as the chromogen, and nuclei were counterstained with hematoxylin. Slides were dehydrated through graded ethanol, cleared in xylene, and mounted. Images were acquired using a bright‐field microscope. Staining intensity was evaluated semi‐quantitatively using the H‐score method. Detailed information on all IHC antibodies and their working concentrations is provided in Extended Data Table S1.

### 4.12 Hematoxylin‐eosin Staining

Sections were deparaffinized in xylene twice (5 min each) to remove paraffin, and then rehydrated through a graded ethanol series (100%, 95%, 90%, 85%, and 80%), followed by a rinse in PBS. Slides were immersed in hematoxylin solution, and the staining intensity was monitored under a microscope until the nuclei appeared dark blue; excess hematoxylin was removed by rinsing in running tap water. Sections were then incubated in eosin solution, with staining monitored microscopically until the cytoplasm appeared pink to red, and excess dye was washed off with tap water. Afterward, slides were dehydrated through graded ethanol (80%, 85%, 90%, 95%, and 100%), cleared in xylene until fully transparent, and mounted with a coverslip using a suitable mounting medium. Samples were examined under a light microscope and representative images were captured for documentation.

### 4.13 RNA Extraction and Real‐Time PCR Analysis

Total RNA was isolated using an RNA extraction kit (Tiangen) following the indicated treatments. RNA was reverse‐transcribed into complementary DNA (cDNA) using a reverse transcription kit (Tiangen, KR118‐02). Quantitative real‐time PCR was performed using a SYBR Green qPCR master mix (Tiangen, KR123) on a real‐time PCR system, according to the manufacturer's instructions. Reactions were run in technical triplicates. Relative transcript abundance was calculated using the 2^−ΔΔCt^ method and normalized to GAPDH. Primer sequences are provided in Extended Data Table S2.

### 4.14 Establishment of Patient‐Derived Breast Cancer Organoids

Fresh breast tumor specimens were collected immediately after surgical resection under written informed consent and ethics committee approval. Samples were transported on ice in serum‐free basal medium and processed within 2 h. In a biosafety cabinet, tissues were minced into ∼1–2 mm^3^ fragments and digested in a collagenase IV solution (Biosharp, BS165) supplemented with the ROCK inhibitor Y‐27632 at 37°C with shaking for ∼1 h, until the material was largely dissociated into single cells and small clusters.

The digest was then passed through a 70 µm cell strainer to remove undigested debris. The filtrate was washed three times with PBS and centrifuged at 400 g for 5 min. The supernatant was discarded, and the cell pellet was collected and counted. Cells were resuspended in pre‐chilled Matrigel (Corning, 356231) and plated as droplets onto the bottom of pre‐warmed 24‐well plates. Plates were incubated at 37°C for ∼20 min to allow Matrigel polymerization and dome formation. After gelation, pre‐warmed breast cancer organoid culture medium (basal medium supplemented with B27, N2, growth factors, antioxidants, and additional components as required) was added to each well. Organoids were maintained at 37°C in a humidified incubator with 5% CO_2_, and medium was replaced every 2 days. Once organoids formed well‐defined spherical or gland‐like structures with stable growth, they were used for ferroptosis‐related functional assays and drug treatments, including exposure to the SLC25A13‐targeting compound HY‐QS02682823.

### 4.15 Lentiviral Packaging and Generation of Stable Cell Lines

Lentiviral vectors were constructed and packaged by GeneChem (Shanghai, China). All lentiviral transduction procedures were performed in a biosafety cabinet.

For the generation of stable cell lines, MDA‐MB‐231 and MDA‐MB‐468 cells in logarithmic growth were seeded into 6‐well plates. When cultures reached ∼30%–50% confluence, cells were washed twice with PBS and incubated with the calculated volume of lentivirus supplemented with 0.1 mL transduction enhancer (P solution). Complete medium was added to a final volume of 2 mL per well, and cells were cultured for 24 h. The medium was then replaced with fresh complete medium. Stable populations were selected using puromycin (Biosharp, BL528A; 1 µg/mL) for 72 h and maintained under selection until all non‐infected control cells were eliminated. Surviving cells were expanded as stable knockdown or overexpression lines. sh‐NC or empty vector (Vector) populations were used as matched controls in all experiments. Knockdown or overexpression efficiency was validated by qPCR and WB. All stable cell lines were confirmed to be mycoplasma‐free before use in functional and mechanistic assays.

The following plasmids were used in this study: luciferase reporters containing the wild‐type IFI6 promoter (−2000–0 bp; pGL3‐IFI6‐WT) and the corresponding mutant reporter with point mutations introduced at the predicted STAT3‐binding sites P1–P3 (pGL3‐IFI6‐Mut); STAT3 and IFI6 overexpression plasmids for co‐transfection; an SLC25A13 overexpression plasmid (oe‐SLC25A13) and empty vector control (Vector); shRNA plasmids targeting STAT3 (shSTAT3#1 and shSTAT3#2) with a negative control (sh‐NC); and shRNA plasmids targeting SLC25A13 (shSLC25A13#1 and shSLC25A13#2) with a negative control (sh‐NC). All plasmids were constructed by Zhongshi Biotechnology (China).

### 4.16 Transmission Electron Microscopy

To examine ferroptosis‐associated mitochondrial ultrastructural changes, MDA‐MB‐231 and MDA‐MB‐468 cells were collected after treatment with the ferroptosis inducer RSL3 and/or genetic manipulation of IFI6, as indicated. Cells were immediately fixed in 2.5% glutaraldehyde at 4°C overnight. After PBS washes, samples were post‐fixed with 1% osmium tetroxide, dehydrated through a graded ethanol series, and embedded in epoxy resin for polymerization. Ultrathin sections (∼70 nm) were cut using an ultramicrotome, stained with uranyl acetate and lead citrate, and imaged on a transmission electron microscope. Mitochondrial ultrastructure was evaluated by assessing mitochondrial size, cristae integrity, and membrane electron density, which are characteristic features associated with ferroptosis.

### 4.17 Mitochondrial Membrane Potential Assay

Mitochondrial membrane potential (ΔΨm) was assessed using the JC‐1 probe. Briefly, after treatment with RSL3, ferrostatin‐1 (Fer‐1), and/or IFI6 overexpression or knockdown as indicated, cells were gently washed once with PBS and incubated with pre‐warmed JC‐1 working solution (Beyotime, C2003S) at 37°C for 20 min. Cells were then washed twice with JC‐1 buffer and replaced with fresh serum‐free medium.

JC‐1 red fluorescence (aggregates) and green fluorescence (monomers) were captured by fluorescence microscopy or quantified by flow cytometry in the red and green channels. Changes in ΔΨm were expressed as the red/green fluorescence ratio and normalized to the control group.

### 4.18 Mitochondrial Respiration Analysis

MDA‐MB‐231 cells were seeded in Seahorse XF96 cell culture microplates at 1 × 10^4^–2 × 10^4^ cells per well. After overnight attachment, cells were transfected or transduced with the indicated constructs (sh‐STAT3, sh‐SLC25A13, or oe‐SLC25A13), or treated with drugs as specified by the experimental design. Prior to measurement, culture medium was replaced with CO_2_‐independent Seahorse XF assay medium, and plates were equilibrated at 37°C for 1 h.

Oxygen consumption rate (OCR) was measured in real time using a Seahorse XF Analyzer with sequential injections of oligomycin, FCCP, and rotenone/antimycin A. Basal respiration, ATP‐linked respiration (ATP production), maximal respiration, and spare respiratory capacity were calculated according to the manufacturer's instructions and compared across treatment groups.

### 4.19 BODIPY 581/591 C11

Lipid peroxidation was assessed using the fluorescent probe BODIPY 581/591 C11 followed by flow cytometry. Briefly, MDA‐MB‐468 cells (sh‐NC or sh‐SLC25A13/IFI6) and MDA‐MB‐231 cells (Vector or Oe‐SLC25A13/IFI6) were seeded and subjected to the indicated treatments (0.1% DMSO, RSL3 8 µm, or RSL3 8 µm + ferrostatin‐1 (Fer‐1) (2 µm) for 24 h. After treatment, cells were collected by trypsinization, washed twice with PBS, and incubated with BODIPY 581/591 C11 (2 µm) in serum‐free medium) at 37°C for 30 min protected from light. Cells were then washed twice with PBS and resuspended in PBS for immediate analysis on a flow cytometer. BODIPY C11 oxidation was quantified as fluorescence intensity in the FITC/green channel and/or the PE/red channel as indicated, and data were analyzed using FlowJo. Lipid peroxidation levels were reported as mean fluorescence intensity or as the oxidized/reduced fluorescence ratio. At least 10 000 events were collected per sample. Experiments were performed in ≥3 independent biological replicates.

### 4.20 Proximity Ligation Assay

A PLA was performed using the Duolink in situ PLA kit to evaluate the interaction between SLC25A13 and STAT3. Briefly, cells were fixed, permeabilized, and incubated with primary antibodies against SLC25A13 and STAT3. Species‐specific PLUS and MINUS PLA probes were then applied to bind the corresponding primary antibodies. When the two target proteins were in close proximity, the attached oligonucleotides were ligated to form a circular DNA template, which was subsequently amplified by rolling‐circle amplification and detected using fluorescent probes according to the manufacturer's instructions. Images were acquired using a confocal microscope, and PLA puncta were quantified using image analysis software to assess the proximity between SLC25A13 and STAT3.

### 4.21 Nuclear Translocation of STAT3

STAT3 nuclear/cytoplasmic distribution was assessed by combining subcellular fractionation with WB. MDA‐MB‐231 cells were seeded in 6‐well plates and transduced with the indicated lentiviral constructs (sh‐STAT3 or oe‐SLC25A13). Where required, sh‐STAT3 was co‐introduced for rescue experiments. Cytoplasmic and nuclear fractions were prepared using a nuclear and cytoplasmic protein extraction kit according to the manufacturer's instructions. β‐actin and Lamin B1 were used as loading controls for the cytoplasmic and nuclear fractions, respectively. STAT3 abundance in each fraction was analyzed by Western blot.

Dynamic STAT3 nuclear translocation was further examined by immunofluorescence microscopy. Cells were seeded on glass coverslips and treated as indicated. At 0, 30, and 90 min, cells were fixed with 4% paraformaldehyde, permeabilized with 0.1% Triton X‐100, and blocked with 5% BSA. Samples were incubated with an anti‐STAT3 primary antibody at 4°C overnight. After PBS washes, fluorophore‐conjugated secondary antibodies were applied for 1 h at room temperature, followed by DAPI nuclear counterstaining. Images were acquired using a confocal microscope, and STAT3 fluorescence distribution between cytoplasm and nucleus was compared across conditions and time points.

### 4.22 CD8^+^ T‐Cell Isolation, Activation, and Co‐Culture Assay

Human CD8^+^ T cells were isolated from peripheral blood using a human CD8^+^ T‐cell isolation kit (STEMCELL EasySep, Catalog#17953) following the manufacturer's protocol. Peripheral blood mononuclear cells (PBMCs) were first separated from whole blood by density‐gradient centrifugation, and CD8^+^ T cells were subsequently purified by magnetic enrichment. Cell purity was verified by flow cytometry using antibodies against CD3 and CD8. Purified CD8^+^ T cells were activated with a CD3/CD28/CD2 T‐cell activator for 24 h prior to co‐culture. Breast cancer cells were plated in advance, and activated CD8^+^ T cells were added at the indicated effector‐to‐target (E: T) ratios (1:1, 5:1, and 10:1) for 24 h. Tumor cell survival, LDH release, and IFN‐γ levels in the co‐culture system were then analyzed.

### 4.23 Small‐Molecule Screening

To identify candidate small molecules targeting SLC25A13, we performed a focused compound screen using the HY‐L901P library purchased from MedChemExpress and selected 10 candidate molecules for initial screening (HY‐Q35682, HY‐Q36452, HY‐Q38834, HY‐Q35168, HY‐Q57743, HY‐Q56407, HY‐Q02110, HY‐QS02682823, HY‐QS06468958, and HY‐QS06459561). Among them, HY‐QS02682823 was an exploratory small‐molecule candidate identified through virtual screening. Its available physicochemical properties are summarized in Extended Data Table S3.

Stable SLC25A13‐Flag–overexpressing MDA‐MB‐231 cells were seeded in 6‐well plates and cultured to 70% confluence, followed by treatment with each compound at final concentrations of 0, 4, 6, or 10 µm for 24 h. Cells were then lysed, and SLC25A13‐Flag and β‐actin protein levels were analyzed by WB. Compounds were considered preliminary hits if they reduced SLC25A13 protein abundance in a dose‐dependent manner.

To further assess whether candidate compounds sensitized tumor cells to ferroptosis, the indicated cancer cell lines were seeded in 96‐well plates at 3000 cells per well and pretreated with DMSO or the indicated compound for 2 h, followed by co‐treatment with RSL3 over a logarithmic concentration gradient for 24 h. Cell viability was then measured using the MTT assay described above. Ferroptosis sensitization was evaluated based on shifts in the RSL3 dose–response curves in the presence versus absence of the tested compound across multiple tumor cell lines.

### 4.24 Cell Viability Detection

HeLa, MC38, HCC827, B16‐F10, HCT116, HepG2, MDA‐MB‐231, and A549 cells were seeded in 96‐well plates at 3000 cells per well and cultured under standard conditions for 24 h to allow attachment. Cells were then treated with RSL3 and/or HY‐QS02682823 as indicated.

At the end of treatment, MTT reagent (0.5 mg/mL; MedChemExpress, HY‐15924) was added to each well and incubated at 37°C for 2–3 h. The supernatant was removed, and 100 µL dimethyl sulfoxide (DMSO) was added per well with shaking to dissolve the formazan crystals. Absorbance was measured using a microplate reader at 490 nm (test wavelength) and 630 nm (reference wavelength). After blank subtraction, values were normalized to the vehicle control to calculate relative cell viability and to generate dose–response curves.

### 4.25 CETSA

CETSA was performed essentially as previously described with minor modifications [48, 49]. Briefly, cells were harvested by trypsinization, washed twice with ice‐cold PBS and resuspended in PBS at a density of approximately 1 × 10^7^ cells/mL. Aliquots of 50 µL were distributed into PCR tubes and exposed to a temperature gradient ranging from 37°C to 61°C in 3°C increments for 3 min using a thermocycler. Immediately after heating, samples were snap‐frozen in liquid nitrogen and subjected to three freeze–thaw cycles, followed by incubation on ice for 20 min to ensure complete cell lysis. Lysates were clarified by centrifugation at 20 000 g for 10 min at 4°C, and the soluble fractions were collected and mixed with 5× SDS loading buffer. Equal volumes of the supernatants were resolved by SDS–PAGE and analyzed by WB to assess the thermal stability of the indicated proteins.

### 4.26 Organoid Ferroptosis Assay

Patient‐derived breast cancer organoids were maintained in 24‐well plates. Once organoids displayed stable growth, they were treated with arachidonic acid plus IKE (I+A) as indicated. In selected wells, Fer‐1 was co‐administered as a ferroptosis antagonism control. After the specified treatment period, propidium iodide (PI) working solution was added directly to the organoid culture medium. Organoids were incubated at 37°C for 15 min and then gently washed with fresh medium. Bright‐field (BF) and PI fluorescence images were acquired using an inverted fluorescence microscope. Organoid structural integrity and PI‐positive area were quantified across conditions to assess the extent of I+A‐induced ferroptotic death and its reversal by Fer‐1.

### 4.27 Orthotopic Syngeneic Tumor Models With SLC25A13 Manipulation

To evaluate the impact of SLC25A13 on tumor growth, female BALB/c mice were orthotopically inoculated in the right mammary fat pad with murine breast cancer cells stably expressing control shRNA or Oe‐SLC25A13 (1 × 10^5^ cells per mouse) suspended in 100 µL PBS to establish a syngeneic transplantation model. Tumor length and width were measured every 2 days once tumors became palpable, and tumor volume was calculated using the formula 0.52 × length × width^2^. Mice were euthanized at predefined time points or when endpoint criteria were met. Tumors were excised and weighed, and tumor tissues were collected for H&E staining, IHC, IF, and flow cytometry to assess tumor growth and ferroptosis‐related readouts upon SLC25A13 manipulation.

### 4.28 In Vivo Antitumor Efficacy of HY‐QS02682823 Monotherapy

To assess the in vivo antitumor activity of HY‐QS02682823, murine breast cancer cells (2 × 10^5^ cells per mouse in 100 µL PBS) were orthotopically injected into the mammary fat pad of female BALB/c mice. Mice were randomized into a vehicle control group and an HY‐QS02682823‐treated group. HY‐QS02682823 was dissolved in saline containing 2.5% DMSO and administered by intraperitoneal injection (10 mg/kg, every 2 days, for 4–5 doses). Control mice received an equal volume of vehicle. Tumor volume and body weight were monitored at indicated intervals throughout treatment. Mice were euthanized after the final dose, tumors were excised and weighed, and tumors and major organs were harvested for H&E staining, IHC, and IF to evaluate lipid peroxidation (4‐HNE), ferroptosis‐related molecules (ACSL4 and GPX4), and immune‐related markers (SLC25A13, STAT3, PD‐L1, and CD8).

### 4.29 CD8^+^ T Cell Depletion Model

To determine whether the in vivo antitumor efficacy of HY‐QS02682823 depends on CD8^+^ T cells, a CD8^+^ T cell depletion model was established in the syngeneic orthotopic breast cancer model. After tumor implantation, mice were randomized into four groups: Vehicle+IgG, Vehicle+anti‐CD8α, HY‐QS02682823+IgG, and HY‐QS02682823+anti‐CD8α. Anti‐CD8α monoclonal antibody or isotype control IgG was administered intraperitoneally according to a predefined schedule (details are provided in the figure schematic) to achieve sustained depletion of peripheral and intratumoral CD8^+^T cells. HY‐QS02682823 was administered at the dose and frequency described above. Tumor growth was monitored during treatment. At the endpoint, mice were euthanized, tumor weights were recorded, and CD8^+^ T cell depletion/infiltration and antitumor responses were evaluated by flow cytometry and IHC.

### 4.30 Combination Therapy With HY‐QS02682823 and Anti–PD‐1

To evaluate potential synergy between HY‐QS02682823 and immune checkpoint blockade, mice bearing syngeneic orthotopic breast tumors were randomized into four groups: Vehicle+IgG, Vehicle+anti–PD‐1, HY‐QS02682823+IgG, and HY‐QS02682823+anti–PD‐1. HY‐QS02682823 was administered intraperitoneally every 2 days as in the monotherapy regimen. Anti‐PD‐1 antibody or isotype control IgG was administered intraperitoneally according to a predefined dosing schedule (dose and timing are provided in the figure schematic). Tumor volume and body weight were monitored longitudinally. At the endpoint, tumor weights were recorded, and survival was analyzed to assess whether the combination improved tumor control and extended survival without increasing systemic toxicity.

### 4.31 Combination Therapy With HY‐QS02682823 and a Ferroptosis Inhibitor

To test whether the antitumor effect of HY‐QS02682823 in vivo requires ferroptosis, tumor‐bearing mice were randomized into four groups: Vehicle, Liproxstatin‐1, HY‐QS02682823, and HY‐QS02682823+Lip‐1. HY‐QS02682823 was administered intraperitoneally at 10 mg/kg every 2 days. Lip‐1 was prepared in an appropriate vehicle and administered intraperitoneally according to a predefined dose and schedule (details are provided in the figure schematic). Tumor volume and body weight were monitored throughout treatment. At the endpoint, tumors were excised and weighed, followed by H&E staining and molecular marker analyses. Comparisons between the HY‐QS02682823 monotherapy and HY‐QS02682823+Lip‐1 groups were used to determine whether ferroptosis inhibition attenuated the antitumor efficacy and survival benefit of HY‐QS02682823, thereby assessing ferroptosis dependence in vivo.

### 4.32 Statistical Analysis

All experiments were independently repeated three times to ensure data reliability and reproducibility. For comparisons between two groups, a two‐tailed unpaired Student's *t*‐test was used. For comparisons among multiple groups, one‐way analysis of variance (ANOVA) was performed. Data are presented as mean ± s.d. (standard deviation). A *p*‐value < 0.05 was considered statistically significant, with significance levels denoted as follows: not significant (ns), *p < 0.05, ** *p* < 0.01, *** *p* < 0.001, and **** *p* < 0.0001.

## Author Contributions

Y.Z. and Y.T. wrote the first draft of the manuscript. Y.C. and Z.Z. contributed to conception. Y.L., X.W., Q.S., W.Z., and H.P. contributed to review and editing. All authors contributed to manuscript revision, read, and approved the submitted version.

## Funding

This study was funded by the Beijing Medical Award Foundation (Grant Number YXJL‐2023‐0078‐0053). This work was supported by the Beijing Medical Award Foundation (YXJL‑2021‑0424‑0382). This work was supported by the Harbin Medical University Cancer Hospital Haiyan Scientific Research Fund (JJZD2022‑14). This work was supported by the Natural Science Foundation of Heilongjiang Province (PL2024H183).

## Conflicts of Interest

The authors declare that the research was conducted in the absence of any commercial or financial relationships that could be construed as a potential conflict of interest.

## Supporting information




**Supporting File 1**: advs75818‐sup‐0001‐SuppMat.docx.

**Supporting File 2**: advs75818‐sup‐0002‐supplement‐Extended‐Data.zip.

## Data Availability

Data sharing not applicable to this article as no datasets were generated or analysed during the current study.
