# Supplementary material for: Mitochondrial Carrier SLC25A13 Drives Ferroptosis Resistance and Immune Evasion via a STAT3–IFI6 Circuit in Breast Cancer
Source: Adv Sci (Weinh). 2026 May 25:e75818. Online ahead of print. doi: 10.1002/advs.75818 (PMC13336081; doi:10.1002/advs.75818)
Supplement: Supplementary file 1 — Supporting File 1: advs75818‐sup‐0001‐SuppMat.docx. [file ADVS-9999-e75818-s001.docx]

**Extended Data**


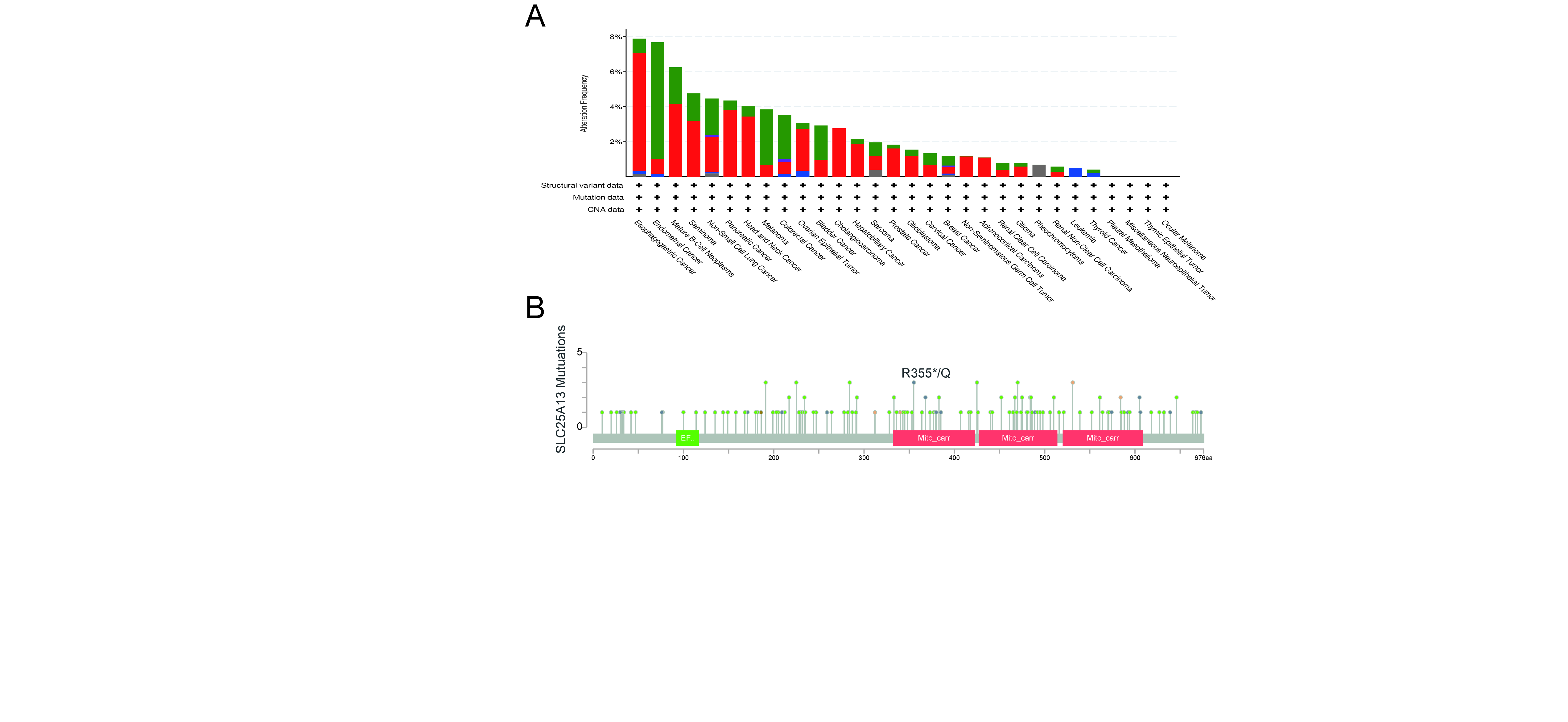


**Extended Data Fig. 1**

**Pan-cancer genomic alteration landscape of SLC25A13.**

(A) Bar plot showing the frequency of SLC25A13 genomic alterations across cancer types, generated from publicly available pan-cancer datasets.

(B) Schematic of the SLC25A13 protein structure with the distribution of mutations mapped along the protein.


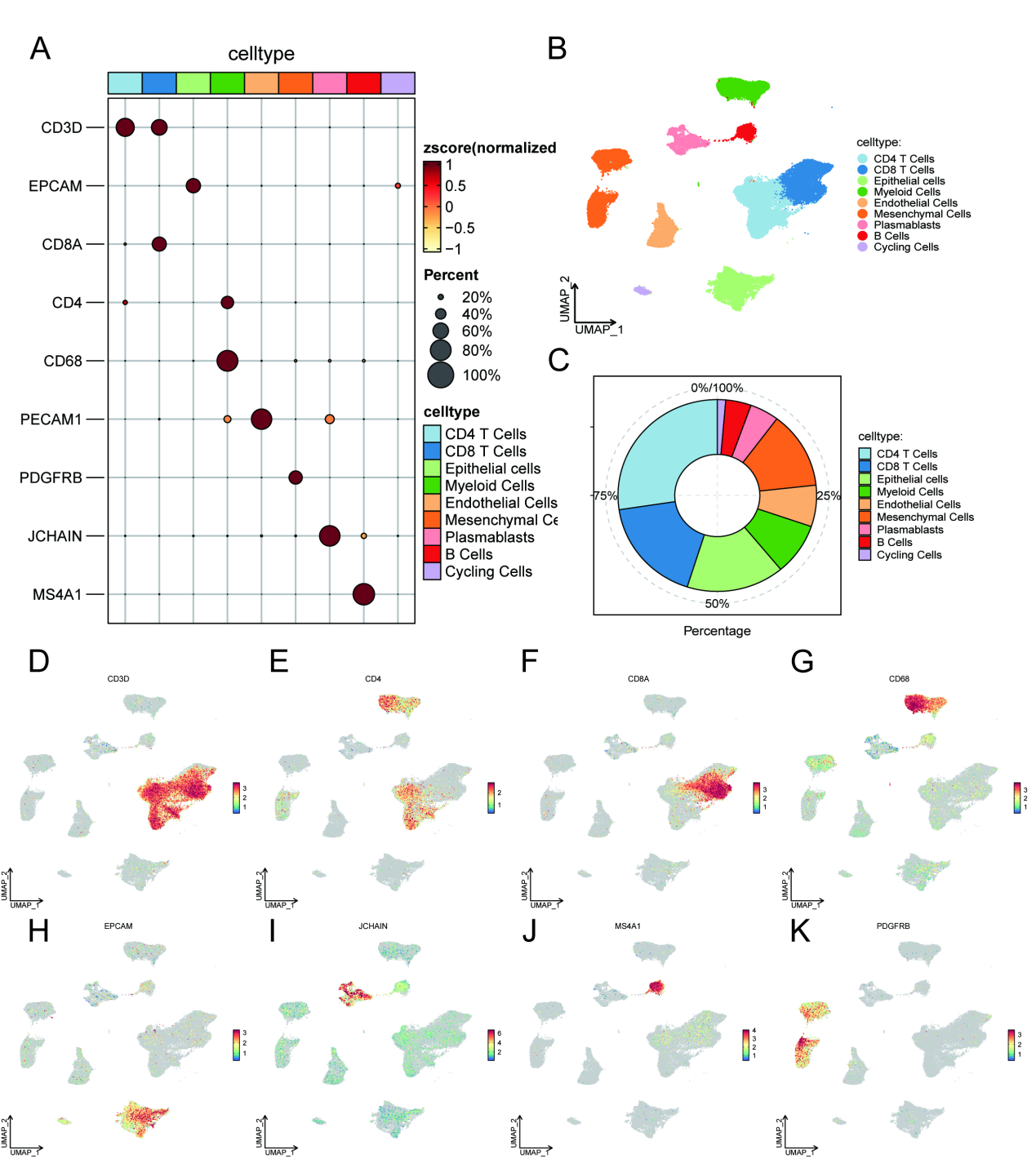


**Extended Data Fig. 2**

**Identification of tumor microenvironment cell types in breast cancer single-cell transcriptomes.**

(A) Dot plot of canonical marker genes (CD3D, CD4, CD8A, CD68, PECAM1, PDGFRB, JCHAIN, MS4A1, EPCAM, etc.) across clusters to distinguish major populations, including CD4⁺/CD8⁺ T cells, myeloid cells, endothelial cells, stromal cells, B cells, plasmablasts, epithelial/malignant epithelial cells, and proliferating cells. Dot size indicates the fraction of expressing cells, and color denotes normalized expression levels.

(B) UMAP visualization of all cells clustered by transcriptomic profiles with cell-type annotations.

(C) Donut chart showing the proportional composition of major cell types across samples.

(D–K) Feature plots in UMAP space showing the expression of key marker genes (CD3D, CD4, CD8A, CD68, EPCAM, JCHAIN, MS4A1, PDGFRB) to support cell-type annotations.


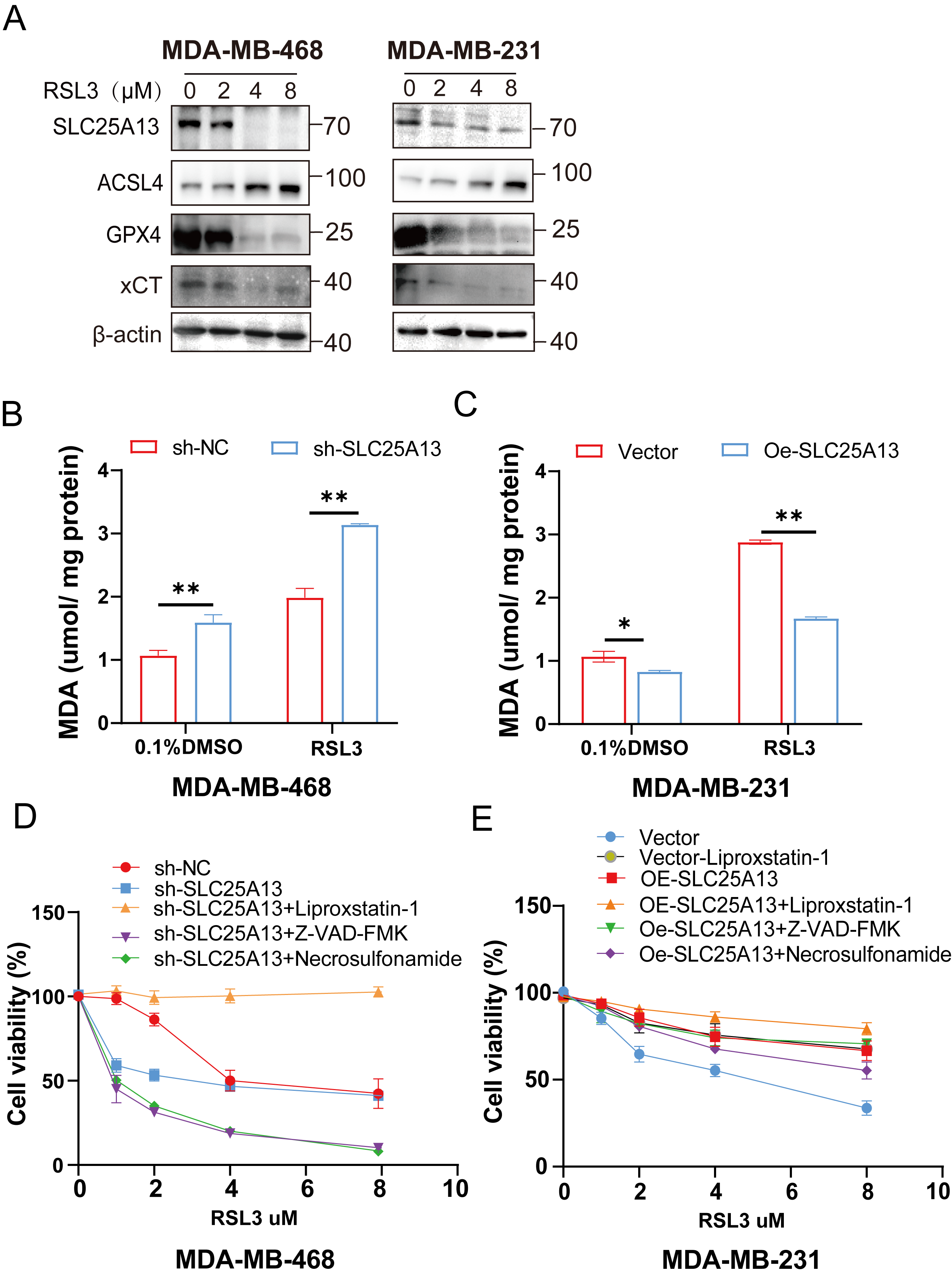


**Extended Data Fig. 3**

**SLC25A13 regulates ferroptosis in breast cancer cells.**

(A) MDA-MB-468 and MDA-MB-231 cells were treated with RSL3 (0, 2, 4, 8 μM) for **24h**, followed by WB analysis.

(B,C) Intracellular malondialdehyde (MDA) levels in MDA-MB-468 and MDA-MB-231 cells upon SLC25A13 knockdown, with or without RSL3 treatment, measured using a TBARS-based commercial MDA assay kit (Beyotime, S0131M) and normalized to total protein content.

1. Cell viability of **MDA-MB-468** cells expressing **sh-NC** or **sh-SLC25A13** under increasing **RSL3** concentrations, with or without **Liproxstatin-1**, **Z-VAD-FMK**, or **Necrosulfonamide**.

(E) Cell viability of **MDA-MB-231** cells expressing **Vector** or **Oe-SLC25A13** under increasing **RSL3** concentrations, with or without **Liproxstatin-1**, **Z-VAD-FMK**, or **Necrosulfonamide**.

All experiments in (A–E) were performed with three biological replicates; data are presented as mean ± s.d. Panel (A) shows a representative WB from three independent experiments. Bar graphs in (B,C) were analyzed by one-way ANOVA with Dunnett’s multiple-comparisons test. Cell viability–dose response curves in (D,E) were analyzed by two-way ANOVA with Dunnett’s multiple-comparisons test.


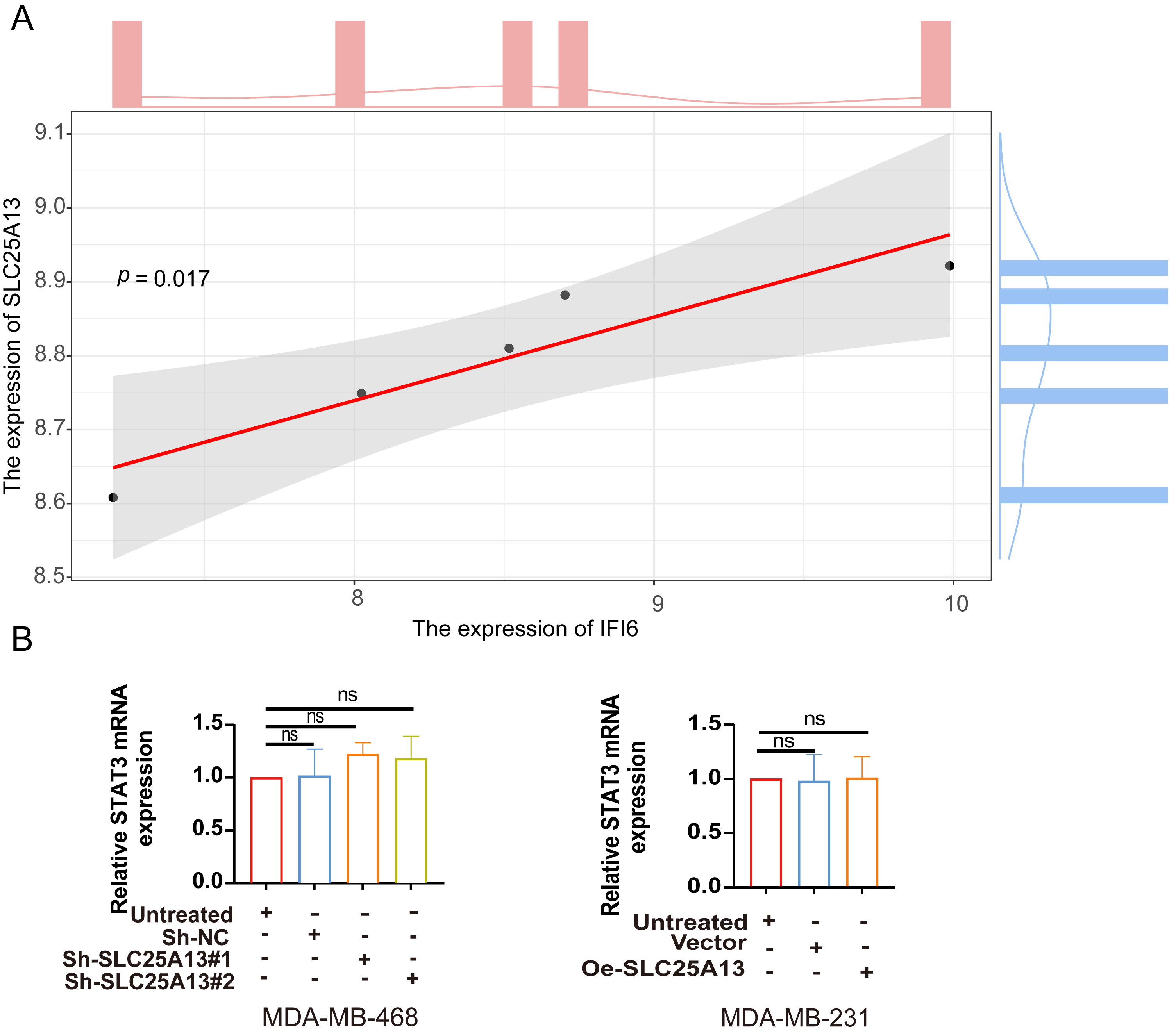


**Extended Data Fig. 4**

**Correlation between SLC25A13 and IFI6 in TNBC and the effect of SLC25A13 on STAT3 mRNA expression**

**(A)** Correlation analysis of **SLC25A13** and **IFI6** expression in the independent **TNBC** dataset **GSE27447**.

**(B)** qPCR analysis of **STAT3 mRNA** levels following **SLC25A13 knockdown** in **MDA-MB-468** cells (left) or **SLC25A13 overexpression** in **MDA-MB-231** cells (right).

Data are presented as mean ± SD from three independent experiments (**n = 3**). Statistical significance was analyzed by **one-way ANOVA with Dunnett’s multiple-comparisons test**. ns, not significant.


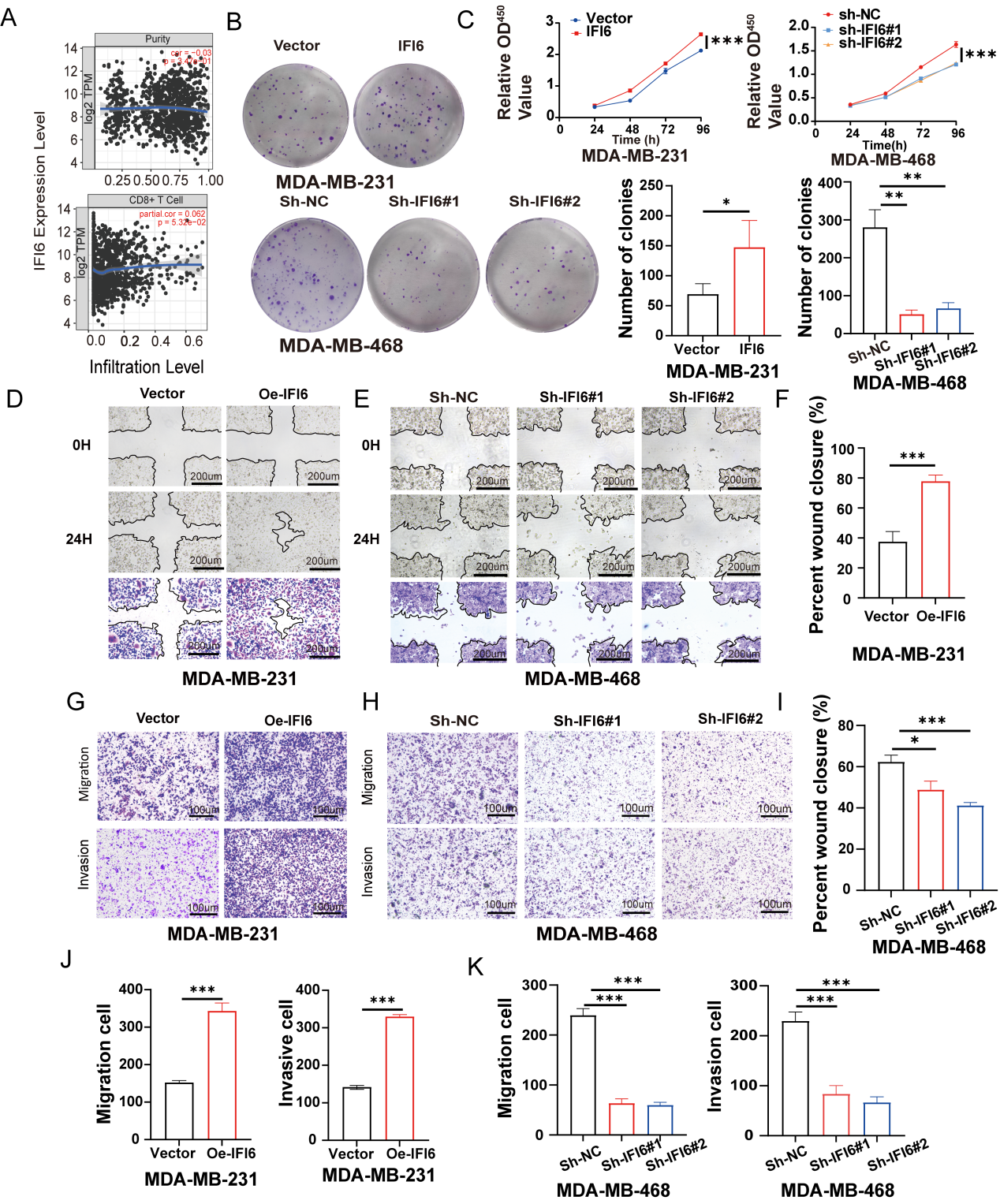


**Extended fig 5**

**IFI6 promotes proliferation, migration, and invasion of breast cancer cells.**

**(A)** Correlation analysis of **IFI6** expression with **tumor purity** (top) and **CD8⁺ T-cell infiltration level** (bottom) in the **TCGA-BRCA** cohort.
**(B)** Representative images of **colony formation assays** in **MDA-MB-231** cells transfected with **Vector** or **IFI6**, and in **MDA-MB-468** cells expressing **sh-NC**, **sh-IFI6#1**, or **sh-IFI6#2**.
**(C)** **Cell proliferation curves** measured by CCK-8 assay (top) and **quantification of colony numbers** from the colony formation assay (bottom) in **MDA-MB-231** and **MDA-MB-468** cells under the indicated conditions.
**(D–E)** Representative images of **wound-healing assays** in **MDA-MB-231** cells expressing **Vector** or **Oe-IFI6** (**D**) and in **MDA-MB-468** cells expressing **sh-NC**, **sh-IFI6#1**, or **sh-IFI6#2** (**E**) at **0 h** and **24 h**. Scale bars, **200 μm**.
**(F, I)** Quantification of **percent wound closure** in **MDA-MB-231** (**F**) and **MDA-MB-468** (**I**) cells under the indicated conditions.
**(G–H)** Representative images of **Transwell migration and invasion assays** in **MDA-MB-231** cells expressing **Vector** or **Oe-IFI6** (**G**) and in **MDA-MB-468** cells expressing **sh-NC**, **sh-IFI6#1**, or **sh-IFI6#2** (**H**). Scale bars, **100 μm**.
**(J–K)** Quantification of **migrated** and **invaded** cell numbers in **MDA-MB-231** (**J**) and **MDA-MB-468** (**K**) cells.

Data are presented as mean ± SD from three independent experiments. The correlation analysis in (A) was performed using Spearman’s correlation. Two-group comparisons were analyzed by two-tailed unpaired Student’s t test, and comparisons among multiple groups were analyzed by one-way ANOVA with Dunnett’s multiple-comparisons test. **P* < 0.05, ***P* < 0.01, ******P* < 0.001.**


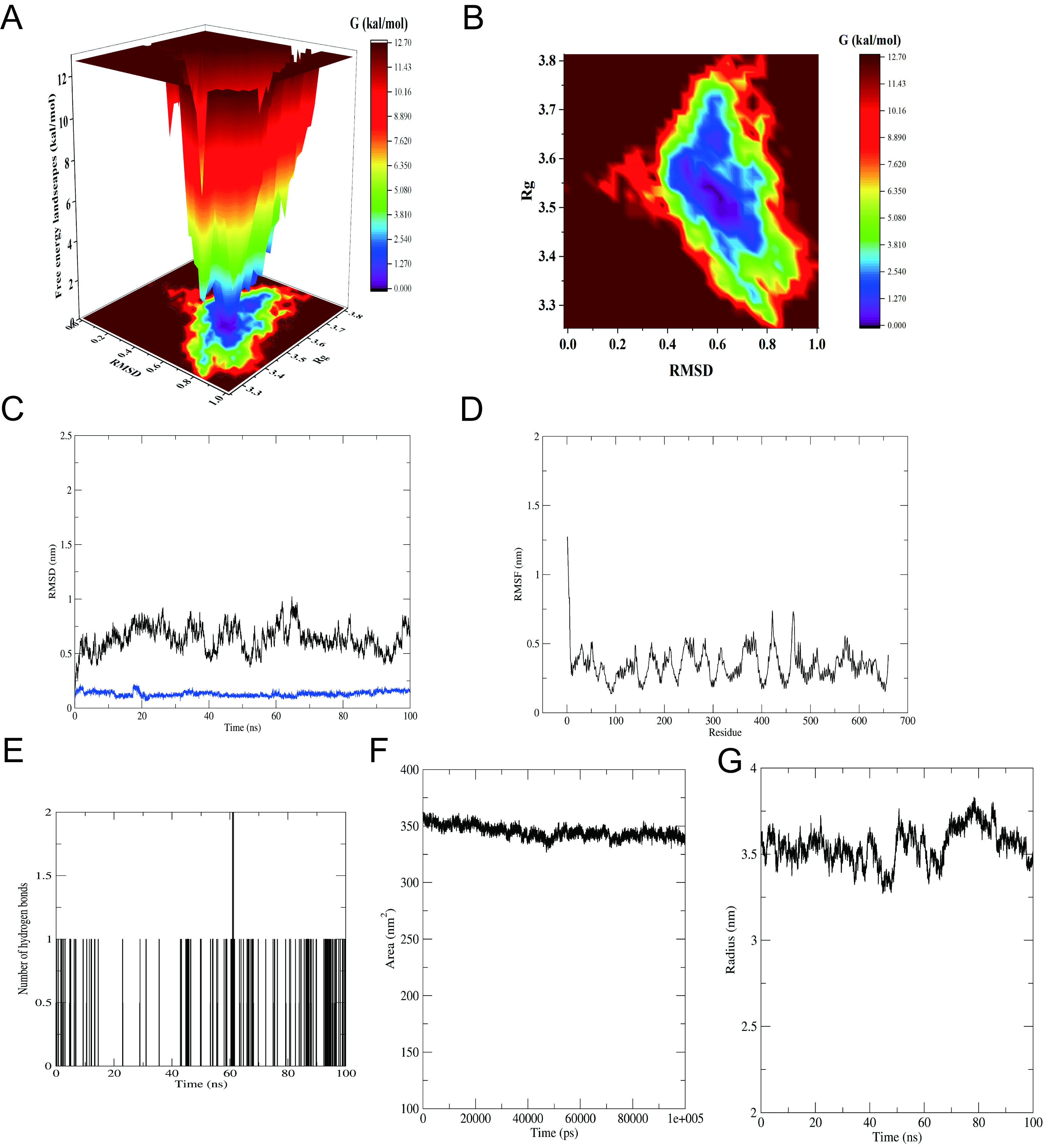


**Extended Data Fig 6**

**Molecular dynamics simulations reveal a stable binding conformation of the HY small-molecule inhibitor in complex with SLC25A13.**

(A) Three-dimensional free-energy landscape constructed using protein backbone RMSD and radius of gyration (Rg) as reaction coordinates.

(B) Two-dimensional free-energy projection on the RMSD–Rg plane, highlighting the dominant conformational region sampled during the simulation.

(C) Time evolution of RMSD, with the protein backbone RMSD (black) and ligand HY RMSD (blue), indicating limited fluctuations after a brief equilibration phase.

(D) Per-residue RMSF profile, reflecting residue-wise flexibility during the simulation; most residues show low fluctuations, with higher mobility mainly at the termini and selected loop regions.

(E) Time course of hydrogen bonds between HY and SLC25A13 (y-axis, number of hydrogen bonds), illustrating dynamic formation and maintenance of the interfacial hydrogen-bond network over 100 ns.

(F) Solvent-accessible surface area (SASA) of the complex over time, indicating overall stable solvent exposure throughout the simulation.

(G) Radius of gyration (Rg) of the complex over time, suggesting that the protein remains compact without pronounced unfolding.


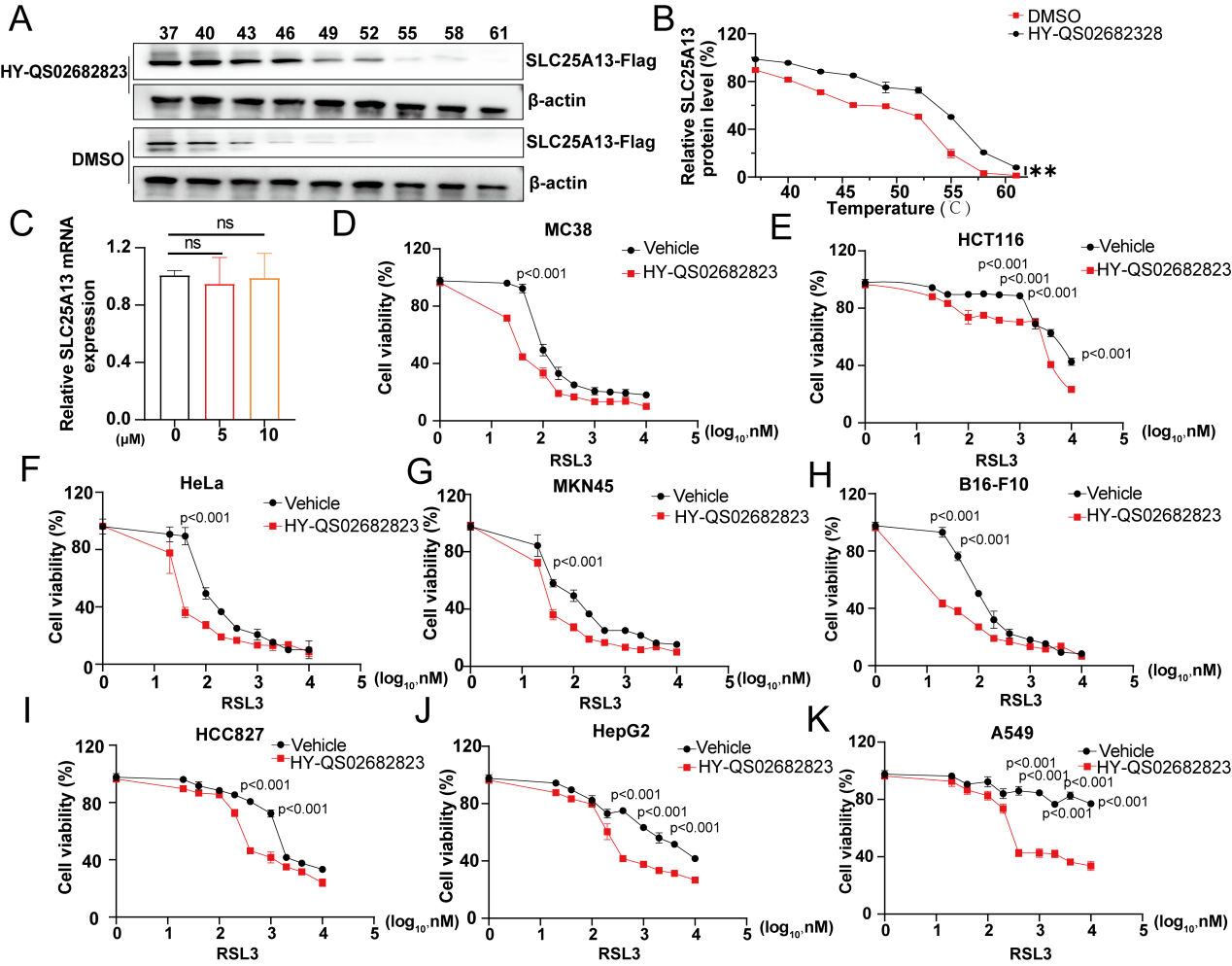


**Extended Data Fig. 7**

**HY-QS02682823 directly targets SLC25A13 with high affinity and potentiates RSL3-induced ferroptosis across multiple tumor cell lines.**

(A) CETSA in cells stably expressing SLC25A13-Flag treated with DMSO or HY-QS02682823, followed by heating at the indicated temperatures (37–61 °C) and WB to detect residual SLC25A13-Flag protein.

(B) Densitometric quantification of (A), plotted as the relative SLC25A13 protein abundance versus temperature to compare thermal stabilization by HY-QS02682823 versus DMSO.

(C) qPCR analysis of SLC25A13 mRNA levels after 24 h treatment with HY-QS02682823, showing no significant effect on SLC25A13 transcription.

(D–K) Dose–response curves for RSL3 after 24 h treatment in mouse- or human-derived tumor cell lines pretreated with vehicle or HY-QS02682823: MC38 (D), HCT116 (E), Hela (F), MKN45 (G), B16-F10 (H), HCC827 (I), HepG2 (J), and A549 (K), as assessed by cell viability assays. Red curves indicate HY-QS02682823 + RSL3, and black curves indicate vehicle controls.

Data in (B,C) are presented as mean ± s.d. and were analyzed by two-tailed unpaired Student’s t test. Dose–response curves in (D–K) are based on three independent experiments and were analyzed by two-way ANOVA followed by Dunnett’s multiple-comparisons test. ns, not significant. **P* < 0.05, ***P* < 0.01, ****P* < 0.001.


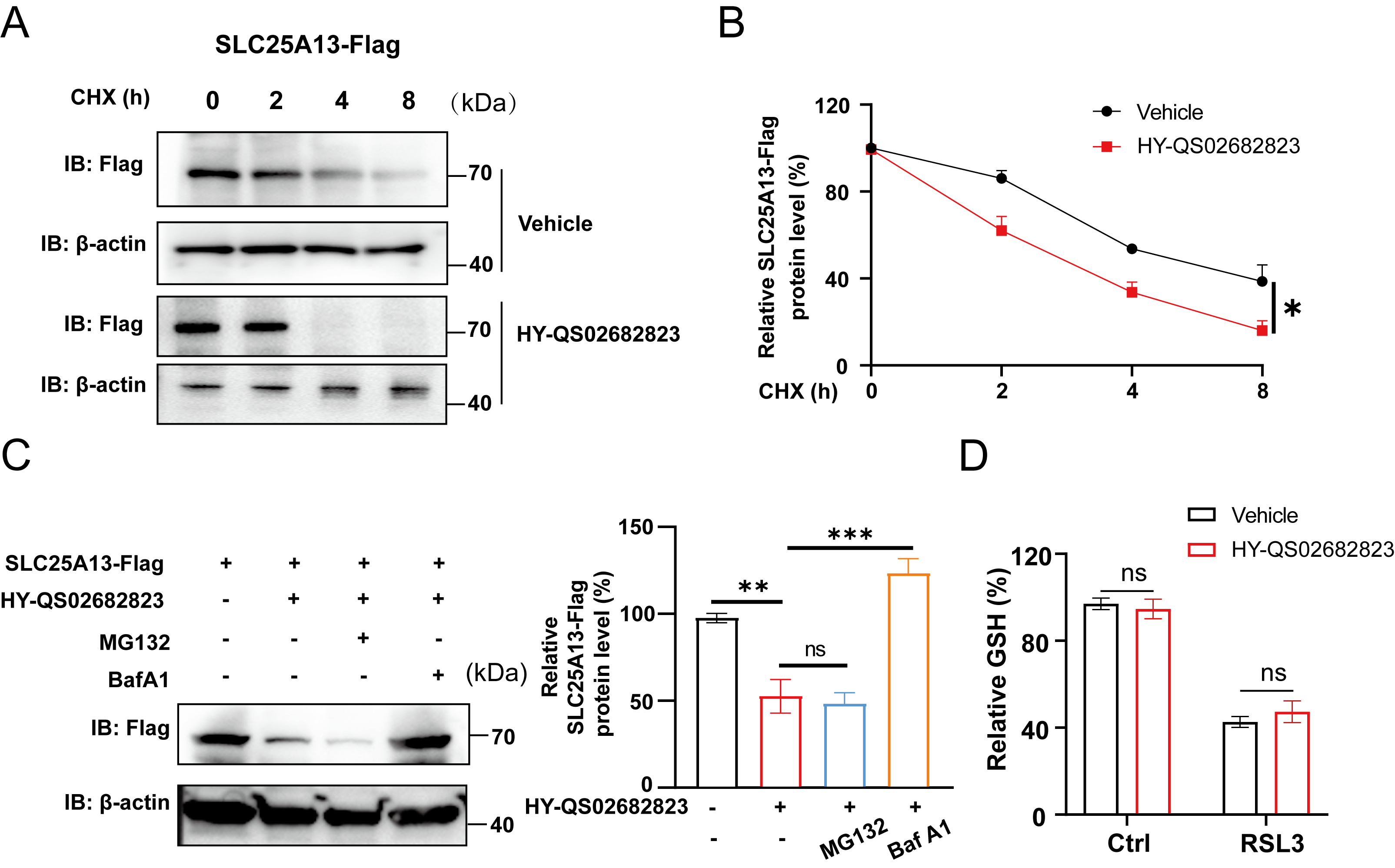


**Extended Data Fig. 8**

**HY-QS02682823 promotes SLC25A13 protein degradation predominantly via the lysosomal pathway.**

(A) Cycloheximide (CHX) chase assay in cells overexpressing SLC25A13-Flag.

(B) Densitometric quantification of SLC25A13-Flag in (A) after normalization, plotted as relative protein abundance over the CHX chase time course.

(C) Cells overexpressing SLC25A13-Flag were treated for 8 h with MG132 (proteasome inhibitor) or BafA1 (lysosome inhibitor) in the absence or presence of HY-QS02682823 (10 μM).

(D) Relative intracellular GSH levels in vehicle- or HY-QS02682823-treated cells under basal conditions or following RSL3 treatment.

Panel (A) shows a representative WB. Quantitative data in (B-D) are presented as mean ± SD. Statistical analysis for (B) and (D) was performed using two-way ANOVA followed by Dunnett’s multiple-comparisons test. Statistical analysis for (C) was performed using one-way ANOVA followed by Dunnett’s multiple-comparisons test. ns, not significant. **P* < 0.05, ***P* < 0.01, ****P* < 0.001.


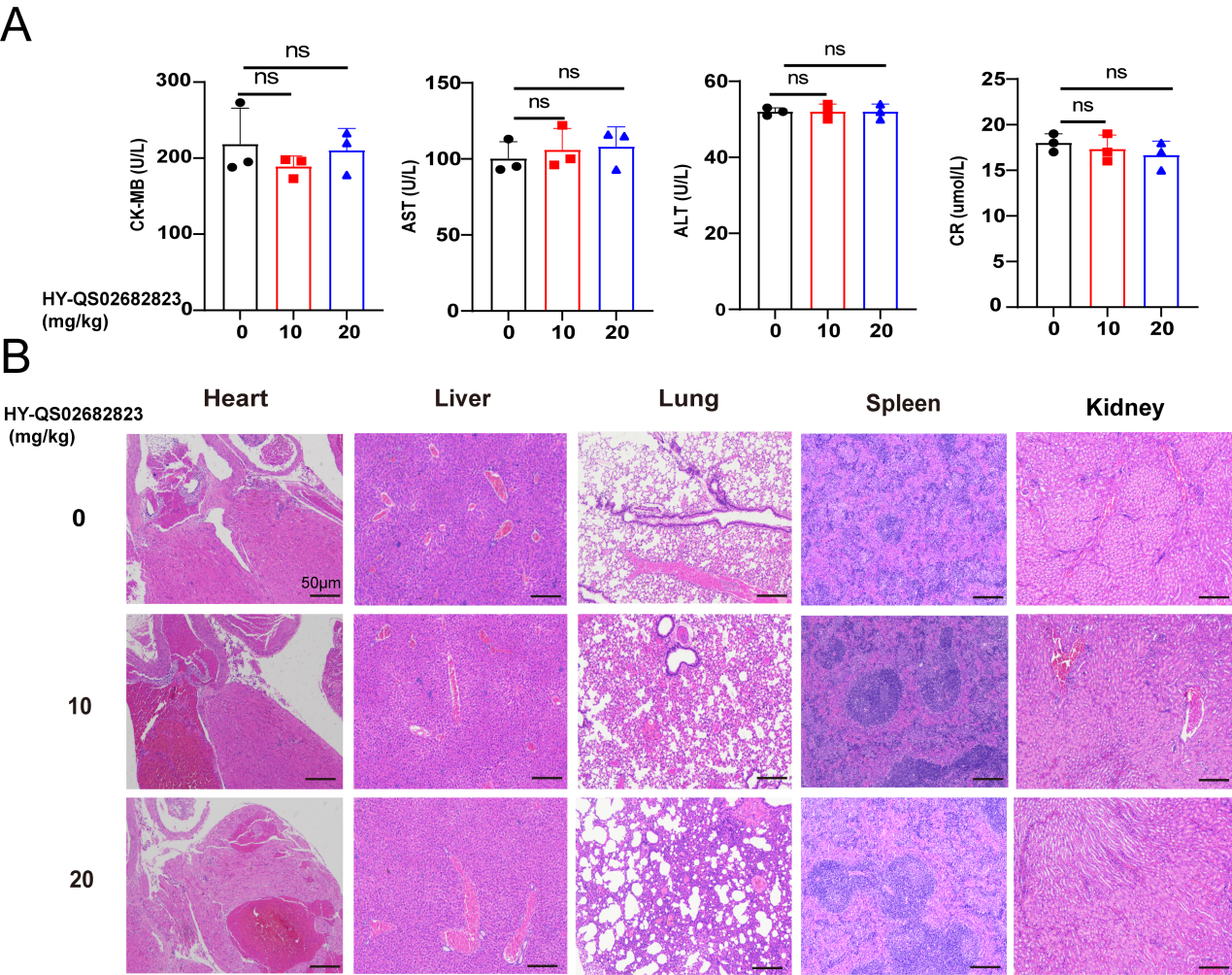


**Extended Data Fig. 9**

**HY-QS02682823 exhibits no detectable cytotoxicity in vivo.**

(A) Serum levels of alanine aminotransferase (ALT), aspartate aminotransferase (AST), creatinine (CRE), and creatine kinase isoenzyme (CK-MB) in mice following HY-QS02682823 administration.

(B) Representative hematoxylin and eosin (H&E) staining of mouse tissues (heart, liver, spleen, lung, and kidney) after HY-QS02682823 treatment. Scale bar, 200 μm. Data in (B) are representative of three independent mice.

For (A), data are presented as mean ± s.d. with n = 3 mice per group and were analyzed by one-way ANOVA followed by Dunnett’s multiple-comparisons test. ns, not significant.


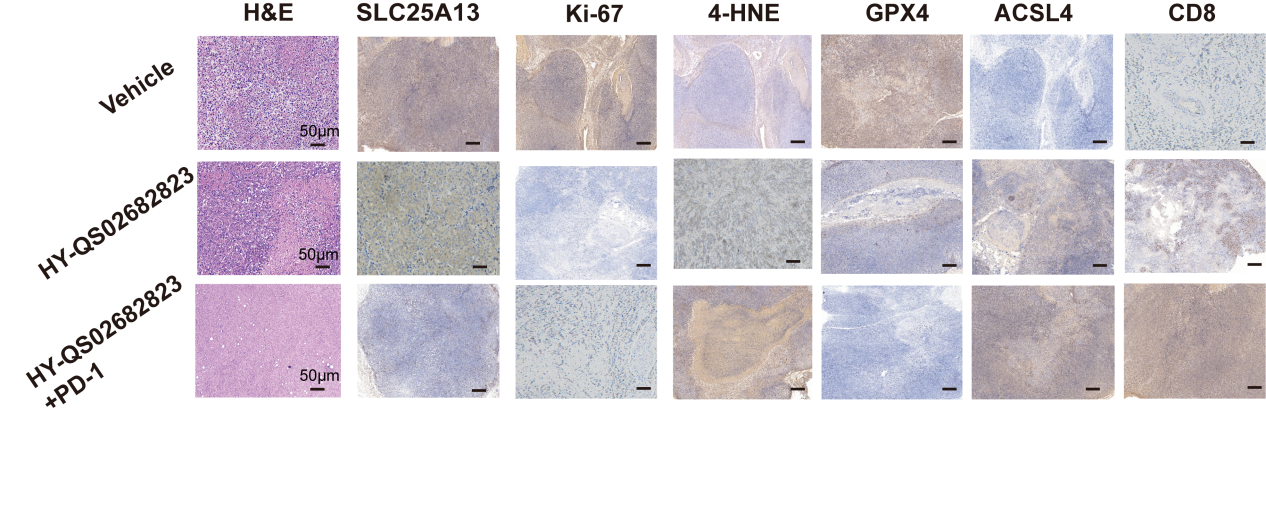


**Extended Data Fig. 10**

**H&E and IHC analysis of tumor tissues after HY-QS02682823 treatment alone or in combination with anti-PD-1**Representative **H&E** and **IHC** images of endpoint tumor tissues from mice treated with **Vehicle**, **HY-QS02682823**, or **HY-QS02682823 + anti-PD-1.** (Scale bars, **50 μm**)

**Extended data Table 1 List of antibodies used in this project.**

| **Extended data Table 1. List of antibodies used in this project** | | | | |
| --- | --- | --- | --- | --- |
| **Name** | **Vendor** | **Cat.** | **Application** | **Dilution** |
| Anti-SLC25A13 | Abcam | ab96303 | IB | 1:1000 |
| Anti-SLC25A13 | Proteintech | 10789-1-AP | IB/IF/IHC | 1:1000 |
| Anti-STAT3 | Starter | S-1393-64 | IB | 1:1000 |
| Anti-STAT3 | Proteintech | 10253-2-AP | IP/IHC | 1:1000 |
| Anti-GPX4 | Abclonal Technology | A11243 | IB | 1:1000 |
| Anti-ACSL4 | Proteintech | 22401-1-AP | IB | 1:10000 |
| Goat anti-mouse secondary HRP antibody | Invitrogen | A16072 | IB | 1:5000 |
| Goat anti-rabbit secondary HRP antibody | Invitrogen | A16104 | IB | 1:5000 |
| HRP-anti-HA | Roche Applied Science | 12994 | IB | 1:5000 |
| HRP-anti-Flag | Proteintech | 66008-4-Ig | IB | 1:20000 |
| HRP-anti-His | Proteintech | 66005-1-Ig | IB | 1:10000 |
| Anti-β-actin | Sigma-Aldrich | A1978 | IB | 1:3000 |
| Anti-4 Hydroxynonenal | Abcam | ab48506 | IF | 1:150 |
| Anti-CD8 alpha | Servicebio | GB114196-100 | IF | 1:200 |
| APC anti-mouse CD3ε Antibody | Biolegend | 152306 | FACS | 1:200 |
| FITC anti-mouse CD8a Antibody | BioLegend | 100706 | FACS | 1:200 |
| PE/Cyanine7 anti-mouse CD45 Recombinant Antibody | BioLegend | 157614 | FACS | 1:200 |
| APC/Cyanine7 anti-mouse IFN-γ Antibody | BioLegend | 505850 | FACS | 1:200 |
| PE/Cyanine7 anti-mouse TNF-α Antibody | BioLegend | 506324 | FACS | 1:200 |
| PE/Cyanine7 anti-human/mouse Granzyme B Recombinant Antibody | BioLegend | 372214 | FACS | 1:200 |
| FITC anti-mouse/rat/human CD27 Antibody | BioLegend | 124208 | Isolation | 1:100 |
| Rat IgG2b isotype control-InVivo (clone LTF-2) | Selleck | A2106 | In vivo test | / |
| Anti-mouse CD8α-InVivo (clone 2.43) | Selleck | A2102 | In vivo test | / |
| InVivoMAb rat IgG2a isotype control, anti-trinitrophenol (clone 2A3) | BioXCell | BE0089 | In vivo test | / |
| InVivoMAb anti-mouse PD-1 (CD279) (clone RMP1-14) | BioXCell | BE0146 | In vivo test | / |

**Extended data Table 2 Primer sequences.**

| Name | Sequence |
| --- | --- |
| SLC25A13-F | AGATGGTTCGGTCCCACTTGCA |
| SLC25A13-R | ACCAGTGGTGATTTCTCCTGCC |
| STAT3-F | CAGCAGCTTGACACACGGTA |
| STAT3-R | AAACACCAAAGTGGCATGTGA |
| IFI6-F | GGTCTGCGATCCTGAATGGG |
| IFI6-R | TCACTATCGAGATACTTGTGGGT |
| GAPDH-F | GGTGTGAACCATGAGAAGTATGA |
| GAPDH-R | GAGTCCTTCCACGATACCAAAG |

**Extended data Table 3 Physicochemical properties of HY-QS02682823**

| Parameter | Description |
| --- | --- |
| molecular formula | C36H29BrN2O2 |
| molecular weight | 601.53 |
| CAS number | Not assigned |
| Purity | 90% |
| Solubility | Soluble in DMSO |
| Stability | DMSO stock solutions were freshly prepared or stored at −80°C for short-term use |
