# Supplementary figures and images for: Mitochondrial Carrier SLC25A13 Drives Ferroptosis Resistance and Immune Evasion via a STAT3–IFI6 Circuit in Breast Cancer
Source: Adv Sci (Weinh). 2026 May 25:e75818. Online ahead of print. doi: 10.1002/advs.75818 (PMC13336081; doi:10.1002/advs.75818)

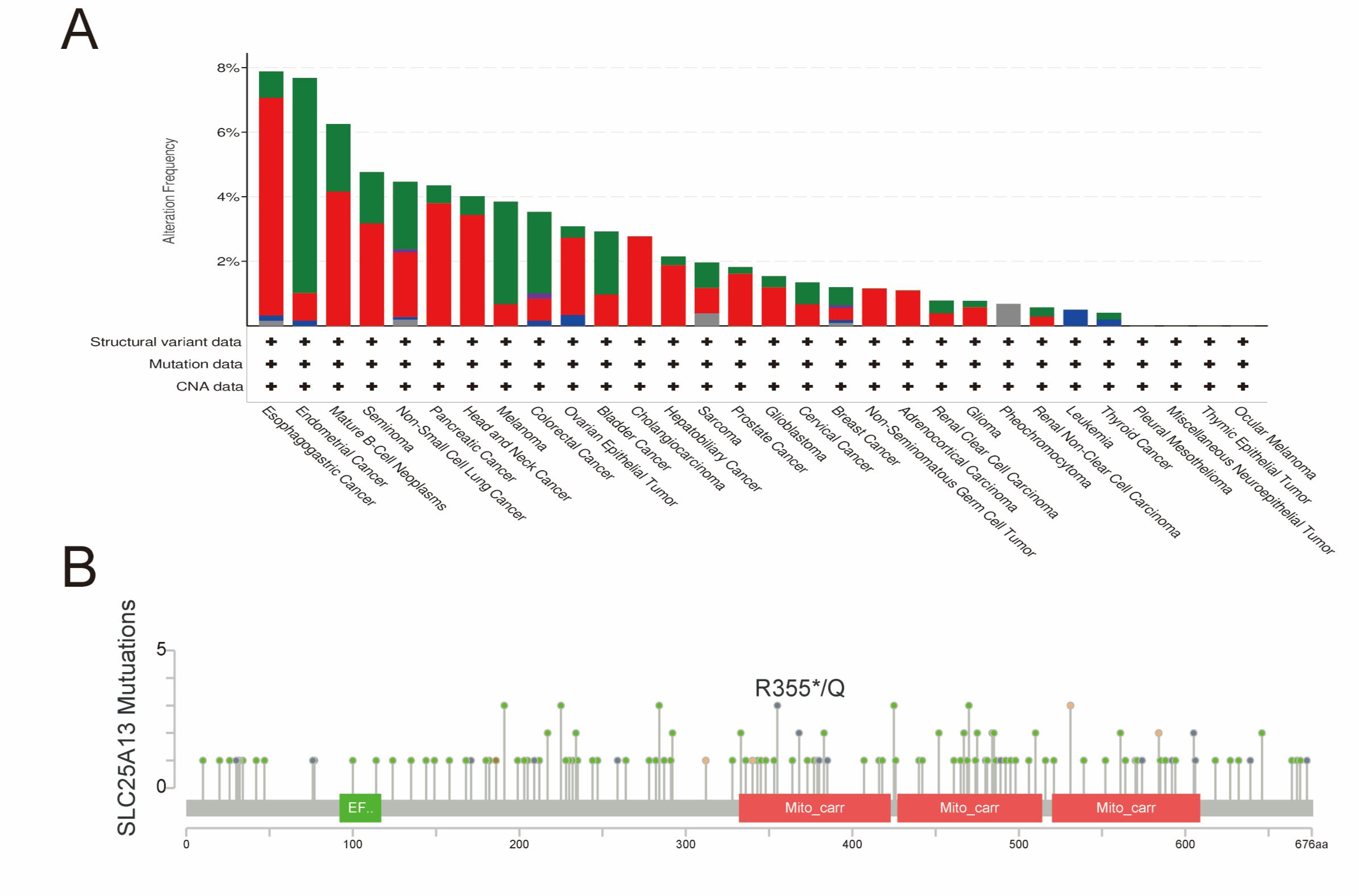

Supplement: Supplementary file 2 — Supporting File 2: advs75818‐sup‐0002‐supplement‐Extended‐Data.zip. [file ADVS-9999-e75818-s002.zip › supplement/Extended Data Fig. 1.jpg]

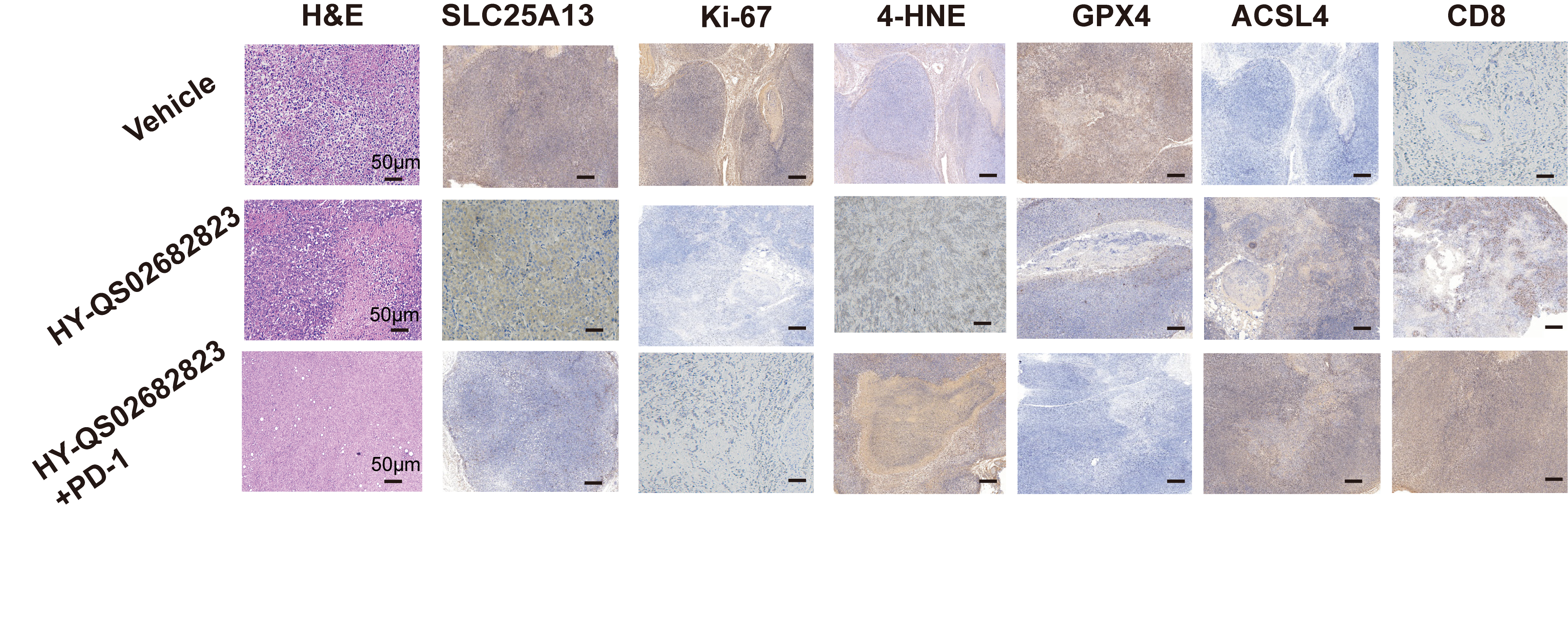

Supplement: Supplementary file 2 — Supporting File 2: advs75818‐sup‐0002‐supplement‐Extended‐Data.zip. [file ADVS-9999-e75818-s002.zip › supplement/Extended Data Fig. 10.tif]

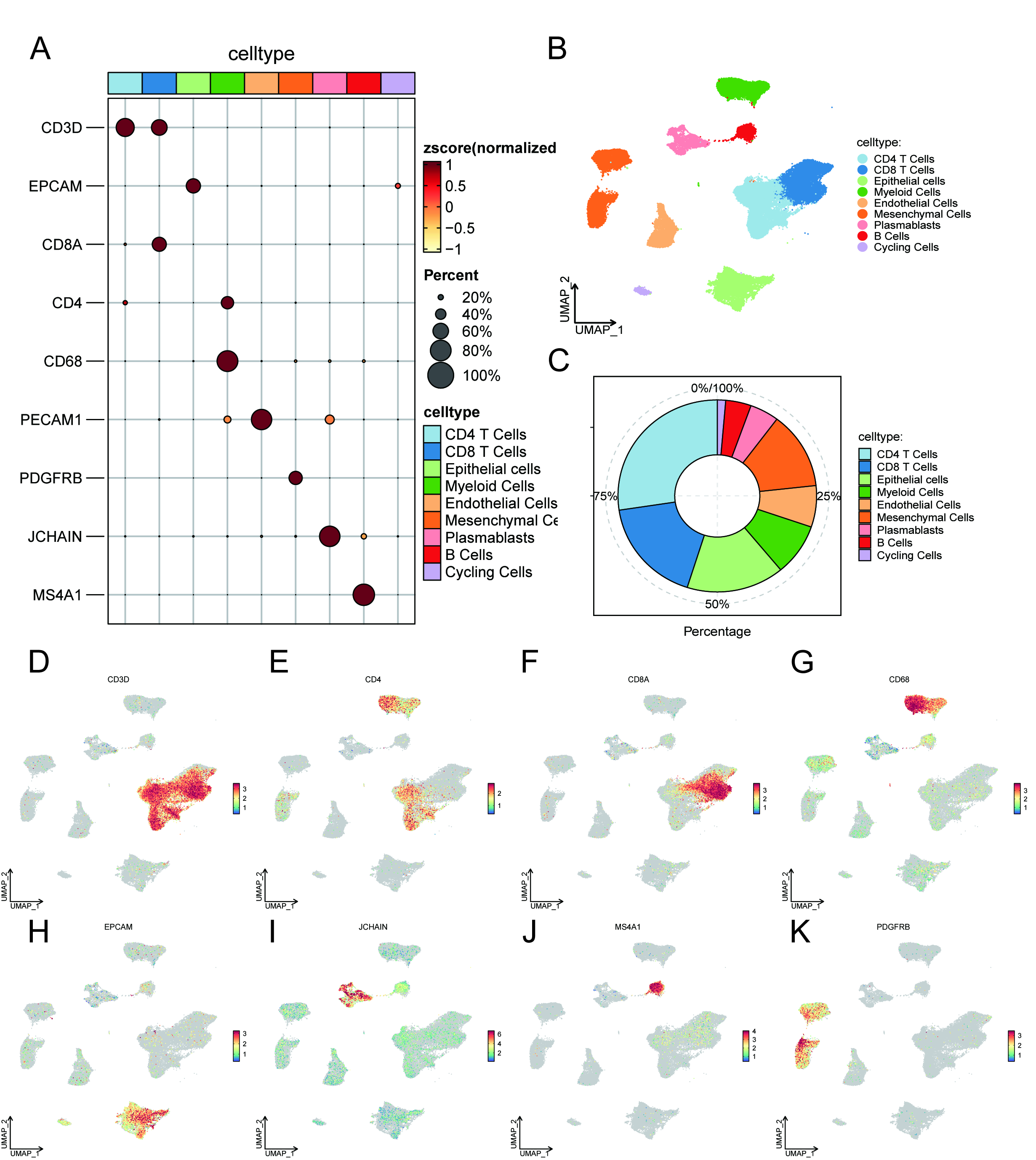

Supplement: Supplementary file 2 — Supporting File 2: advs75818‐sup‐0002‐supplement‐Extended‐Data.zip. [file ADVS-9999-e75818-s002.zip › supplement/Extended Data Fig. 2.tif]

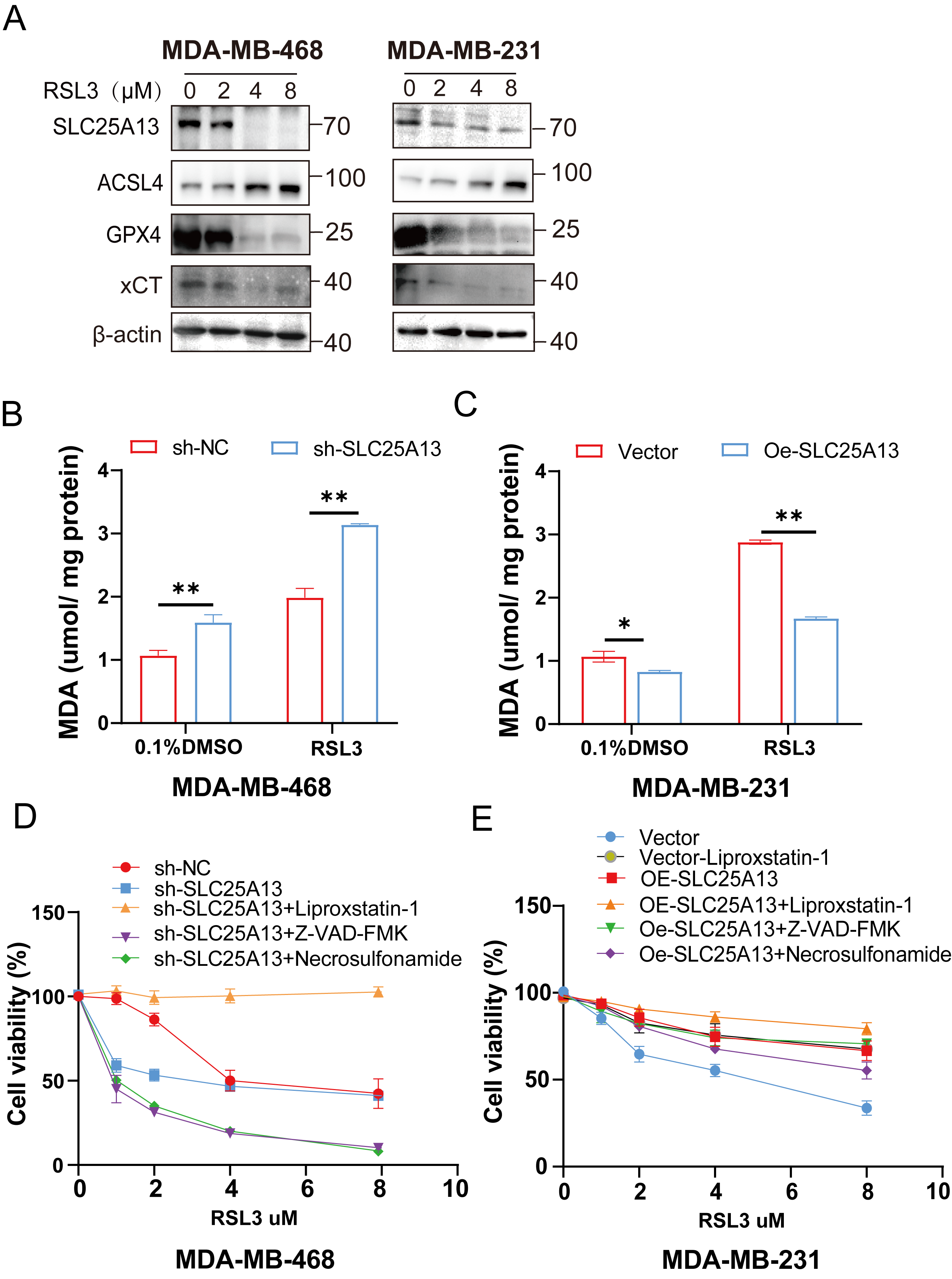

Supplement: Supplementary file 2 — Supporting File 2: advs75818‐sup‐0002‐supplement‐Extended‐Data.zip. [file ADVS-9999-e75818-s002.zip › supplement/Extended Data Fig. 3.tif]

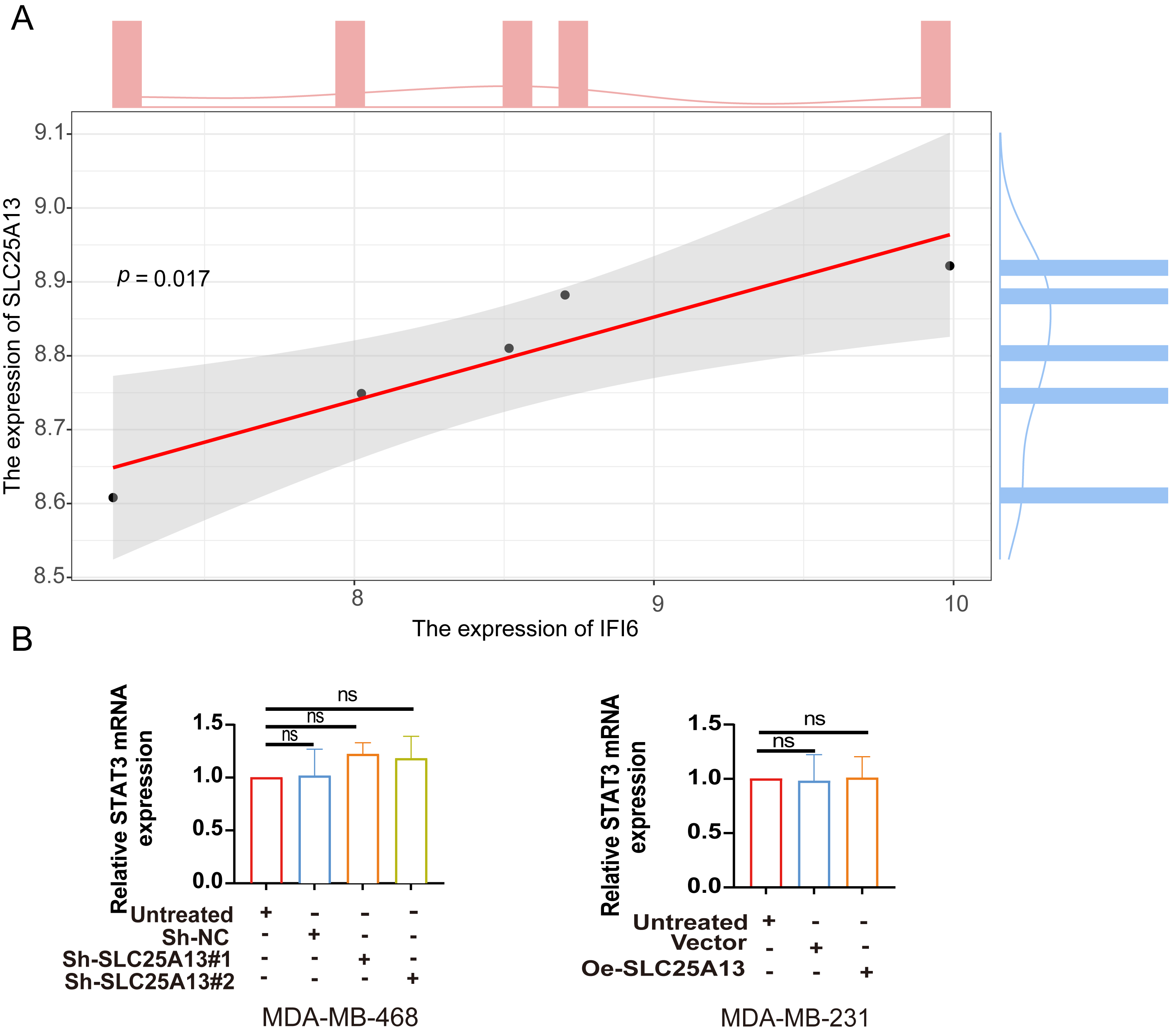

Supplement: Supplementary file 2 — Supporting File 2: advs75818‐sup‐0002‐supplement‐Extended‐Data.zip. [file ADVS-9999-e75818-s002.zip › supplement/Extended Data Fig. 4.tif]

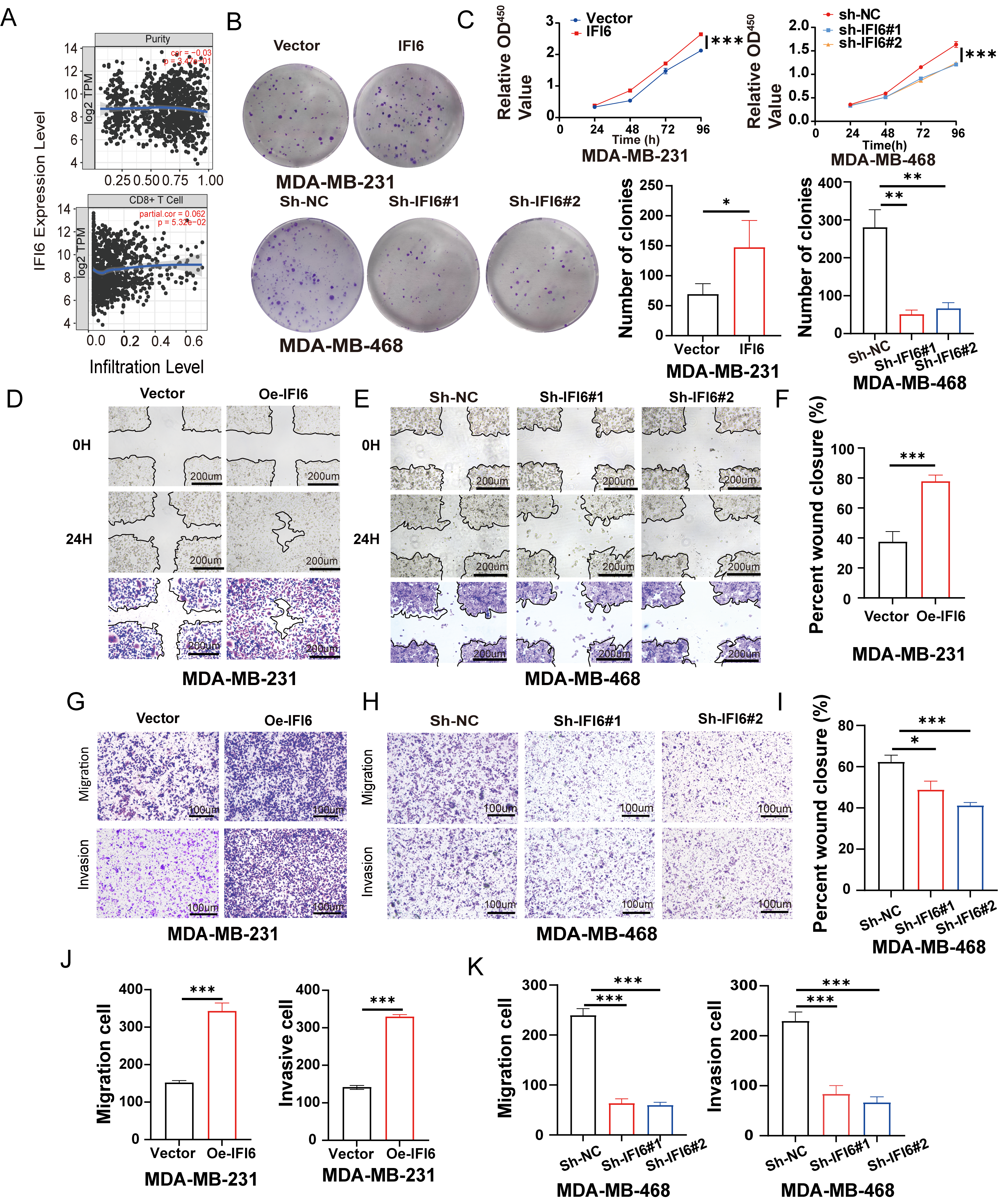

Supplement: Supplementary file 2 — Supporting File 2: advs75818‐sup‐0002‐supplement‐Extended‐Data.zip. [file ADVS-9999-e75818-s002.zip › supplement/Extended Data Fig. 5-.tif]

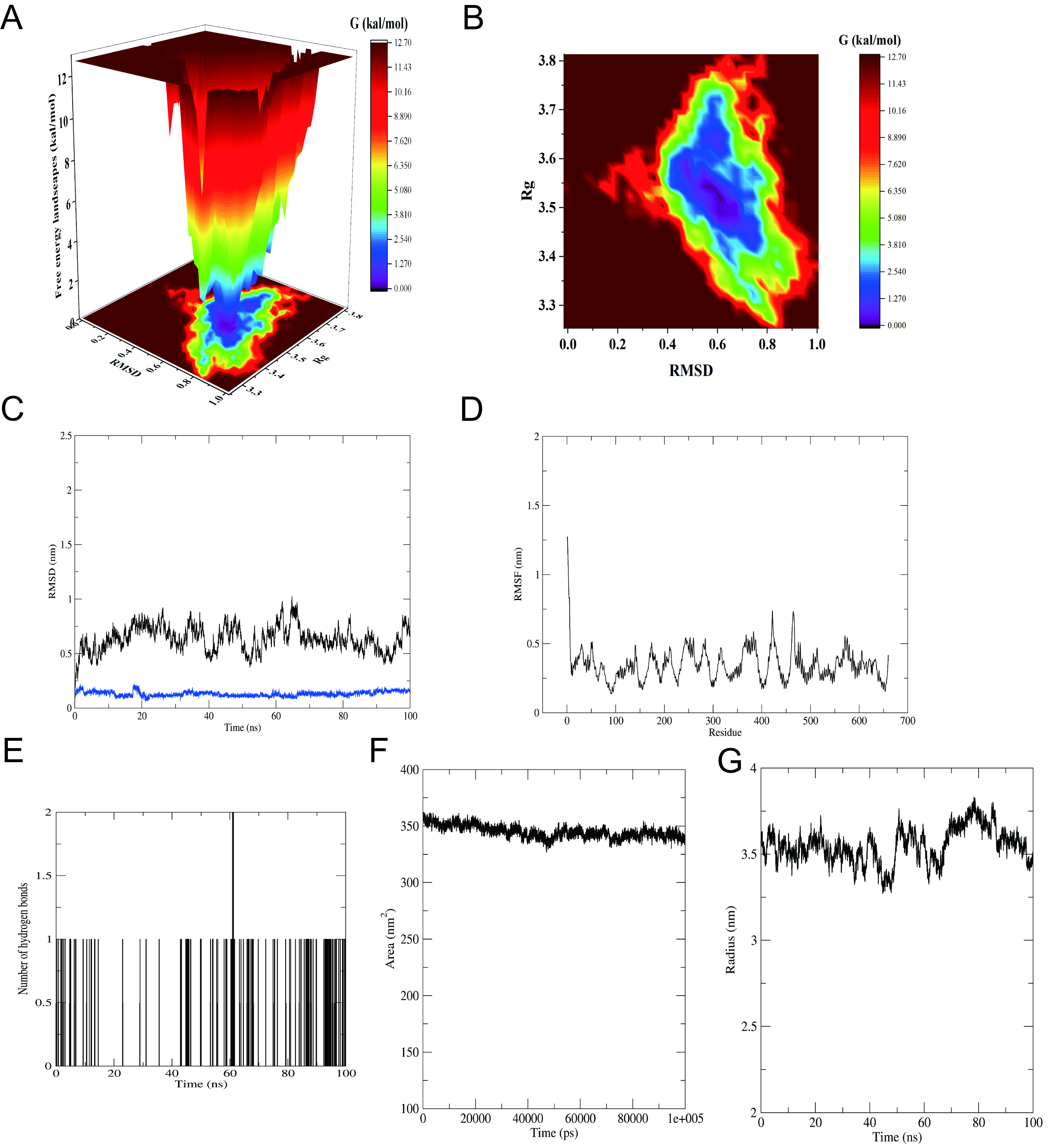

Supplement: Supplementary file 2 — Supporting File 2: advs75818‐sup‐0002‐supplement‐Extended‐Data.zip. [file ADVS-9999-e75818-s002.zip › supplement/Extended Data Fig. 6.tif]

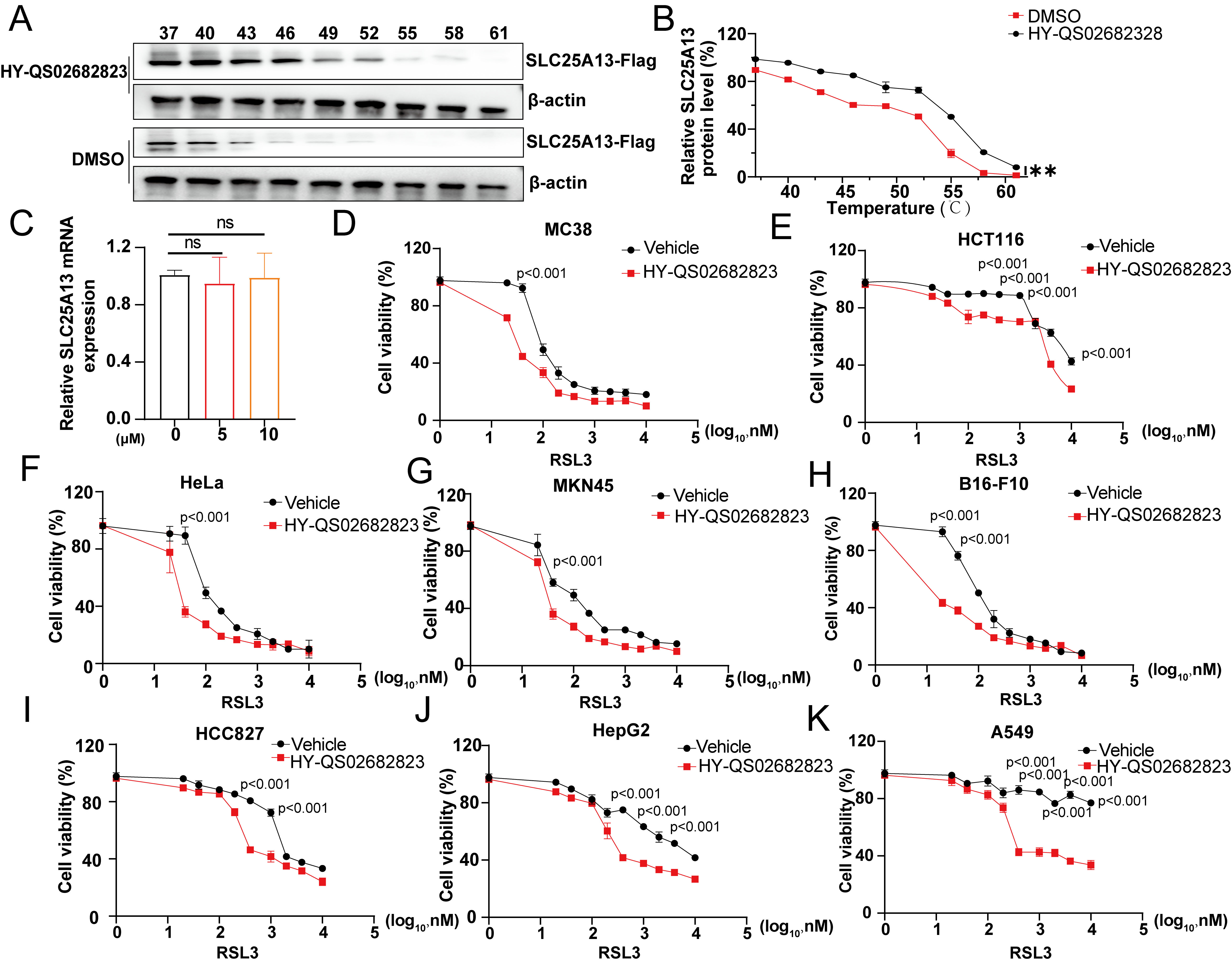

Supplement: Supplementary file 2 — Supporting File 2: advs75818‐sup‐0002‐supplement‐Extended‐Data.zip. [file ADVS-9999-e75818-s002.zip › supplement/Extended Data Fig. 7.tif]

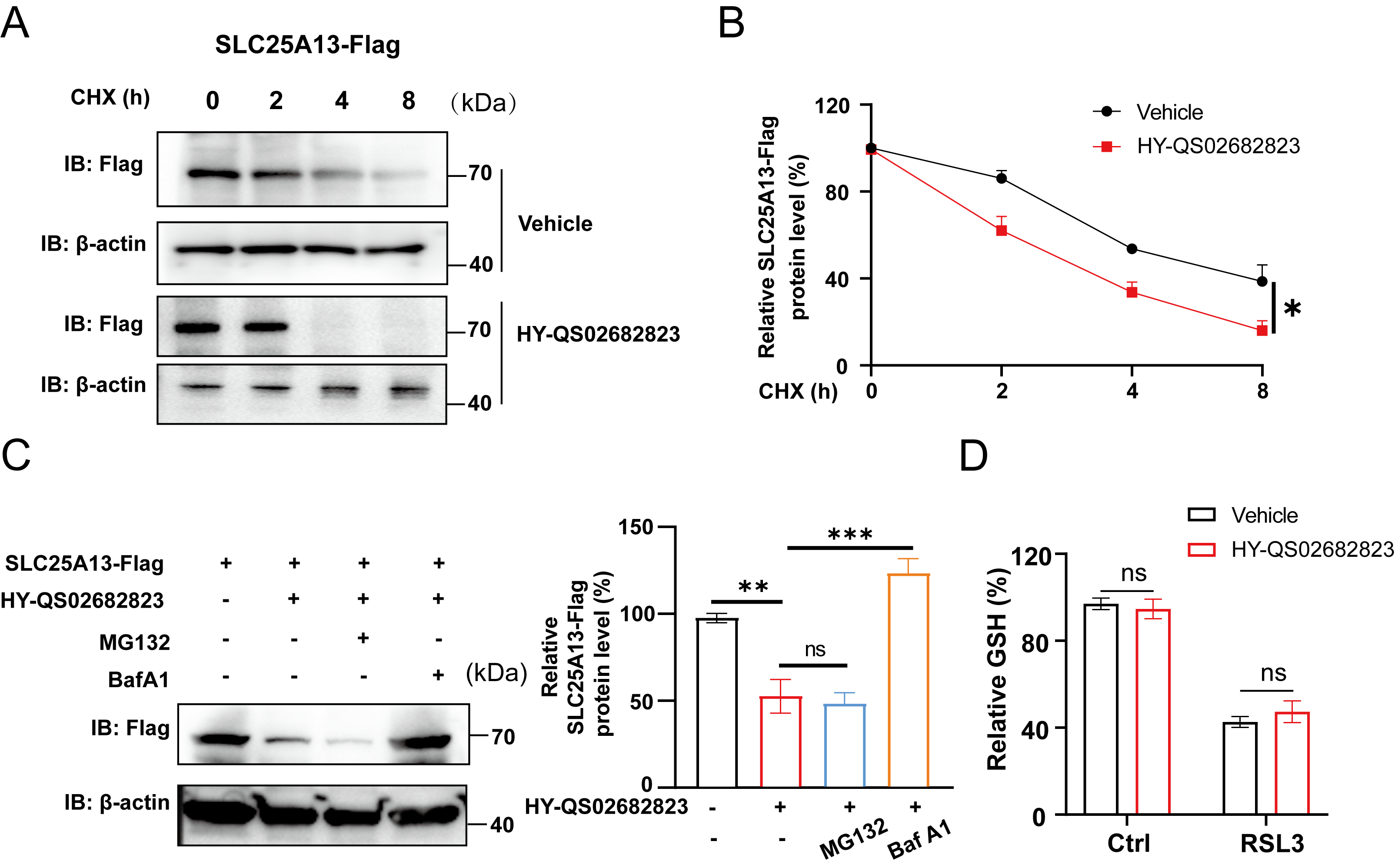

Supplement: Supplementary file 2 — Supporting File 2: advs75818‐sup‐0002‐supplement‐Extended‐Data.zip. [file ADVS-9999-e75818-s002.zip › supplement/Extended Data Fig. 8.tif]

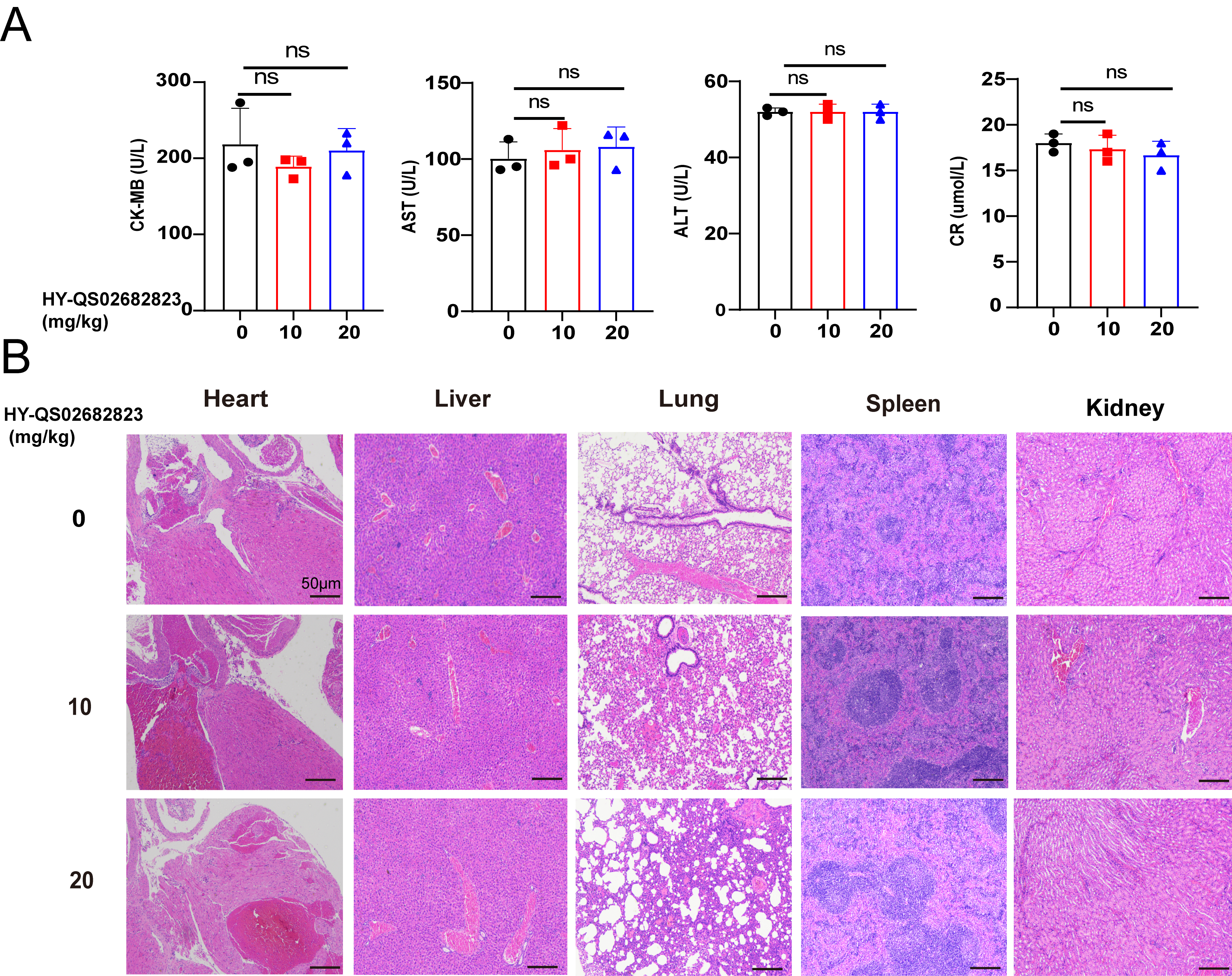

Supplement: Supplementary file 2 — Supporting File 2: advs75818‐sup‐0002‐supplement‐Extended‐Data.zip. [file ADVS-9999-e75818-s002.zip › supplement/Extended Data Fig. 9.tif]
